# Low versus high dose of antimony for American cutaneous leishmaniasis: A randomized controlled blind non-inferiority trial in Rio de Janeiro, Brazil

**DOI:** 10.1371/journal.pone.0178592

**Published:** 2017-05-30

**Authors:** Mauricio Naoto Saheki, Marcelo Rosandiski Lyra, Sandro Javier Bedoya-Pacheco, Liliane de Fátima Antônio, Maria Inês Fernandes Pimentel, Mariza de Matos Salgueiro, Érica de Camargo Ferreira e Vasconcellos, Sonia Regina Lambert Passos, Ginelza Peres Lima dos Santos, Madelon Novato Ribeiro, Aline Fagundes, Maria de Fátima Madeira, Eliame Mouta-Confort, Mauro Célio de Almeida Marzochi, Cláudia Maria Valete-Rosalino, Armando de Oliveira Schubach

**Affiliations:** 1 Laboratory for Leishmaniasis Surveillance, Evandro Chagas National Institute of Infectious Diseases, Oswaldo Cruz Foundation, Rio de Janeiro, Rio de Janeiro State, Brazil; 2 Department of Epidemiology and Quantitative Methods in Health, Sergio Arouca National School of Public Health, Oswaldo Cruz Foundation, Rio de Janeiro, Rio de Janeiro State, Brazil; 3 Clinical Epidemiology Laboratory, Evandro Chagas National Institute of Infectious Diseases, Oswaldo Cruz Foundation, Rio de Janeiro, Rio de Janeiro State, Brazil; 4 Department of Otorhinolaryngology and Ophthalmology, Faculty of Medicine, Federal University of Rio de Janeiro, Rio de Janeiro, Rio de Janeiro, Brazil; University of California Los Angeles, UNITED STATES

## Abstract

**Background:**

Although high dose of antimony is the mainstay for treatment of American cutaneous leishmaniasis (ACL), ongoing major concerns remain over its toxicity. Whether or not low dose antimony regimens provide non-inferior effectiveness and lower toxicity has long been a question of dispute.

**Methods:**

A single-blind, non-inferiority, randomized controlled trial was conducted comparing high dose with low dose of antimony in subjects with ACL treated at a referral center in Rio de Janeiro, an endemic area of *Leishmania (Viannia) braziliensis* transmission. The primary outcome was clinical cure at 360 days of follow-up in the modified-intention-to-treat (mITT) and per-protocol (PP) populations. Non-inferiority margin was 15%. Secondary objectives included occurrence of epithelialization, adverse events and drug discontinuations. This study was registered in ClinicalTrials.gov: NCT01301924.

**Results:**

Overall, 72 patients were randomly assigned to one of the two treatment arms during October 2008 to July 2014. In mITT, clinical cure was observed in 77.8% of subjects in the low dose antimony group and 94.4% in the high dose antimony group after one series of treatment (risk difference 16.7%; 90% CI, 3.7–29.7). The results were confirmed in PP analysis, with 77.8% of subjects with clinical cure in the low dose antimony group and 97.1% in the high dose antimony group (risk difference 19.4%; 90% CI, 7.1–31.7). The upper limit of the confidence interval exceeded the 15% threshold and was also above zero supporting the hypothesis that low dose is inferior to high dose of antimony after one series of treatment. Nevertheless, more major adverse events, a greater number of adverse events and major adverse events per subject, and more drug discontinuations were observed in the high dose antimony group (all p<0.05). Interestingly, of all the subjects who were originally allocated to the low dose antimony group and were followed up after clinical failure, 85.7% achieved cure after a further treatment with local therapy or low dose of antimony.

**Conclusions:**

Compared with high dose, low dose of antimony was inferior at the pre-specified margin after one series of treatment of ACL, but was associated with a significantly lower toxicity. While high dose of antimony should remain the standard treatment for ACL, low dose antimony treatment might be preferred when toxicity is a primary concern.

## Introduction

Leishmaniasis is a complex of vector-borne diseases caused by more than 20 species of the protozoan genus *Leishmania*, which are transmitted by a variety of female sandfly species [1–3]. It is considered one of the most neglected diseases in the world given its direct link to poverty and the glaring imbalance between its enormous healthcare burden and the limited resources invested in research and new drug development [4, 5]. An estimated 12 million people in 88 countries are infected with *Leishmania* worldwide and an additional 350 million are at risk of infection; one to two million new cases occur each year, while many more probably remain unreported [4, 5].

Cutaneous leishmaniasis (CL) is the most common form of the disease; it usually presents with a range of skin lesions, mainly painless ulcers, on exposed parts of the body, that may rarely leave lifelong scars, serious disability or spread to mucosal tissues [1, 3]. Approximately 75% of global cases of CL are confined to ten countries: Afghanistan, Algeria, Brazil, Colombia, Costa Rica, Ethiopia, the Islamic Republic of Iran, Peru, Sudan and the Syrian Arab Republic [6]. While Old World Leishmaniasis is mostly considered benign and self-limited [2, 3], in the New World it causes American tegumentary leishmaniasis (ATL), a syndrome named by Rabello in 1923 [7, 8], that comprises both CL and a variety of other manifestations, which include diffuse, disseminated and mucosal forms. In Brazil, about 20.000 cases of ATL are reported each year [9] with the predominant causative species being *Leishmania braziliensis* [1].

Antimony compounds have been used as the mainstay in the treatment of cutaneous leishmaniasis [1, 3, 10] since therapy with tartar emetic was first described by Gaspar Vianna in 1912 [11]. High dose of pentavalent antimony represents the current standard treatment for cutaneous leishmaniasis [3, 10, 12]. Specifically, World Health Organization (WHO) recommends pentavalent antimony (Sb^5+^) at a standard dose of 20 mg Sb^5+^/kg/day (high dose) administered by intramuscular or intravenous injection for 20 days with no ceiling dose [10]. The Brazilian Ministry of Health makes a similar recommendation, with a maximum daily dose of 1,215 mg Sb^5+^ [12]. Unfortunately, however, pentavalent antimony is not devoid of potentially serious side effects [3, 13]. It carries significant toxicity and morbidity [3, 13], and should be avoided in a subgroup of patients, which includes young children, elderly persons, pregnant women and those with hepatic, renal, or cardiovascular diseases [10, 12, 14].

On this account, a few observational studies have suggested that alternative antimonial treatment regimens such as low dose (5 mg Sb^5+^/kg/day), intermittent and intralesional therapies are safer, more cost-efficient and could be as effective as the standard high dose therapy for treating CL [15–24]. Other studies have shown that alternative antimonial regimens could be used in the aforementioned subgroup of patients with CL who, otherwise, would not get any treatment due to the high toxicity of the drugs [21].

Unsurprisingly, clinical trials to evaluate and optimize antimonial regimens are urgently warranted [25, 26], but have been almost non-existent to date. In our literature review, we identified only one small published trial [16] and another larger unpublished trial [26] that addressed the issue.

We designed this trial with the premise that treatment with a lower dose of antimony will avoid significant toxicity, provide less adverse events, but will nonetheless have non-inferior efficacy to the standard high dose antimony treatment.

## Methods

### Design overview

We compared 30 days of meglumine antimoniate, a pentavalent antimony compound, at a dose of 5 mg Sb^5+^/kg/day (low dose) with 20 days of meglumine antimoniate at a dose of 20 mg Sb^5+^/kg/day (high dose) up to a maximum of 1,215 mg Sb^5+^/day, in a phase III, randomized, controlled, single blind, non-inferiority trial, in subjects that acquired American cutaneous leishmaniasis (ACL) in Rio de Janeiro State, Brazil, an almost exclusive endemic area of *Leishmania (Viannia) braziliensis* transmission. The same lot of meglumine antimoniate (Glucantime^®^, Sanofi-Aventis, Suzano, Brazil), number 604898, was used in all of the subjects, provided by the Brazilian Ministry of Health.

### Participants

**Fig 1:**
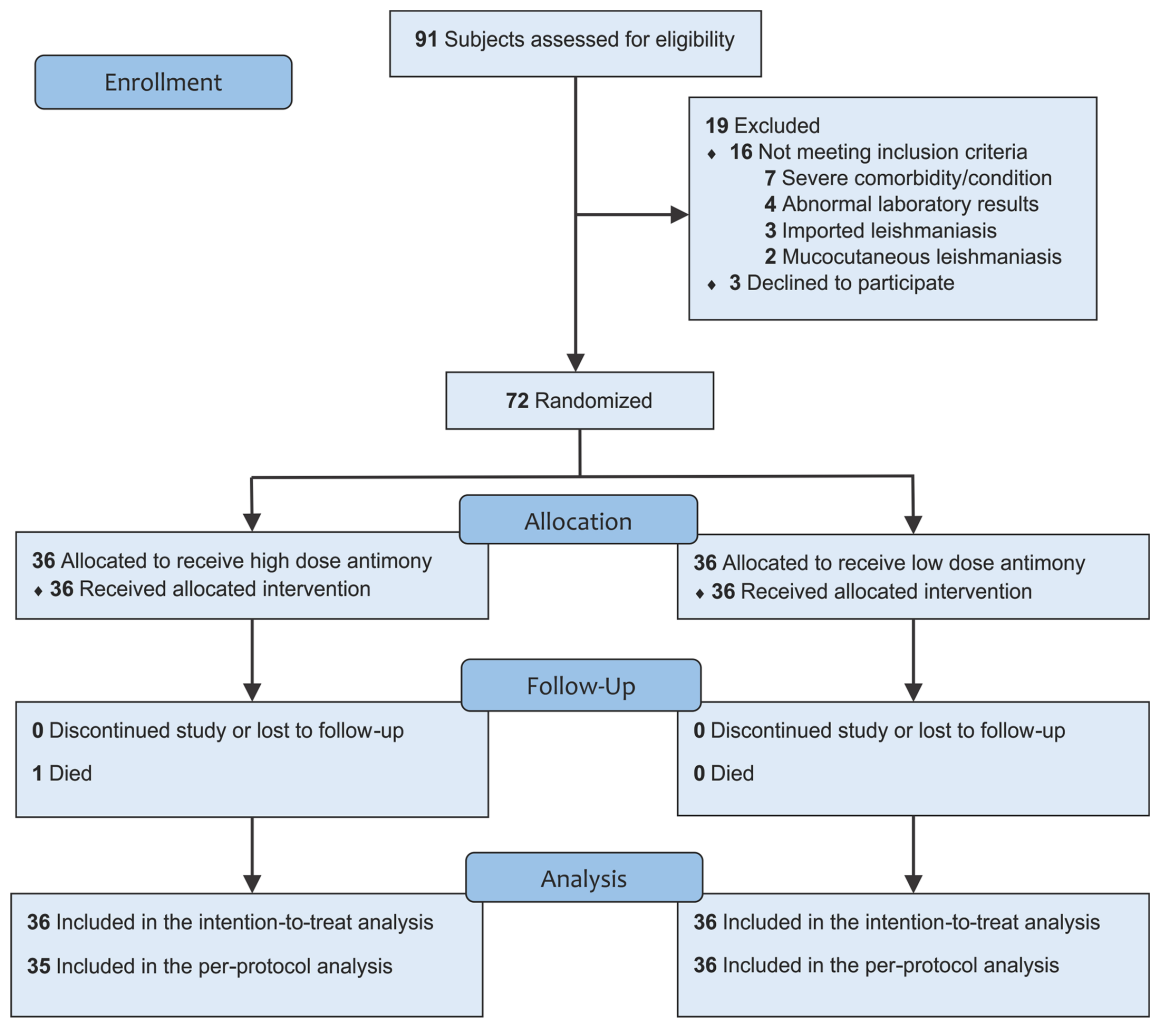
Flowchart of participants through each stage of the study “Low versus high dose of antimony for American cutaneous leishmaniasis.”

Fig 1 shows the flow chart for study participants. Subjects were consecutively recruited at the Evandro Chagas National Institute of Infectious Diseases (INI), Oswaldo Cruz Foundation (Fiocruz), on a voluntary basis. Eligible subjects were at least 13 years or older and had parasitological diagnosis of cutaneous leishmaniasis by at least one of the following methods: direct examination (scraping or imprint), histopathology, culture, immunohistochemistry or polymerase chain reaction (PCR). Reasons to exclude subjects comprised: prior treatment with meglumine antimoniate, concomitant mucosal leishmaniasis, lack of exposure in an endemic area of Rio de Janeiro State, women in reproductive age not using contraceptives, pregnancy, immunosuppressive therapy, ongoing treatment for tuberculosis or leprosy, presence of severe or worse changes in baseline clinical evaluation, presence of moderate or worse changes in baseline laboratory evaluation, presence of moderate or worse changes in baseline electrocardiographic evaluation or baseline corrected QT interval (cQT) >460 ms.

### Ethics statement

We obtained informed written consent from each subject before enrollment. Subjects under the age of 18 or who lacked capacity to consent took part in research only if written consent was given on their behalf by a legally authorised representative and written assent was obtained from participants. This consent procedure was approved by the ethics committee. The study was performed according to the ethical principles outlined in the Declaration of Helsinki and the guidelines from Brazilian National Health Council resolution 196/96 and 251/97 on research involving human subjects. Ethical approval was granted by the ethics committee at the Evandro Chagas National Institute of Infectious Diseases under number 0055.0.009.000–07 on October 17, 2007.

### Trial registration

The study was registered in ClinicalTrials.gov, number NCT01301924, after patient recruitment began. Clinical trial registration was delayed because the investigators did not have a trial registration policy in place at the time of this study. The authors confirm that all ongoing and related trials for this drug/intervention are registered.

### Randomization and masking

Randomization was performed by a statistician with no clinical involvement in the trial using a random allocation sequence generated by Epi Info, version 6.04d, in blocks of size 12. Eligible subjects were randomly allocated to receive one of the following regimens in a 1:1 ratio: 20 mg Sb^5+^/kg/day for 20 days (high dose), or 5 mg Sb^5+^/kg/day for 30 days (low dose). The allocation sequence was concealed in sequentially numbered, opaque and sealed envelopes until interventions were assigned. Envelopes were kept by an independent pharmacist in a safe deposit box at the pharmacy of INI. To ensure allocation concealment, after the written informed consents were obtained from eligible subjects, treatment was assigned by a second independent pharmacist at the pharmacy of INI.

Subjects were evaluated by clinicians and dermatologists in different rooms. While treatment assignment obviously could not be masked to subjects due to the intramuscular injections, they were instructed before each visit not to discuss any aspects of the treatment with the examiners. Clinicians involved in subjects’ enrollment and adverse events management were masked to lesion assessment and group assignment. Dermatologists who assessed the lesions were masked to group assignment, clinical data and adverse events. None of the investigators, apart from the independent pharmacist, was aware of treatment allocation. None of the investigators, apart from the trial statistician, had access to the randomization sequence. All clinical, dermatological, electrocardiographic (ECG) and laboratory assessments were performed masked with respect to the treatment allocation, which was not revealed until the database had been closed at the end of the trial. The treatment allocation and masking process was reviewed and checked by a Data Monitoring Committee. To test for adequate masking, clinicians and dermatologists were asked to guess group assignment at the end of the study. A second trial statistician responsible solely for undertaking the analyses was masked to the randomization sequence and to the treatment allocation until all analyses had been done.

### Procedures

Subjects underwent a detailed interview and health examination at enrollment, filled in a form containing clinical, socio-demographic information and assessment measures. Clinical assessment with enquiry about adverse events was done by a clinician every ten days during treatment, and every month thereafter for two months. Samples for routine laboratory tests were taken at baseline and at each clinical assessment. Clinical, ECG and laboratory adverse events were graded using a toxicity grading scale adapted from the Division of AIDS Table for Grading of Severity of Adult and Pediatric Adverse Events [27]. Although the antimony ampoules were administered in local healthcare centers, compliance was ascertained by a pharmacist through interviewing, counting of the remaining ampoules and local nurse signature’s verification in the study medication card. Satisfactory compliance was defined as completion of prescribed treatment or as missing less than 10% of treatment within the specified duration. Lesions, ulcers and scars were assessed and measured by dermatologists every ten days for two months, then every month for three months, then every three months until the end of one year, then every six months until the end of two years, and then once a year. Standardized photographs were taken at baseline and at each evaluation and were also used by dermatologists to follow lesions over time. Subjects were further assessed to rule out mucosal lesions, through videofiberoptic nasolaryngoscopy, performed by an Ear, Nose and Throat (ENT) specialist in the first medical visit, then every two months until the end of one year, then every six months until the end of two years, then once a year. Approaches used to prevent missing data and drop outs included: a tracking system containing standard subject and supplementary information (telephone numbers, e-mail and physical addresses of relatives, neighbors, friends or primary care workers), “default reminders” (telephone calls, telephone messages or letters a day after a missed visit) and transport reimbursement. We also performed quality control of forms and routine verification of missing data.

### Outcome measures

The primary outcome was clinical cure at 360 days of follow-up in the modified intention-to-treat (mITT) and per-protocol (PP) populations, defined as: epithelialization within 120 days, scarring within 360 days, absence of clinical worsening, absence of relapse and no appearance of mucosal lesion.

**Fig 2:**
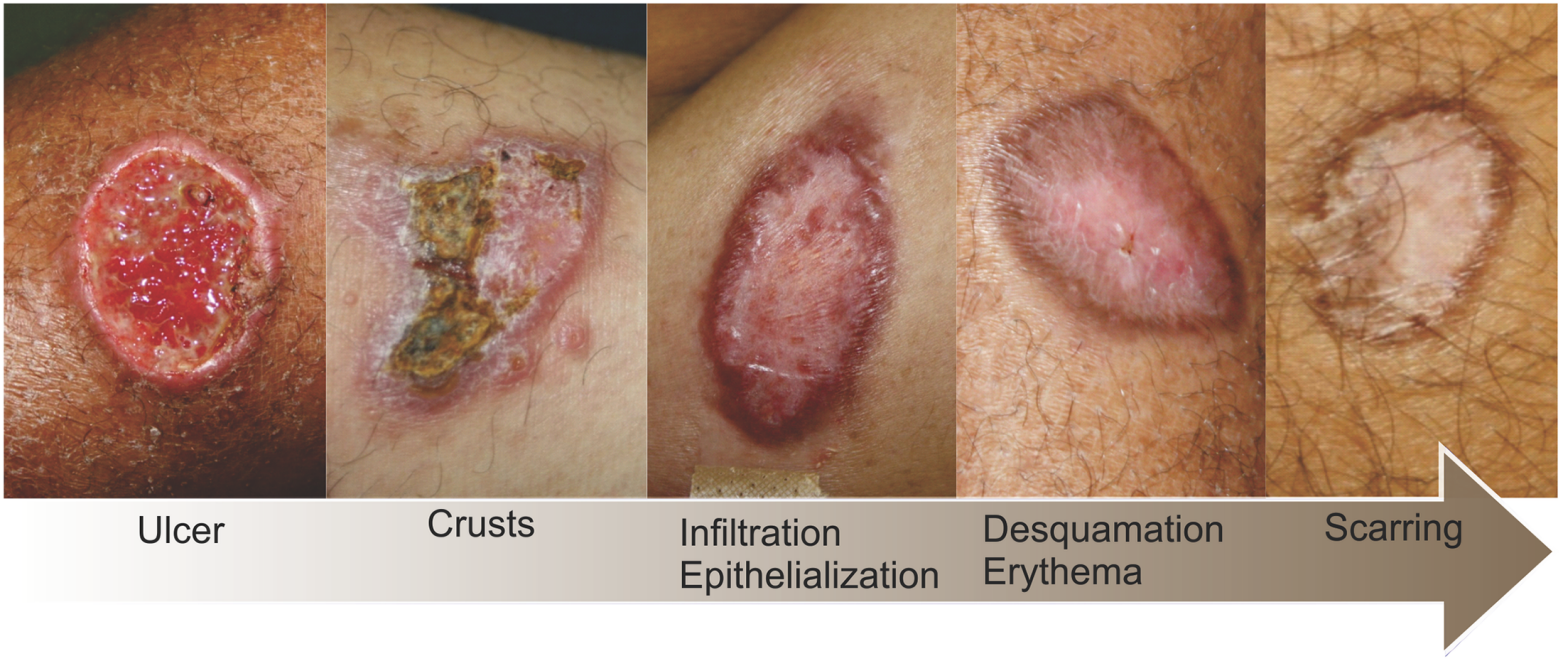
Different stages of the wound healing process in American cutaneous leishmaniasis.

Fig 2 illustrates a model of different phases of the wound healing process in ACL, from a typical leishmanial ulcer to a healed scar, and was included for reference. Epithelialization was defined as complete wound closure without any erosion. Scarring was assessed at each visit and it was defined as the presence of the following criteria: complete healing of all lesions characterized by complete epithelialization and absence of crusts, infiltration, desquamation, or erythema. Clinical worsening was defined as deterioration of presenting signs or appearance of satellite lesions. Relapse was defined as the reappearance of inflammatory signs in the scar or development of new cutaneous lesions in other locations after scarring was established. Appearance of mucosal lesion was defined as the observation of any mucosal lesion and subsequent confirmation of mucosal leishmaniasis by at least one of the following laboratory methods obtained through lesion biopsy: *Leishmania* parasite detection using PCR, parasite isolation by culture, or identification of amastigotes by routine histopathological exam, imprint or immunohistochemical analysis. Early failure was defined as failure caused by lack of response, appearance of mucosal lesions or clinical worsening within 120 days of follow-up. Failure was defined as inability to achieve clinical cure. Time to failure was defined as the interval between treatment initiation to the first occurrence of failure.

Secondary outcomes were the occurrence of epithelialization, adverse events (AE), major adverse events and drug discontinuations. Major adverse events were defined as presence of severe or worse changes in clinical evaluation, presence of moderate or worse changes in laboratory evaluation, presence of moderate or worse changes in electrocardiographic evaluation or corrected QT interval (cQT) >460 ms.

Subjects received alternative therapies according to clinician’s preferences and standard local practices if they had not achieved clinical cure or were unable to tolerate the allocated treatment.

The mITT analysis included all patients who received at least one dose of meglumine antimoniate. Missing data in mITT analysis were handled using the last-observation-carried-forward method pre-specified by the protocol. We compared this imputation method to complete case analysis and the expectation-maximization algorithm to test the robustness of our findings. The PP analysis was defined on a by-visit basis and included those mITT patients who met the entry criteria, received more than 90% of study medication and had an observed outcome for that visit time in regard to clinical response.

### Statistical analysis

Our hypotheses for sample size calculations were based on historical data [3, 15, 28–30] and findings of a previous study performed in Rio de Janeiro State, Brazil [15]. We assumed that the treatment efficacy of standard high dose antimony would be 95%, the efficacy difference of standard high dose versus low dose treatment would not exceed 15% and the hypothetical failure rate on placebo would be 90%. The largest clinically acceptable margin (M1) was assumed to be 85.5%, which represents the difference in clinical cure rate between standard high dose antimony and placebo (S1 Appendix). A non-inferiority margin (M2) of 15% was determined on clinical judgement, based on a panel discussion by clinical investigators and the study statistician, as well as on statistical analysis using historical data [3, 15, 28–30] and a multistep method consistent with U.S. Food and Drug Administration draft guideline recommendations [31] (S1 Appendix). This non-inferiority margin would be sufficient to preserve 82.5% of M1 and ensure that the conclusion of non-inferiority was consistent with the analysis. An assay sensitivity analysis was accordingly performed to study different possible scenarios of M1, by decreasing the effectiveness of standard high dose antimony treatment from 95% to 80% and increasing the failure rate on placebo from 75% to 95% (S1 Appendix). To calculate the sample size, we have used estimates from the largest clinically acceptable margin derived from historical data [3, 15, 28–30]. Sample size calculations were inflated to allow for the possibility of up to a 10% withdrawal rate from the study. With these assumptions, a statistical power (1 –β) of 80%, and α level of 5% (one-sided test), a total of 72 patients would be required to determine non-inferiority.

The study was designed and powered as a non-inferiority trial on the primary endpoint at day 360. The analyses for the primary and secondary endpoints were on a mITT basis, supplemented by PP analysis of the primary endpoint. The hypothesis is presented as follows: H0: P(high)–P(low) ≥ Δ versus H1: P(high)–P(low) < Δ; where P(high) is the cure rate observed in the high dose group, P(low) is the cure rate observed in the low dose group and Δ defines the margin of non-inferiority. Non-inferiority of low dose compared with high dose of antimony would be claimed if the upper limit of the one-sided 95% confidence interval (CI) for the risk difference between treatments did not exceed the pre-specified margin of 15%, equivalent to a one-sided test with an alpha value of 0.05. Categorical data was compared using the Pearson’s chi-squared test or Fisher’s exact test, as appropriate. Normal distribution of the data was tested using Shapiro-Francia W’ and Kolmogorov-Smirnov tests. Normal distribution was considered if p-values were above 0.05. Continuous data with normal distribution was compared with the Student’s t test; whereas the Mann-Whitney, the Kruskal Wallis or the Wilcoxon signed-rank tests were applied to variables with non-normal distribution, as appropriate. A pre-specified subgroup interaction analysis by age, gender and race was performed to evaluate potential effect modifiers between modalities and outcomes. For subjects experiencing clinical failure, subsequent treatments were recorded and compared. Statistical significance of the observed differences was considered at a probability of type 1 error less than 0.05. In order to find the upper limit of the one-sided 95% CI, two-sided confidence intervals were calculated at the 90% level for the primary analysis since the one-sided 95% CI and the two-sided 90% CI share the same upper limit [32]. All the other confidence intervals were two-sided computed at the 95% level, all p-values reported were two-sided. All statistical analysis was performed using SPSS version 19.0 (IBM Corp, Armonk, NY, USA) and Stata version 12.0 (Stata Corp, College Station, TX, USA).

### Role of the funding source

The authors have declared that no competing interests exist. No commercial entity had any role in the study. This study is supported in part with grants approved by the Brazilian National Council for Scientific and Technological Development (CNPq), Carlos Chagas Filho Foundation for Research Support of Rio de Janeiro State (FAPERJ) and the Brazilian Ministry of Health. The Brazilian Ministry of Health provided the meglumine antimoniate ampoules. The funders had no role in the study design, data collection and analysis, decision to publish or preparation of the manuscript. All authors made the decision to submit the report for publication and guarantee the veracity and completeness of the data and their analyses.

## Results

### Study subjects

Between October 2008 and July 2014, 91 subjects were screened for the study; of whom, 72 with parasitologically confirmed diagnosis of ACL were enrolled, 36 were randomly allocated to the high dose group and 36 were allocated to the low dose group (Fig 1). Date range for participant follow-up was from October 2008 to May 2016. *Leishmania* parasites were isolated and identified through species characterization by multilocus enzyme electrophoresis (MLEE), performed according to a previously described procedure [33], from 75.0% of subjects (54/72) from biopsy samples obtained at the baseline visit. All the samples analyzed by MLEE in this study (54/54) were identified as *Leishmania (Viannia) braziliensis*. Table 1 shows the baseline and clinical characteristics of the mITT population. Baseline demographic and clinical characteristics were well balanced between the groups, and no statistically significant difference was found in regard to age, gender, race, place of geographical residence or underlying disease. There were a few statistically nonsignificant but noteworthy imbalances: the high dose group had a lower median age, a greater percentage of subjects with diabetes and lesions located in lower limbs. No serious protocol violation was observed in the study. Follow-up occurred at an average of 3.78 years (95% CI, 3.36 to 4.19) and was similar in both groups. One death occurred in the high dose group and no subject was lost to follow-up; mITT and PP populations were identical.

**Table 1:** Baseline demographic and clinical characteristics of the modified intention-to-treat population.

Baseline variables	Low dose antimony group (n = 36)	High dose antimony group (n = 36)
**Demographic factors**		
**Site, n (%)**		
** Metropolitan**	25 (69.4)	22 (61.1)
** Lowland coast**	4 (11.1)	5 (13.9)
** Central region**	2 (5.6)	4 (11.1)
** North region**	4 (11.1)	4 (11.1)
** South region**	1 (2.8)	1 (2.8)
**Male gender, n (%)**	25 (69.4)	26 (72.2)
**Age, years**	44.0 [25.5–51.5]	38.0 [24.0–55.0]
** Age groups, n (%)**		
** 13–20 years**	6 (16.7)	4 (11.1)
** 21–35 years**	8 (22.2)	13 (36.1)
** 36–50 years**	11 (30.6)	8 (22.2)
** >50 years**	11 (30.6)	11 (30.6)
**Race**		
** White**	15 (41.7)	16 (44.4)
** Black**	5 (13.9)	3 (8.3)
** Mulatto**	16 (44.4)	17 (47.2)
**Body weight, kg**	73.7 (14.9)	72.0 (14.8)
**Height, cm**	169.5 (9.8)	166.4 (9.0)
**Corporal mass index, kg/m**^**2**^	25.8 (5.6)	26.0 (5.0)
**Level of education**		
**Schooling, years**	6.9 (3.8)	6.3 (3.8)
**Type of occupation, n (%)**		
** Sales and service workers**	12 (33.3)	13 (36.1)
** Household workers**	7 (19.4)	6 (16.7)
** Students**	7 (19.4)	4 (11.1)
** Agricultural labourers**	4 (11.1)	6 (16.7)
** Construction labourers**	4 (11.1)	3 (8.3)
** Business and administration professionals**	1 (2.8)	3 (8.3)
** Science and engineering professionals**	1 (2.8)	1 (2.8)
**Clinical factors**		
**Illness duration before treatment, weeks**	13.0 [9.0–20]	12.0 [10.0–17.0]
**Hypertension, n (%)**	11 (30.6)	13 (36.1)
**Diabetes, n (%)**	1 (2.8)	5 (13.9)
**Alcohol drinking, n (%)**	13 (36.1)	13 (36.1)
**Current smoking, n (%)**	7 (19.4)	6 (16.7)
**Intestinal parasites, n (%)**	4 (11.1)	6 (18.2)
**No. of lesions per patient**	1.0 [1–2.5]	1.0 [1.0–1.0]
**Feature of the main lesion—ulcerated, n (%)**	34 (94.4)	33 (91.7)
**Site of the main lesion—lower limbs, n (%)**	11 (30.6)	18 (50.6)
**Lymph node involvement, n (%)**	9 (25.0)	11 (30.6)
**Mean diameter of the main lesion, mm**	33.8 (10.1)	31.6 (17.3)
**Mean diameter of the main ulcer, mm**	22.7 (8.1)	19.9 (7.8)
**Montenegro skin test, mm**	14.1 (8.8)	18.2 (8.5)
**Montenegro skin test positivity, n/N (%)**	29/33 (87.9)	29/30 (96.7)
**Positive culture for Leishmania in skin biopsy, n (%)**	32 (94.1)	31 (91.2)
**ELISA optic density/cut off ratio**	2.3 [1.6–3.5]	2.3 [1.9–3.1]
**ELISA positivity, n (%)**	29 (80.6)	32 (88.9)

Values are expressed as a number (percentage), mean (standard deviation) or median [25-75^th^ percentiles].

### Outcomes

**Fig 3:**
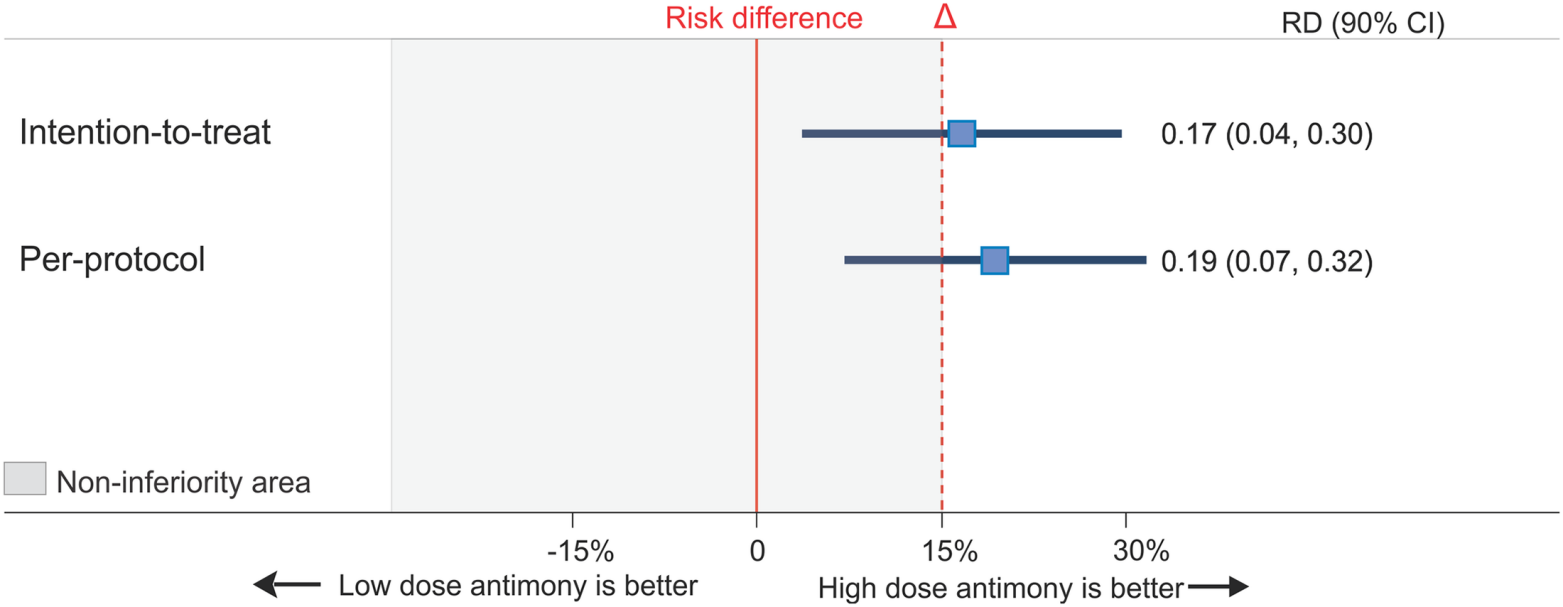
Non-inferiority plot of low dose antimony versus high dose antimony for American cutaneous leishmaniasis. Data are shown as point estimates and 90% confidence interval (CI) for absolute risk difference in clinical cure for intention-to-treat and per-protocol populations. Non-inferiority can be established if confidence intervals are within the prespecified boundary for non-inferiority (Δ).

The cumulative proportion of subjects in whom the primary outcome, clinical cure, occurred was 77.8% in the low dose group and 94.4% in the high dose group for the mITT analysis after one series of treatment, with a difference between the groups of 16.7% (90% CI, 3.7 to 29.7). For the PP analysis, the primary outcome occurred in 77.8% of subjects in the low dose group and 97.1% in the high dose group, with a difference of 19.4% (90% CI, 7.1 to 31.7) (Table 2). These confidence intervals sat wholly above zero and did include the prespecified margin of 15%, meaning that the low dose group was consistently inferior to the high dose group after one series of treatment (Fig 3). Subjects treated in the high dose group had a relative risk of clinical cure of 1.21 in the ITT (90% CI, 1.03 to 1.43) and 1.25 in the PP population (90% CI, 1.07 to 1.46) when compared to the low dose group. Epithelialization within 120 days was also significantly increased in the high dose group in comparison to the low dose group (Table 2). In regard to the subgroup analysis, no significant interaction was found between the primary outcome and age, gender or race.

**Table 2:** Primary and secondary outcomes.

	Low dose group		High dose group		Absolute rate difference	Relative risk	Relative risk increase	p-value
	n/N	% (95% CI)	n/N	% (95% CI)	% (95% CI)	(95% CI)	%	
**Primary outcome**								
Clinical cure: Intention-to-treat	28/36	77.78 (60.88, 88.73)	34/36	94.44 (79.61, 98.67)	16.67 (-∞, 29.68^a^)	1.21 (-∞, 1.43^a^)	21.43	0.04*
Clinical cure: Per-protocol	28/36	77.78 (60.87, 88.73)	34/35	97.14 (81.36, 99.62)	19.37 (-∞, 31.67^a^)	1.25 (-∞, 1.46^a^)	24.90	0.03^¶^
**Secondary outcome**								
Epithelialization at day 120	28/36	77.78 (60.88, 88.72)	34/36	94.44 (79.61, 98.67)	16.67 (1.16, 32.17)	1.21 (1.00, 1.47)	21.43	0.04*
Any adverse event	35/36	97.22 (81.82, 99.63)	35/36	97.22 (81.82, 99.63)	0.00	1.00	0.00	1.00^¶^
No. of adverse events per subject	5.28	(3.84, 6.72)	8.06	(6.29, 9.83)	-		-	0.02^&^
Major adverse events	12/36	33.33 (19.65, 50.55)	25/36	69.44 (52.23, 82.53)	36.11 (14.58, 57.64)	2.08 (1.25, 3.47)	108.33	<0.01*
No. of major adverse events per subject	0.56	(0.17, 0.94)	1.89	(1.11, 2.67)	-	-	-	<0.01^&^
Overall drug discontinuations^b^	7/36	19.44% (9.34, 36.13)	23/36	63.89% (46.72, 78.11)	44.44% (24.11, 64.77)	3.29 (1.62, 6.68)	228.57%	<0.01*

CI, confidence interval.

P-values were calculated using the:

* Pearson’s chi squared test,

^¶^ Fisher’s exact test, or

^&^ Mann-Whitney U test;

p-values <0.05 were considered statistically significant.

^a^ One-sided 95% CI, the upper limit of a 95% one-sided Cl is equivalent to the upper limit of a two-sided 90% CI.

^b^ Overall drug discontinuations corresponds to temporary and permanent drug discontinuations.

In total, six out of nine subjects who did not achieve cure after the first series of treatment received subsequent therapies (S2 Appendix). Four of them originally allocated to the low dose antimony group achieved cure after a further treatment with intralesional or low dose antimony. One subject failed retreatment with intralesional antimony and finally cured after liposomal amphotericin B. One subject originally allocated to the high dose antimony group cured after liposomal amphotericin B. Two subjects were cured after biopsy excision of the lesion and did not require further treatment. One subject had clinical failure confirmed by biopsy, but did not return for treatment. Of all the subjects who were originally allocated to the low dose antimony group and were followed up after clinical failure, 85.7% achieved cure after a further treatment with local therapy (i.e lesion excision by biopsy or intralesional antimony) or low dose of antimony.

Mean time to failure was 135.0 days (95% CI, 28.8 to 241.2 days). All nine failures occurred due to clinical worsening, four of them were classified as early failures. In the follow-up time, neither relapse nor appearance of mucosal lesions was described.

### Safety

Of all subjects, 97.2% had at least one adverse event. The proportion of subjects with any adverse event was not different between the low dose and the high dose groups. However, the number of adverse events (p = 0.02) and major adverse events (p<0.01) per subject was greater in the high dose group (Table 2).

Among subjects in the high dose group, the risk of presenting major adverse events, as compared to those in the low dose group, increased by 108.3% (relative risk, 2.08; 95% CI, 1.25 to 3.47; p<0.01).

The proportion of subjects with laboratory, major laboratory and major clinical adverse events was significantly larger in the high dose group than in the low dose group, with a 36.4% increase in risk of laboratory (83.3% versus 61.1%; relative risk 1.36; 95% CI, 1.01 to 1.84; p = 0.04), 500.0% increase in risk of major laboratory (50.0% versus 8.3%; relative risk, 6.00; 95% CI; 1.94 to 18.60; p<0.01) and 160.0% increase in risk of major clinical adverse events (36.1% versus 13.9%; relative risk, 2.60; 95% CI, 1.04 to 6.54; p = 0.03). Additional pre-specified analyses showed that all adverse and major adverse events rate differences in clinical, ECG and laboratory parameters were in the same direction with high dose group subjects consistently more likely to experience adverse and major adverse events at higher rates than low dose group subjects.

**Fig 4:**
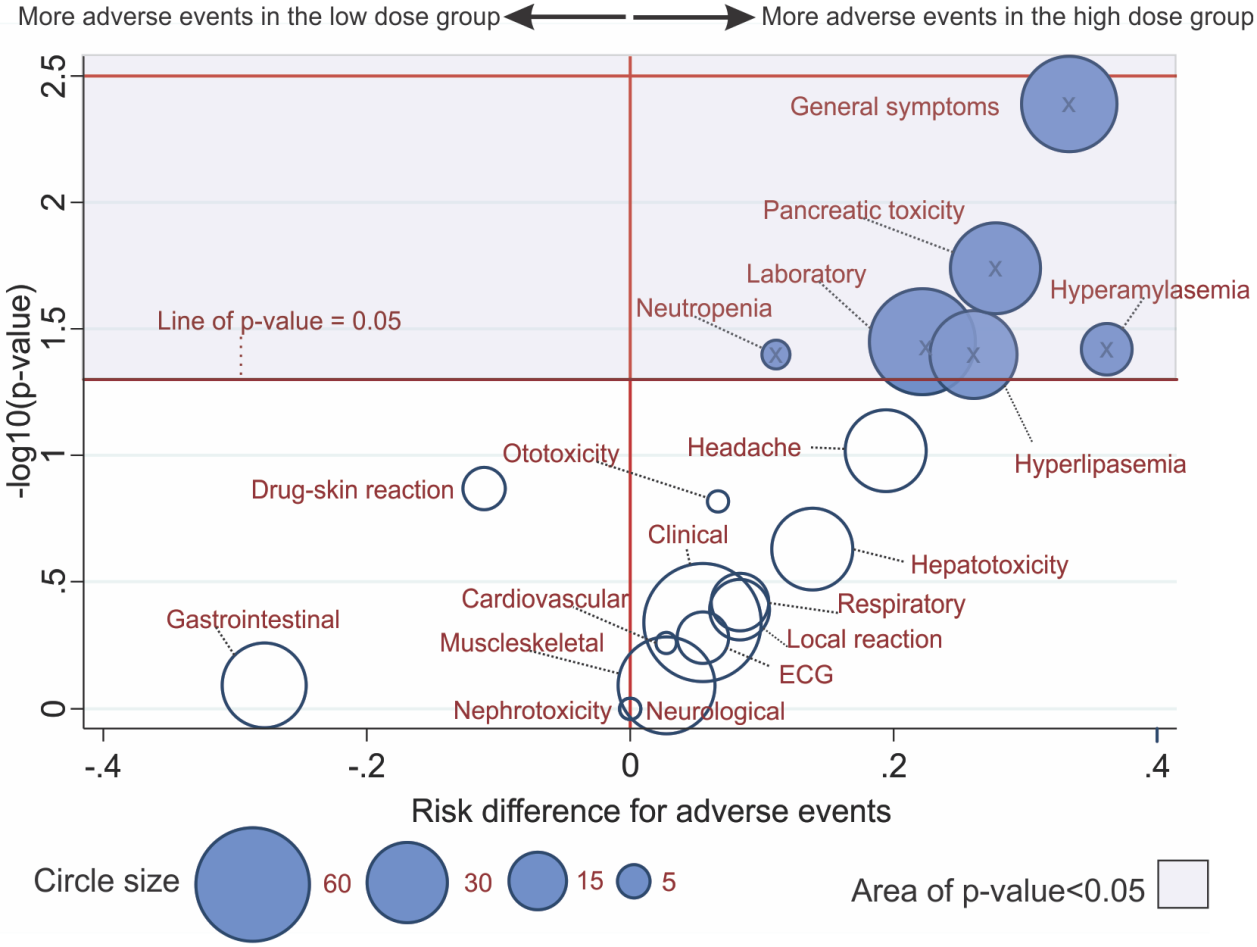
Bubble plot of -log10(Raw p-value) by risk difference with a two-sided 95% confidence interval sized by number of subjects with adverse events in high dose versus low dose antimony groups. Note: Grey zone area corresponds to area of p-value<0.05; X stands for point estimates; point estimates within the grey zone area have p-values<0.05; circles are sized by the number of patients with adverse events; circles located to the right of the vertical solid line favor more adverse events in the high dose antimony group.

Among other adverse events attributed to antimony, subjects receiving high dose treatment were more likely to present general malaise (75.0% versus 41.7%, p<0.01) and elevated pancreatic enzymes (72.2% versus 36.8%, p = 0.02) than those receiving low dose treatment. Fig 4 presents a graphical display of adverse events using bubble plots to highlight differences in number, variety and distribution between treatments.

More subjects in the high dose group (58.3%) than in the low dose group (19.4%) temporarily discontinued treatment due to AE at any time (p<0.01). Two subjects in the high dose group (drug eruption and arthralgia) and none in the low dose group permanently discontinued treatment due to AE, however both took more than 90% of prescribed doses and evolved to cure without additional treatment. One death occurred in the high dose group within twelve days after antimony-related severe electrocardiographic and laboratory pancreatic abnormalities. Although the cause of death was determined as diabetes mellitus resulting in bacterial sepsis, antimony-related toxicity could not be conclusively ruled out as a contributing factor to the death.

**Fig 5:**
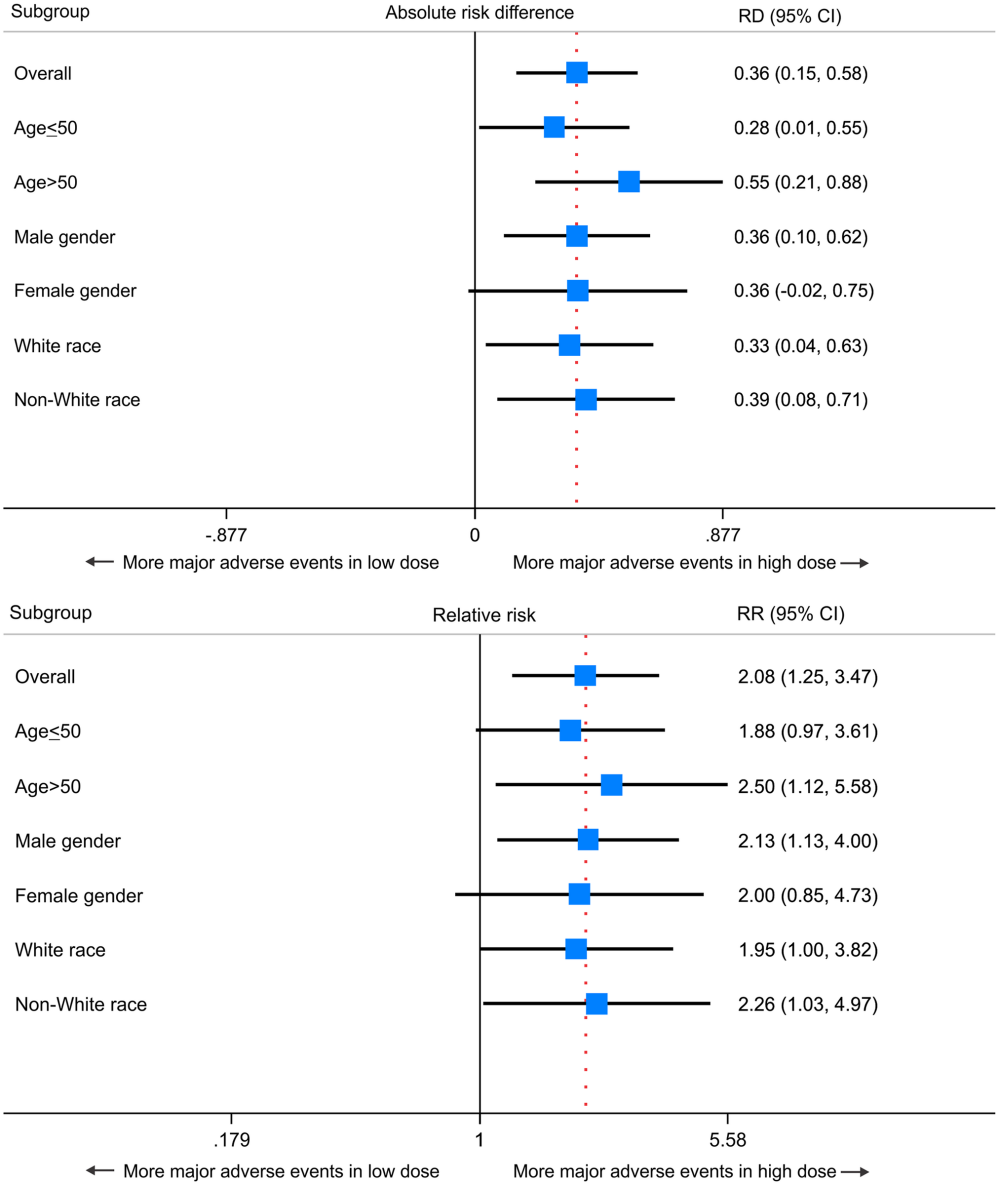
Subgroup analysis: Forest plot of absolute risk difference (RD) and relative risk (RR) with two-sided 95% confidence interval (CI) for major adverse events according to low versus high dose antimony treatment. Squares located to the right of the vertical solid line favor more major adverse events in the high dose antimony group.

Subgroup analysis showed that the risk of major adverse events due to antimony toxic effects were increased in the high dose group regardless of age (subjects aged 50 and younger, as well as older than 50 years’ subgroups), but the magnitude of the risk was greater in subjects older than 50 years of age (Fig 5).

## Discussion

Since the mid-1980s, low dose regimen has been employed at our institution in Rio de Janeiro, Brazil, to treat patients with cutaneous and mucosal leishmaniasis [15–21, 24, 30, 34]. The rationale behind it is intuitively attractive and relies on its expected non-inferior effectiveness, less toxicity, improved compliance and reduced costs [26].

Although previous studies have suggested that low dose regimen is apparently effective, evidence was scarce and based mainly on case reports and observational studies [16, 18–24]. High dose of antimony is the current standard of care for most patients with ACL. Studies, however, have focused mostly on effectiveness and adverse effects [26]. One of the main unanswered questions on this matter at the present is the understanding of the most appropriate dose and time of drug exposure to treat ACL [26]. This question might only be answered with a randomized controlled trial. To our knowledge, this is the first published non-inferiority trial to assess whether low dose regimen is non-inferior to high dose regimen for the treatment of ACL.

In this study, low dose regimen was found to be inferior to standard high dose antimony regimen in terms of clinical cure after one series of treatment for patients with ACL. Conversely, high dose regimen induced a significantly higher toxic effect than the low dose regimen. These results have important clinical relevance and leads to a conundrum: where do you find a fine balance between effectiveness and safety in antimony therapy for ACL?

For decades, antimony compounds have been known to be highly toxic, bearing chemico-toxicological similarities with arsenic [13]. Although the toxic effects of antimony have been known to be almost always reversible, they are frequent; some of them can be severe and lead to death if not promptly identified [35–37]. The high toxicity of antimony was clearly demonstrated in our study, with a large proportion of adverse events in both treatment groups. Our study also found evidence of greater toxicity in the high dose group as compared to the low dose group. There were significant greater numbers of adverse events and major adverse events per subject, more major adverse events and more drug-related discontinuations in the high dose group. The dose dependent toxicity of antimony might account for the differences, and this result is consistent with previous studies [38, 39].

The subgroup analysis of adverse events found that the incidence of major adverse events markedly increased in subjects aged 50 years or older treated at higher doses. A previous study showed that in the treatment of ATL most of adverse events occur in subjects over 50 years of age [40]. While it is an interesting observation, our trial was not powered to detect differences among subgroups and these results serve to suggest that subjects over 50 years old may be a suitable group to assess in further studies.

As the virulence and the intrinsic drug sensitivity differs among species and strains of *Leishmania* [41–43], the management of leishmaniasis should address these differences and also be tailored to provide the optimized balance between efficacy and toxicity [3, 10]. When clinical differences such as comorbidity, immunosuppression and age are added to the balance, markedly different treatment strategies might be required [3, 10].

Currently, leishmaniasis treatment relies exclusively on a limited therapeutic arsenal of highly toxic drugs administered by parenteral route such as pentavalent antimonials, pentamidine, amphotericin B deoxycholate and lipid-based formulations of amphotericin [3, 10]. Although oral miltefosine has recently been shown to be non-inferior to meglumine antimoniate for the treatment of ACL [44, 45], it is not registered nor commercialized in many endemic countries in the world [46]. The past decades have seen a growing interest in the use of alternative treatments for ACL such as thermotherapy, low dose and intralesional antimony, as they could fill the void which currently exists in treating patients with significant less toxicity [3, 47].

Mucosal leishmaniasis (ML) is a rare but dreaded complication of ACL, presenting in less than 5% of the patients after cure [1, 3, 48]. One widely held view is that the benefit of preventing ML should outweigh the toxicity of systemic antileishmanial treatment [49]. Although it is the ultimate aim of any treatment in ACL to prevent mucosal disease, no epidemiological evidence exists to support the assumption that appropriate treatment could decrease the risk of ML [48]. To complicate matter further, in his classical review article, Marsden suggested that even low doses of antimony could prevent mucosal complications [50]. Added impetus to this view is given by a recent study performed at our center (data not yet published) that found that secondary mucosal lesion and relapse rates observed in ACL patients treated with low dose and intralesional antimony were similar to those treated at high doses of antimony at the state of Rio de Janeiro and the Southeast Region of Brazil [51]. In our study, no patient developed mucosal lesion in either arm at a mean follow-up of 3.78 years.

A final intriguing finding of the study is that most subjects who failed initial treatment achieved cure after local therapy or low dose of antimony. We speculate whether this might be explained by an insufficient time of exposure to antimony in our study (low dose treatment might need to be longer) or a natural feature of the disease (a hypothetically different response to locally applied treatment) [52]. In that regard, it is fitting to mention that, although low dose of antimony was found to be inferior to high dose of antimony for clinical cure, our analyses were based on parameters obtained from only the first series of treatment. For the Brazilian Ministry of Health, for example, clinical failure is established after two series of treatment [12]. This is important in light of the fact that our study data suggest that less toxic therapies such as intralesional or low dose of antimony might still be viable and attractive options for treatment, even when a patient has failed initial therapy. Quite interestingly, if a criterium of clinical cure achieved after two series of treatment with the same or less toxic therapies had been used instead in our study, cure rates in both low and high dose groups would be similar.

The choice of the best drug treatment regimen in a disease is a particularly difficult process, in which many factors must be taken into account [53, 54]. Overall, a fine balance between effectiveness and safety depends on the characteristics of the disease being studied [53, 54]. Simply put, effectiveness is the most important parameter for the treatment of a severe or life-threatening disease [53, 54], e.g. visceral leishmaniasis. In this case, the best choice is the maximum tolerated dose [54]. On the other hand, in diseases such as cutaneous leishmaniasis, which rarely evolves to severe impairment, disabling sequelae or death, safety should be paramount and the most appropriate option is the lowest effective dose [53, 54]. In situations where the best dose is not certain, other approaches conducted in different epidemiological contexts, such as cost-effectiveness studies, have emerged as important tools to help identifying the optimal dose and schedule that might work best for each individual [55, 56].

Our trial, although small, holds a very representative sample of patients with cutaneous leishmaniasis, comprising approximately 90% of ATL cases in the metropolitan area of Rio de Janeiro and 50% of the ATL cases in the state of Rio de Janeiro [57]. Our institution is a tertiary referral center for leishmaniasis, but the majority of patients comes spontaneously with a non-diagnosed lesion. Some limitations should nonetheless be kept in mind when interpreting the results. While advanced age per se was not an exclusion criterion for the study, children up to the age of 13 years, patients with signs of severe illness and absolute contraindications to antimony were not eligible to enrollment. These might compromise the external validity of the study. Response to antimonial treatment also may vary according to the infecting species and the geographic site of acquisition [58, 59]. A previous study reported that *Leishmania (Viannia) braziliensis* strains in Rio de Janeiro are highly sensible to meglumine antimoniate in vitro, which could also limit the generalizability of our findings [58].

In summary, our study found that low dose of antimony was inferior to high dose of antimony in terms of clinical cure after one series of treatment of ACL, but was significantly associated with less toxicity. The use of high dose of antimony for the treatment of ACL is supported by a large body of evidence [1, 3, 10, 12, 60] and should remain as the standard treatment. In patients older than 50 years of age or in a subgroup of patients where drug toxicity is an important consideration, low dose might be preferred.

## Supporting information

S1 Appendix Justification of non-inferiority margin and assay sensitivity analysis. (DOCX)

S2 Appendix Flowchart of subjects with diagnosis of clinical failure. (DOCX)

S3 Appendix CONSORT 2010 checklist of information for reporting a randomised trial. (DOCX)

S4 Appendix The CONSORT extension checklist for reporting noninferiority and equivalence trials. (DOC)

S5 Appendix Trial study protocol in English (translated version). (PDF)

S6 Appendix Trial study protocol in Portuguese (original version). (PDF)

## References

[1] Da-Cruz AM, Pirmez C. Leishmaniose tegumentar americana In: Coura JR, editor. Dinâmica das doenças infecciosas e parasitárias. 1 ed Rio de Janeiro: Guanabara Koogan; 2005 p. 697–712.

[2] Pace D. Leishmaniasis. J Infect. 2014;69 Suppl 1:S10–8. 10.1016/j.jinf.2014.07.016 . 25238669

[3] Conceição-Silva F, Alves CRA. Leishmanioses do Continente Americano. Rio de Janeiro: Fiocruz; 2014 521 p.

[4] Alvar J, Yactayo S, Bern C. Leishmaniasis and poverty. Trends Parasitol. 2006;22(12):552–7. 10.1016/j.pt.2006.09.004 . 17023215

[5] WHO Expert Committee on the Control of the Leishmaniases., World Health Organization. Control of the leishmaniases: report of a meeting of the WHO Expert Commitee on the Control of Leishmaniases, Geneva, 22–26 March 2010. Geneva: World Health Organization; 2010. xiii, 186 p.

[6] World Health Organization. Leishmaniasis in high-burden countries: an epidemiological update based on data reported in 2014. Wkly Epidemiol Rec. 2016;91(22):287–96. . 27263128

[7] Rabello E. Les origines de la leishmaniose tégumentaire au Brésil. Congrès des dermatologistes et syphiligraphes de langue française; Juillet 1923; Strasbourg, France;1923.

[8] Rabello E. Formes cliniques de la leishmaniose tégumentaire. Congrès de dermatologistes et syphiligraphes de langue française; Juillet 1923; Strasbourg, France;1923.

[9] Brazil Ministry of Health. Indicadores e dados básicos: taxa de incidência da leishmaniose tegumentar americana 2013 [5 June 2013]. http://dtr2004.saude.gov.br/sinanweb/tabnet/tabnet?sinannet/lta/bases/ltabrnet.def.

[10] World Health Organization. Control of the Leishmaniases: report of a meeting of the WHO Expert Committee. Geneva, Switzerland: World Health Organization; 2010. 186 p.

[11] Vianna G. Tratamento da leishmaniose tegumentar por injeções intravenosas de tártaro emético. Arq Bras Med. 1912;4:426–8.

[12] Brazil Ministry of Health. Manual de Vigilância da Leishmaniose Tegumentar Americana. Brasília, DF: Editora do Ministério da Saúde; 2010 p. 180.

[13] Roberts WL, Berman JD, Rainey PM. In vitro antileishmanial properties of tri- and pentavalent antimonial preparations. Antimicrob Agents Chemother. 1995;39(6):1234–9. ; 7574507 10.1128/aac.39.6.1234 PMC162718

[14] Fontenele e Silva JS, Galvao TF, Pereira MG, Silva MT. Treatment of American tegumentary leishmaniasis in special populations: a summary of evidence. Rev Soc Bras Med Trop. 2013;46(6):669–77. 10.1590/0037-8682-0104-2013 . 24474006

[15] Schubach Ade O, Marzochi KB, Moreira JS, Schubach TM, Araujo ML, Vale AC, et al Retrospective study of 151 patients with cutaneous leishmaniasis treated with meglumine antimoniate. Rev Soc Bras Med Trop. 2005;38(3):213–7. /S0037-86822005000300001. . 15895170 10.1590/s0037-86822005000300001

[16] Oliveira-Neto MP, Schubach A, Mattos M, Goncalves-Costa SC, Pirmez C. Treatment of American cutaneous leishmaniasis: a comparison between low dosage (5 mg/kg/day) and high dosage (20 mg/kg/day) antimony regimens. Pathol Biol (Paris). 1997;45(6):496–9. . 9309267

[17] Oliveira-Neto MP, Schubach A, Mattos M, Goncalves-Costa SC, Pirmez C. A low-dose antimony treatment in 159 patients with American cutaneous leishmaniasis: extensive follow-up studies (up to 10 years). Am J Trop Med Hyg. 1997;57(6):651–5. . 9430521 10.4269/ajtmh.1997.57.651

[18] Oliveira-Neto MP, Schubach A, Mattos M, da Costa SC, Pirmez C. Intralesional therapy of American cutaneous leishmaniasis with pentavalent antimony in Rio de Janeiro, Brazil—an area of Leishmania (V.) braziliensis transmission. Int J Dermatol. 1997;36(6):463–8. . 9248897 10.1046/j.1365-4362.1997.00188.x

[19] Oliveira-Neto MP, Mattos M, Pirmez C, Fernandes O, Goncalves-Costa SC, Souza CF, et al Mucosal leishmaniasis ("espundia") responsive to low dose of N-methyl glucamine (Glucantime) in Rio de Janeiro, Brazil. Rev Inst Med Trop Sao Paulo. 2000;42(6):321–5. . 11136518 10.1590/s0036-46652000000600004

[20] Oliveira Neto MP, Schubach A, Araujo ML, Pirmez C. High and low doses of antimony (Sbv) in American cutaneous leishmaniasis. A five years follow-up study of 15 patients. Mem Inst Oswaldo Cruz. 1996;91(2):207–9. . 8736092 10.1590/s0074-02761996000200016

[21] de Camargo Ferreira EVE, de Oliveira Schubach A, Valete-Rosalino CM, de Souza Coutinho R, Conceicao-Silva F, de Matos Salgueiro M, et al American tegumentary leishmaniasis in older adults: 44 cases treated with an intermittent low-dose antimonial schedule in Rio de Janeiro, Brazil. J Am Geriatr Soc. 2010;58(3):614–6. 10.1111/j.1532-5415.2010.02747.x . 20398135

[22] Costa JM, Marsden PD. Low dose glucantime therapy in Leishmania viannia braziliensis (Lvb) infections. Rev Soc Bras Med Trop. 1988;21(2):85–6. . 3249826 10.1590/s0037-86821988000200013

[23] Amato VS, de Oliveira LS, Silva AC, Machado FR, Amato JG, Nicodemo AC, et al [A case of mucocutaneous leishmaniasis treated with success with a low dose of pentavalent antimonial]. Rev Soc Bras Med Trop. 1998;31(2):221–4. . 9608241 10.1590/s0037-86821998000200008

[24] de Oliveira-Neto MP, Mattos Mda S. Successful therapeutic response of resistant cases of mucocutaneous leishmaniasis to a very low dose of antimony. Rev Soc Bras Med Trop. 2006;39(4):376–8. . 17119754 10.1590/s0037-86822006000400011

[25] Olliaro P, Vaillant M, Arana B, Grogl M, Modabber F, Magill A, et al Methodology of clinical trials aimed at assessing interventions for cutaneous leishmaniasis. PLoS Negl Trop Dis. 2013;7(3):e2130 10.1371/journal.pntd.0002130 ; 23556016 PMC3605149

[26] Ampuero JS. Efficacy and safety of low-dose pentavalent antimonial for treatment of cutaneous leishmaniasis by Leishmania (Viannia) braziliensis in Bahia, Brazil: a randomized clinical trial [Doctoral thesis]. Brasília, DF: Universidade de Brasília; 2009.

[27] Division of AIDS, National Institute of Allergy Infectious Diseases, National Institutes of Health. Division of AIDS table for grading the severity of adult and pediatric adverse events 2004 [12 May 2015]. http://rsc.tech-res.com/Document/safetyandpharmacovigilance/Table_for_Grading_Severity_of_Adult_Pediatric_Adverse_Events.pdf.

[28] Navin TR, Arana BA, Arana FE, Berman JD, Chajon JF. Placebo-controlled clinical trial of sodium stibogluconate (Pentostam) versus ketoconazole for treating cutaneous leishmaniasis in Guatemala. J Infect Dis. 1992;165(3):528–34. . 1311351 10.1093/infdis/165.3.528

[29] Navin TR, Arana BA, Arana FE, de Merida AM, Castillo AL, Pozuelos JL. Placebo-controlled clinical trial of meglumine antimonate (glucantime) vs. localized controlled heat in the treatment of cutaneous leishmaniasis in Guatemala. Am J Trop Med Hyg. 1990;42(1):43–50. . 2405727 10.4269/ajtmh.1990.42.43

[30] Mattos MdS. Determinação de parâmetros clínicos e prognósticos para o controle de cura da Leishmaniose Tegumentar Americana [Doctoral thesis]. Rio de Janeiro, RJ: Fundação Oswaldo Cruz; 2004.

[31] Food and Drug Administration, U.S. Department of Health and Human Services Guidance for Industry: non-Inferiority clinical trials. Rockville, MD: Food and Drug Administration; 2010 63 p.

[32] Nelson W. Applied life data analysis. New York: Wiley; 1982 xiv, 634 p.

[33] Cupolillo E, Grimaldi G Jr., Momen H. A general classification of New World Leishmania using numerical zymotaxonomy. Am J Trop Med Hyg. 1994;50(3):296–311. . 8147488 10.4269/ajtmh.1994.50.296

[34] Antonio LdF. Resposta à Intradermorreação de Montenegro e ocorrência de falha terapêutica na forma cutânea da leishmaniose tegumentar americana: um estudo de caso controle [Master's dissertation]. Rio de Janeiro, RJ: Fundação Oswaldo Cruz; 2012.

[35] Oliveira AL, Brustoloni YM, Fernandes TD, Dorval ME, Cunha RV, Boia MN. Severe adverse reactions to meglumine antimoniate in the treatment of visceral leishmaniasis: a report of 13 cases in the southwestern region of Brazil. Trop Doct. 2009;39(3):180–2. 10.1258/td.2008.080369 . 19535762

[36] Oliveira LF, Schubach AO, Martins MM, Passos SL, Oliveira RV, Marzochi MC, et al Systematic review of the adverse effects of cutaneous leishmaniasis treatment in the New World. Acta Trop. 2011;118(2):87–96. 10.1016/j.actatropica.2011.02.007 . 21420925

[37] Sundar S, Chakravarty J. Antimony toxicity. Int J Environ Res Public Health. 2010;7(12):4267–77. 10.3390/ijerph7124267 ; 21318007 PMC3037053

[38] Chulay JD, Spencer HC, Mugambi M. Electrocardiographic changes during treatment of leishmaniasis with pentavalent antimony (sodium stibogluconate). Am J Trop Med Hyg. 1985;34(4):702–9. . 2992303 10.4269/ajtmh.1985.34.702

[39] Neves DB, Caldas ED, Sampaio RN. Antimony in plasma and skin of patients with cutaneous leishmaniasis—relationship with side effects after treatment with meglumine antimoniate. Trop Med Int Health. 2009;14(12):1515–22. 10.1111/j.1365-3156.2009.02408.x . 19954451

[40] Diniz DS, Costa AS, Escalda PM. The effect of age on the frequency of adverse reactions caused by antimony in the treatment of American tegumentary leishmaniasis in Governador Valadares, State of Minas Gerais, Brazil. Rev Soc Bras Med Trop. 2012;45(5):597–600. . 23152343 10.1590/s0037-86822012000500011

[41] Martinez JE, Travi BL, Valencia AZ, Saravia NG. Metastatic capability of Leishmania (Viannia) panamensis and Leishmania (Viannia) guyanensis in golden hamsters. J Parasitol. 1991;77(5):762–8. . 1919926

[42] Mohapatra S. Drug resistance in leishmaniasis: Newer developments. Trop Parasitol. 2014;4(1):4–9. 10.4103/2229-5070.129142 ; 24754020 PMC3992802

[43] Grimaldi G Jr., Porrozzi R, Friedrich K, Teva A, Marchevsky RS, Vieira F, et al Comparative efficacies of two antimony regimens to treat Leishmania braziliensis-induced cutaneous Leishmaniasis in rhesus macaques (Macaca mulatta). Antimicrob Agents Chemother. 2010;54(1):502–5. 10.1128/AAC.00858-09 ; 19822700 PMC2798537

[44] Chrusciak-Talhari A, Dietze R, Chrusciak Talhari C, da Silva RM, Gadelha Yamashita EP, de Oliveira Penna G, et al Randomized controlled clinical trial to access efficacy and safety of miltefosine in the treatment of cutaneous leishmaniasis Caused by Leishmania (Viannia) guyanensis in Manaus, Brazil. Am J Trop Med Hyg. 2011;84(2):255–60. 10.4269/ajtmh.2011.10-0155 ; 21292895 PMC3029178

[45] Machado PR, Ampuero J, Guimaraes LH, Villasboas L, Rocha AT, Schriefer A, et al Miltefosine in the treatment of cutaneous leishmaniasis caused by Leishmania braziliensis in Brazil: a randomized and controlled trial. PLoS Negl Trop Dis. 2010;4(12):e912 10.1371/journal.pntd.0000912 ; 21200420 PMC3006132

[46] Drugs for Neglected Diseases initiative (DNDi). Leishmaniasis factsheet 2015 [cited 2016 November 6, 2016]. http://www.dndi.org/wp-content/uploads/2016/10/Factsheet_2015_Leish.pdf.

[47] Cardona-Arias JA, Velez ID, Lopez-Carvajal L. Efficacy of thermotherapy to treat cutaneous leishmaniasis: a meta-analysis of controlled clinical trials. PLoS One. 2015;10(5):e0122569 10.1371/journal.pone.0122569 ; 26009885 PMC4444239

[48] Herwaldt BL. Leishmaniasis. Lancet. 1999;354(9185):1191–9. 10.1016/S0140-6736(98)10178-2 . 10513726

[49] Blum J, Lockwood DN, Visser L, Harms G, Bailey MS, Caumes E, et al Local or systemic treatment for New World cutaneous leishmaniasis? Re-evaluating the evidence for the risk of mucosal leishmaniasis. Int Health. 2012;4(3):153–63. 10.1016/j.inhe.2012.06.004 . 24029394

[50] Marsden PD. Mucosal leishmaniasis ("espundia" Escomel, 1911). Trans R Soc Trop Med Hyg. 1986;80(6):859–76. . 3037735 10.1016/0035-9203(86)90243-9

[51] Brahim-Paes LRdN. Distribuição espaçotemporal dos casos humanos de leishmaniose tegumentar americana notificados no estado do Rio de Janeiro de 2001 a 2013 e associação com variáveis clínicas e populacionais [Doctoral thesis]. Rio de Janeiro, RJ: Fundação Oswaldo Cruz; 2016.

[52] Lakhal-Naouar I, Slike BM, Aronson NE, Marovich MA. The Immunology of a Healing Response in Cutaneous Leishmaniasis Treated with Localized Heat or Systemic Antimonial Therapy. PLoS Negl Trop Dis. 2015;9(10):e0004178 10.1371/journal.pntd.0004178 ; 26485398 PMC4618688

[53] McCormack JP, Allan GM, Virani AS. Is bigger better? An argument for very low starting doses. CMAJ. 2011;183(1):65–9. 10.1503/cmaj.091481 ; 20921252 PMC3017255

[54] Chin R, Lee BY. Dosing and intervention Principles and Practice of Clinical Trial Medicine. 1 ed Amsterdam: Elsevier Academic Press; 1996 p. 181–212.

[55] Neumann PJ. Using cost-effectiveness analysis to improve health care: opportunities and barriers. Oxford; New York: Oxford University Press; 2005 xii, 209 p. p.

[56] World Health Organization., Baltussen RMPM, Adam T, Tan-Torres Edejer T, Hutubessy RCW, Acharya A, et al Making choices in health: WHO guide to cost-effectiveness analysis. Geneva: World Health Organization; 2003 318 p. + 1 CD-ROM. p.

[57] Rio de Janeiro Municipal Secretary of Health Leishmaniose tegumentar americana: investigação SINAN-NET 10.05.2012. Rio de Janeiro, RJ: Sanitary Dermatology Management; 2012.

[58] Azeredo-Coutinho RB, Mendonca SC, Callahan H, Portal AC, Max G. Sensitivity of Leishmania braziliensis promastigotes to meglumine antimoniate (glucantime) is higher than that of other Leishmania species and correlates with response to therapy in American tegumentary leishmaniasis. J Parasitol. 2007;93(3):688–93. 10.1645/GE-1031R.1 . 17626365

[59] Yardley V, Ortuno N, Llanos-Cuentas A, Chappuis F, Doncker SD, Ramirez L, et al American tegumentary leishmaniasis: Is antimonial treatment outcome related to parasite drug susceptibility? J Infect Dis. 2006;194(8):1168–75. 10.1086/507710 . 16991093

[60] Gonzalez U, Pinart M, Rengifo-Pardo M, Macaya A, Alvar J, Tweed JA. Interventions for American cutaneous and mucocutaneous leishmaniasis. Cochrane Database Syst Rev. 2009;(2):CD004834 10.1002/14651858.CD004834.pub2 . 19370612

