# Supplementary material for: Low versus high dose of antimony for American cutaneous leishmaniasis: A randomized controlled blind non-inferiority trial in Rio de Janeiro, Brazil
Source: PLoS One. 2017 May 30;12(5):e0178592. doi: 10.1371/journal.pone.0178592 (PMC5448803; doi:10.1371/journal.pone.0178592)
Supplement: S1 Appendix — (DOCX) [file pone.0178592.s001.docx]

## **S1 Appendix.** Justification of non-inferiority margin and assay sensitivity analysis

Our review of historical data of treatment of American cutaneous leishmaniasis (ACL) with antimony in Rio Janeiro showed that clinical cure rates ranged from 81.8 to 100.0% [1-9]. However, these findings were difficult to analyze and compare because of significant differences in recording techniques, endpoint definitions, follow-up times and antimony dose regimens. After a discussion panel between clinical investigators and statisticians, the judgement was to consider historical information from our cohort (95% cure rate) [10] and extract information from the largest studies with endpoints evaluations at 12+ months (93.2% to 100% cure rate) [1, 6]. To find failure rates without treatment, we searched placebo controlled studies in ACL caused by *Leishmania (Viannia) braziliensis* on relevant key words in a range of research databases. We found four studies which showed that spontaneous cure in ACL caused by *Leishmania braziliensis* was a rare event and occurred in only 0.0 to 9.1% of patients [11-14].

In this study, we assumed that high dose antimony treatment would be 95% effective and failure in placebo was assumed to be 90%. As the first step, to determine M1 (largest acceptable margin), which corresponds to the effect of high dose antimony treatment over placebo, we applied the formula to find expected failure rate in control: (1.0 – (High dose antimony effectiveness)) x (Failure rate on placebo). The expected failure rate in high dose group would be: (1.0 – 0.95) x (90%) = 4.5%. The formula to find M1 would be: (Failure rate in placebo – Failure rate in high dose) = (90.0 – 4.5) = 85.5%. On step two, to find the expected failure of low dose treatment over placebo, as non-inferiority margin (M2) was defined beforehand, we applied the formula: (M2 + Failure rate in high dose) = (15.0 + 4.5) = 19.5%. Expected improvement of low dose treatment over placebo would be: (Failure rate in placebo – Failure rate in low dose) = (90.0 – 19.5) = 70.5%. To find the M1 margin preservation rate, we applied the formula: (Expected improvement of low dose over placebo / M1) = (70.5 / 85.5) = 82.5%. In other words, applying a non-inferiority margin of 15%, low dose treatment preserves at least 82.5% of the effect of high dose antimony treatment compared to placebo.

Assay sensitivity analysis creating a simulation of different scenarios is shown in the table below. Our findings suggest that, even in the worst possible scenario, a significant fraction of M1 (75.0%) would still be preserved.

| **High dose antimony effectiveness rate (%)** | **Failure rate on placebo (%)** | **Expected failure rate in high dose (%)** | **M1 (%)** | **M2 (%)** | **Expected failure rate in low dose (%)** | **Improvement of low dose treatment over placebo (%)** | **M1 preservation (%)** |
| --- | --- | --- | --- | --- | --- | --- | --- |
| 95.0 | 95.0 | 4.8 | 90.3 | 15.0 | 19.8 | 75.2 | 83.4 |
| 95.0 | 90.0 | 4.5 | 85.5 | 15.0 | 19.5 | 70.5 | 82.5 |
| 95.0 | 85.0 | 4.3 | 80.8 | 15.0 | 19.3 | 65.8 | 81.4 |
| 95.0 | 80.0 | 4.0 | 76.0 | 15.0 | 19.0 | 61.0 | 80.3 |
| 95.0 | 75.0 | 3.8 | 71.3 | 15.0 | 18.8 | 56.3 | 78.9 |
| 90.0 | 95.0 | 9.5 | 85.5 | 15.0 | 24.5 | 70.5 | 82.5 |
| 90.0 | 90.0 | 9.0 | 81.0 | 15.0 | 24.0 | 66.0 | 81.5 |
| 90.0 | 85.0 | 8.5 | 76.5 | 15.0 | 23.5 | 61.5 | 80.4 |
| 90.0 | 80.0 | 8.0 | 72.0 | 15.0 | 23.0 | 57.0 | 79.2 |
| 90.0 | 75.0 | 7.5 | 67.5 | 15.0 | 22.5 | 52.5 | 77.8 |
| 85.0 | 95.0 | 14.3 | 80.8 | 15.0 | 29.3 | 65.8 | 81.4 |
| 85.0 | 90.0 | 13.5 | 76.5 | 15.0 | 28.5 | 61.5 | 80.4 |
| 85.0 | 85.0 | 12.8 | 72.3 | 15.0 | 27.8 | 57.3 | 79.2 |
| 85.0 | 80.0 | 12.0 | 68.0 | 15.0 | 27.0 | 53.0 | 77.9 |
| 85.0 | 75.0 | 11.3 | 63.8 | 15.0 | 26.3 | 48.8 | 76.5 |
| 80.0 | 95.0 | 19.0 | 76.0 | 15.0 | 34.0 | 61.0 | 80.3 |
| 80.0 | 90.0 | 18.0 | 72.0 | 15.0 | 33.0 | 57.0 | 79.2 |
| 80.0 | 85.0 | 17.0 | 68.0 | 15.0 | 32.0 | 53.0 | 77.9 |
| 80.0 | 80.0 | 16.0 | 64.0 | 15.0 | 31.0 | 49.0 | 76.6 |
| 80.0 | 75.0 | 15.0 | 60.0 | 15.0 | 30.0 | 45.0 | 75.0 |

Note: M1 stands for largest acceptable margin, M2 stands for non-inferiority margin

## References

1. Schubach Ade O, Marzochi KB, Moreira JS, Schubach TM, Araujo ML, Vale AC, et al. Retrospective study of 151 patients with cutaneous leishmaniasis treated with meglumine antimoniate. Rev Soc Bras Med Trop. 2005;38(3):213-7. doi: /S0037-86822005000300001. PubMed PMID: 15895170.

2. Oliveira-Neto MP, Schubach A, Mattos M, Goncalves-Costa SC, Pirmez C. Treatment of American cutaneous leishmaniasis: a comparison between low dosage (5 mg/kg/day) and high dosage (20 mg/kg/day) antimony regimens. Pathol Biol (Paris). 1997;45(6):496-9. PubMed PMID: 9309267.

3. Oliveira-Neto MP, Schubach A, Mattos M, Goncalves-Costa SC, Pirmez C. A low-dose antimony treatment in 159 patients with American cutaneous leishmaniasis: extensive follow-up studies (up to 10 years). Am J Trop Med Hyg. 1997;57(6):651-5. PubMed PMID: 9430521.

4. Oliveira-Neto MP, Schubach A, Mattos M, da Costa SC, Pirmez C. Intralesional therapy of American cutaneous leishmaniasis with pentavalent antimony in Rio de Janeiro, Brazil--an area of Leishmania (V.) braziliensis transmission. Int J Dermatol. 1997;36(6):463-8. PubMed PMID: 9248897.

5. Oliveira Neto MP, Schubach A, Araujo ML, Pirmez C. High and low doses of antimony (Sbv) in American cutaneous leishmaniasis. A five years follow-up study of 15 patients. Mem Inst Oswaldo Cruz. 1996;91(2):207-9. PubMed PMID: 8736092.

6. Mattos MdS. Determinação de parâmetros clínicos e prognósticos para o controle de cura da Leishmaniose Tegumentar Americana [Doctoral thesis]. Rio de Janeiro, RJ: Fundação Oswaldo Cruz; 2004.

7. de Oliveira-Neto MP, Mattos Mda S. Successful therapeutic response of resistant cases of mucocutaneous leishmaniasis to a very low dose of antimony. Rev Soc Bras Med Trop. 2006;39(4):376-8. PubMed PMID: 17119754.

8. de Camargo Ferreira EVE, de Oliveira Schubach A, Valete-Rosalino CM, de Souza Coutinho R, Conceicao-Silva F, de Matos Salgueiro M, et al. American tegumentary leishmaniasis in older adults: 44 cases treated with an intermittent low-dose antimonial schedule in Rio de Janeiro, Brazil. J Am Geriatr Soc. 2010;58(3):614-6. doi: 10.1111/j.1532-5415.2010.02747.x. PubMed PMID: 20398135.

9. Antonio LdF. Resposta à Intradermorreação de Montenegro e ocorrência de falha terapêutica na forma cutânea da leishmaniose tegumentar americana: um estudo de caso controle [Master's dissertation]. Rio de Janeiro, RJ: Fundação Oswaldo Cruz; 2012.

10. Conceição-Silva F, Alves CRA. Leishmanioses do Continente Americano. Rio de Janeiro: Fiocruz; 2014. 521 p.

11. Navin TR, Arana BA, Arana FE, de Merida AM, Castillo AL, Pozuelos JL. Placebo-controlled clinical trial of meglumine antimonate (glucantime) vs. localized controlled heat in the treatment of cutaneous leishmaniasis in Guatemala. Am J Trop Med Hyg. 1990;42(1):43-50. PubMed PMID: 2405727.

12. Navin TR, Arana BA, Arana FE, Berman JD, Chajon JF. Placebo-controlled clinical trial of sodium stibogluconate (Pentostam) versus ketoconazole for treating cutaneous leishmaniasis in Guatemala. J Infect Dis. 1992;165(3):528-34. PubMed PMID: 1311351.

13. Herwaldt BL, Arana BA, Navin TR. The natural history of cutaneous leishmaniasis in Guatemala. J Infect Dis. 1992;165(3):518-27. PubMed PMID: 1538157.

14. Soto J, Arana BA, Toledo J, Rizzo N, Vega JC, Diaz A, et al. Miltefosine for new world cutaneous leishmaniasis. Clin Infect Dis. 2004;38(9):1266-72. doi: 10.1086/383321. PubMed PMID: 15127339.
