# Supplementary material for: Low versus high dose of antimony for American cutaneous leishmaniasis: A randomized controlled blind non-inferiority trial in Rio de Janeiro, Brazil
Source: PLoS One. 2017 May 30;12(5):e0178592. doi: 10.1371/journal.pone.0178592 (PMC5448803; doi:10.1371/journal.pone.0178592)
Supplement: S2 Appendix — (DOCX) [file pone.0178592.s002.docx]

## **S2 Appendix.** Flowchart of subjects with diagnosis of clinical failure


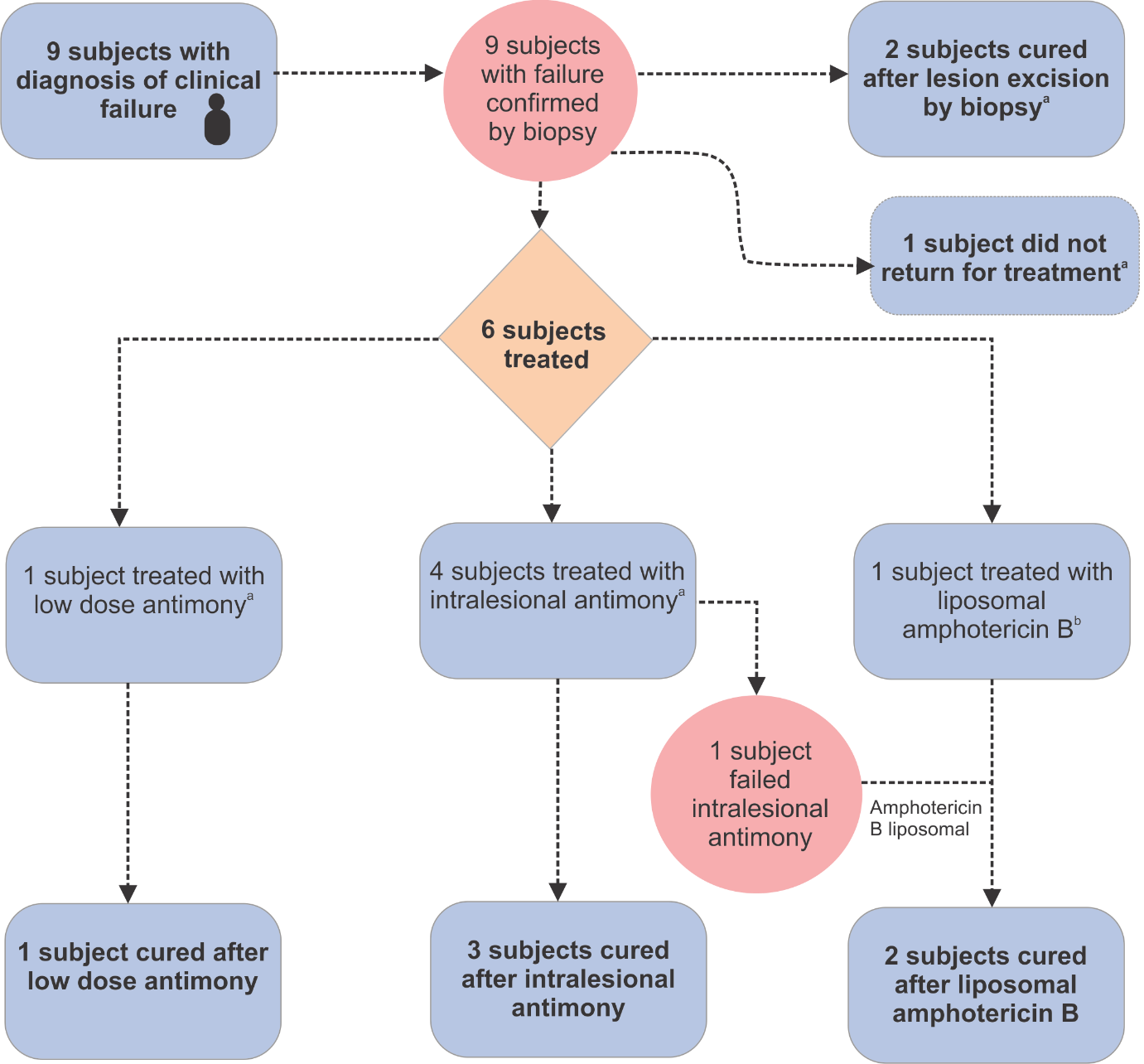


^a^ Subjects in this category had received prior first treatment with low dose antimony

^b^ Subjects in this category had received prior first treatment with high dose antimony
