# Supplementary material for: Low versus high dose of antimony for American cutaneous leishmaniasis: A randomized controlled blind non-inferiority trial in Rio de Janeiro, Brazil
Source: PLoS One. 2017 May 30;12(5):e0178592. doi: 10.1371/journal.pone.0178592 (PMC5448803; doi:10.1371/journal.pone.0178592)
Supplement: S5 Appendix — (PDF) [file pone.0178592.s005.pdf]

PHASE III CLINICAL TRIAL FOR AMERICAN TEGUMENTARY LEISHMANIASIS. EQUIVALENCE BETWEEN THE STANDARD AND ALTERNATIVE SCHEME WITH MEGLUMINE ANTIMONIATE.

Subprojects

A. Controlled, randomized, double-blind, and phase III clinical trial to verify the equivalence of effectiveness and compare the safety between standard and alternative dose regimens of meglumine antimoniate in the treatment of cutaneous leishmaniasis.

B. Development and application of methodologies for analysis of antimony speciation in patients with leishmaniasis treated with meglumine antimoniate.

C. Comparison of the anti-*Leishmania* immune response in patients with American cutaneous leishmaniasis treated with standard or alternative dose of meglumine antimoniate.

D. Comparison of *in vitro* cellular immune response to reference and parasite antigens isolated from the respective American tegumentary leishmaniasis patients who evolved to cure or reactivate the lesions after antimonial therapy

E. Evaluation of genetic variability and *in vitro* antimonial sensitivity of *Leishmania (V.) braziliensis* samples isolated from patients before and after treatment with standard or alternative regimen of meglumine antimoniate.

F. Blind study to evaluate the effectiveness and safety of intralesional meglumine antimoniate in patients with cutaneous leishmaniasis and contraindication to systemic therapy.

G. Blind study to evaluate the effectiveness and safety of intralesional meglumine antimoniate in the treatment of patients with cutaneous leishmaniasis excluded from subproject A (systemic treatment with meglumine antimoniate).

H. Phase III clinical trial for mucocutaneous or mucosal leishmaniasis. Comparison between the standard and alternative scheme with meglumine antimoniate.

I. Blind study to evaluate the effectiveness and safety of intermittent low dose meglumine antimoniate in the treatment of patients with mucosal leishmaniasis excluded from the H subproject (standard or continuous low dose regimen).

J. Evaluation of patients’ adherence in the Phase III clinical trial with standard and alternative scheme with meglumine antimoniate in the treatment of American cutaneous leishmaniasis.

K. Clinical-molecular study on mucosal leishmaniasis: diagnosis and screening of subpopulations of *Leishmania (Viannia) braziliensis*.

Participating Institutions

OSWALDO CRUZ FOUNDATION (FIOCRUZ)

Av. Brasil, 4365- Manguinhos, Rio de Janeiro

CEP: 21.040-900 Tel: (xx 21) 3865-8235

Evandro Chagas Clinical Research Institute (IPEC)

Av. Brasil, 4365 Manguinhos, Rio de Janeiro

CEP: 21040-900 Tel: (21) 3865-9595 Fax: (21) 3865-9541

1. Infectious Diseases Service

2. Reference Center for Leishmaniasis

3. Department of Epidemiology

4. Zoonoses Service

5. Pathology Service

6. Parasitology Service

7. Clinical Specialties Service

8. Pharmacokinetic service

9. Pharmacy Service

10. Clinical Pathology Service

Oswaldo Cruz Institute (IOC)

Av. Brasil, 4365 Manguinhos, Rio de Janeiro

CEP: 21040-900 Tel: (21) 3865-9595 Fax: (21) 3865-9541

11. Immunoparasitology Laboratory

12. Biochemistry Laboratory of Trypanosomatids

13. Biochemical Systematics Laboratory

PONTIFICAL CATHOLIC UNIVERSITY OF RIO DE JANEIRO (PUC)

Av. Brasil, 4365 Manguinhos, Rio de Janeiro

CEP: 21040-900 Tel: (21) 3865-9595 Fax: (21) 3865-9541

14. Department of Chemistry

SUBPROJECT A

A controlled, randomized, double-blind, and phase III clinical trial to verify the equivalence of effectiveness and compare the safety between standard and alternative dose regimens of meglumine antimoniate in the treatment of cutaneous leishmaniasis

A 5. Hypotheses to be tested

A 5.1 Non-inferiority of effectiveness

H_{0E} = There is no non-inferiority between the regimen currently recommended in Brazil for the treatment of cutaneous leishmaniasis (20 mg Sb5 + / kg / day for 20 days) and the alternative regimen with 5 mg for 30 days, i.e. the alternative scheme is not non-inferior to the standard regimen.

H_{1E} = the alternative 5 mg schedule is non-inferior to the currently recommended 20 mg antimony regimen.

A 5.2 Safety

H_{0S} = There is an equivalence of toxicity between the currently recommended regimen in Brazil for the treatment of cutaneous leishmaniasis (20mg Sb5 + / kg / day for 20) and the alternative regimen with 5mg for 30 days, i.e. no difference in toxicity between the schemes.

H_{1S} = the currently recommended 20 mg regimen is more toxic than the alternative regimen.

Regardless of the therapeutic regimen, lesions located above the knees are expected to be epithelialized at the end of treatment, while epithelialization of localized lesions in the legs and feet occurs more frequently after the end of the treatment period. Administration of the drugs, particularly in those cases associated with vascular insufficiency. It is also expected that, regardless of the therapeutic regimen, patients over 50 years old present adverse effects more frequently and more intensely than younger patients.

A 6. Objectives

A 6.1 General objective

To compare the efficacy and safety of meglumine antimoniate at a dose of 20 mg Sb^{5+}/ kg/day for 20 days or at 5 mg for 30 days in the treatment of patients with cutaneous leishmaniasis.

A 6.2 Specific objectives

1. To compare the immediate effectiveness (initial cure) and effectiveness after one year of follow-up (definitive cure) between the standard regimen recommended by the Brazilian Ministry of Health and the alternative regimen for the treatment of cutaneous leishmaniasis, with a non-inferiority margin of 15%.

2. To compare the frequency and severity of clinical, laboratory and electrocardiographic adverse effects among the different antimonial treatment groups.

3. Compare the frequency and severity of adverse effects and effectiveness among groups according to age, gender and race.

4. Compare the frequencies of epithelialization achieved on days 20, 30 and 50 of treatment among patient groups.

5. Compare the time in days until the epithelialization of the lesions according to the location above and below the knees, between the standard and alternative regimen of antimonial treatment, controlling for the concomitance with associated vascular insufficiency.

A 7. Subjects and methods

A 7.1 Study outline

Randomized, controlled to the standard treatment, double-blind and phase III clinical trial.

A 7.2 Description of the medication and schedule of interventions

In Brazil, meglumine antimoniate (Aventis, São Paulo, Brazil) is stored at room temperature and distributed to the health network by the Health Surveillance Secretariat

- SVS / Brazilian Ministry of Health, which will provide a single lot to be used in all patients of the study.

This drug is for intramuscular (deltoid or gluteal) application in a single daily dose. Sterile plastic disposable syringes of 5 to 20 mL and disposable, sterile 25 × 7 mm needles will be employed.

The study using the intramuscular (IM) route without direct supervision will allow the evaluation of its use in real-world conditions (effectiveness) employed by the primary health services in the state of Rio de Janeiro. Although intravenous prescription is possible, it is not a usual route of antimony administration and it is also difficult to implement in an outpatient primary setting.

Each patient will be included in one of the following meglumine antimoniate treatment groups per IM route:

1. 20mg Sb5 + / kg / day for 20 days

2. 5mg Sb5 + / kg / day for 30 days

There will be no cross-over between the groups for the purposes of this study. The data of those patients who need a definitive interruption of a regimen will be analyzed according to the group to which they were randomized, which means, by intention to treat. Data collection will take place according to the consultation schedule.

A 7.3 Sampling plan

A 7.3.1 Sample size

The groups with the minimum sample size (36 patients) are expected to respond to all the outcomes of interest.

We believe that the comparison of effectiveness between the two schemes will reveal non-inferior results to the alternative regimen for the following outcomes:

1. frequency of good initial response (evaluated on days 20, 30 and 50).

2. time (in days) until epithelization of all lesions is reached.

3. time (in days) to achieve total healing of all lesions.

4. frequency of good late response (one year of outpatient follow-up according to study schedule).

6. frequency of reactivation after treatment (up to two years of outpatient follow-up according to study schedule).

The significance level of 5% and power of 80% were used to calculate the required sample size to compare the frequencies of the outcomes of interest in the standard group with the other group. To test the non-inferiority between proportions in the primary endpoints of effectiveness, an acceptable limit of 15% difference between these healing ratios was considered. This will require 36 patients in each therapeutic regimen group.

Predicting the use of the McNemar paired test to compare the rates of healing or epithelialization on days 20, 30 and 50 will require at least 36 patients in each group (assuming a medically irrelevant difference of 30%).

To compare the effectiveness of treatment according to the location of the lesions, above or below the knee, only 12 patients will be needed in each treatment group.

It is expected that the alternative group (low dose, 5 mg Sb^{5+}/kg/day) will present non-inferior results with a margin of 15%, and a lower frequency of clinical adverse effects, in addition to a low frequency of laboratory and electrocardiographic adverse effects. However, we expect to find a significant difference in the following outcomes when comparing the safety of the standard 20mg treatment with the alternative regimen:

1. frequency of clinical, laboratory or electrocardiographic adverse effects in any degree of intensity.

2. frequency of clinical, laboratorial or electrocardiographic effects of greater intensity.

3. frequency of treatment interruption caused by Adverse Events.

4. frequency of treatment dropouts.

In the case of elderly patients, a marked increase in the frequency and severity of AE in group 1 (20mg) is expected when compared to group 2 (5mg).

The significance level of 5% and the power of 80% were used to calculate the sample size needed to compare the standard group with the other group.

A 7.3.2 Allocation strategy (randomization)

Eligible individuals (see eligibility criteria) who agree to participate (signing the informed consent form) will be allocated randomly in one of the treatment groups, according to the order of arrival, until the groups are completed. The randomized (numbered) allocation

list will be constructed in EPI-INFO 6.4 with the total number of subjects required for the survey and made available at the IPEC pharmacy. There will be stratification with interaction analysis of clinical and epidemiological aspects that may have a modifier effect on the endpoints (age, gender and race) and blocking with pre-defined blocks of twelve in order to ensure the balance between treatment groups at any time in case of need to stop the research.

A 7.4 Eligibility criteria

A 7.4.1 Inclusion criteria

1. cutaneous leishmaniasis (CL) with parasitological diagnosis by one or more of the following methods: direct examination (scraping or imprint), histopathology, culture, immunohistochemistry or PCR.

2. history of exposure in the endemic area of the state of Rio de Janeiro.

3. absence of previous treatment with meglumine antimoniate.

A 7.4.2 Exclusion Criteria

1. women who do not use contraceptive methods or do so improperly.

2. pregnant women.

3. children under 13.

4. previous treatment with meglumine antimoniate.

5. use of immunosuppressive therapy (corticosteroid, chemotherapy for cancer) or use. of medications for tuberculosis or leprosy.

6. presence of clinical baseline changes equivalent to adverse effect level> G3.

7. presence of laboratory abnormalities equivalent to adverse effect level> G2.

8. presence of baseline electrocardiographic changes equivalent to adverse effect> G4 and / or baseline QTc > 0.46ms (equivalent to G1 level A).

A 7.5 Study patients and schedule for inclusion

The study will include 72 patients with CL coming from the State of Rio de Janeiro, attended at the Reference Center on Leishmaniasis - IPEC - Fiocruz.

As lesions located below the knee are expected to only be fully epithelized several weeks after the end of treatment, bias may occur in the assessment of treatment effectiveness if patients with this lesion location were more frequently present in a given treatment group. Likewise, patients older than 50 years usually present adverse effects more frequently and with greater intensity, and may negatively influence the safety assessment if they were present more frequently in a given group. These inclusion biases will be avoided through the strategy of block randomization and controlled with multivariate analysis, considering the following variables as potential confounders: age above and below 50 years and presence of lesions above and below the knees.

A 7.6 Outcomes

A 7.6.1 Outcomes of Effectiveness: Definition

1. Initial therapeutic response - presence or absence of total epithelization of all lesions until the consultation on day 120 (initial cure).

2. Late therapeutic response - presence or absence of the following elements in the progression expected for total healing:

- disappearance of crusts until the consultation of day 140;
- disappearance of desquamation (smooth surface) until the consultation of the day 230;
- disappearance of infiltration until the consultation of day 320;
- disappearance of erythema until the consultation on day 360 (definitive cure);
- no appearance of mucosal lesion until the consultation of day 770;
- reappearance of any stage previous to that achieved, maintained in 2 observations performed with an interval of at least two weeks.

A 7.6.2 Safety outcomes (adverse events): definition, strength and relationship to study drug

An adverse event (AE) is any unanticipated or unfavorable event that either the investigator or the patient reports, starting during the use of the medication or within 30 days after its suspension. The AE examination will be made by spontaneous remembrance and questioned by the physician according to a standardized form on days 10, 20, 30, 50, 60 and 80.

The classification of the severity of adverse events (clinical, laboratory and electrocardiographic) will be given according to the tables in annexes 1 and 2 adapted from the "AIDS Table for Grading Severity of Adult Adverse Experiences, 1992" (Adult AIDS Clinical Trials Group August 1992).

The causal relationship with the study drug (= adverse effect) will be evaluated by the investigator and classified as follows:

1. Definitive (Highly Likely): A reaction that occurs within a reasonable time sequence after drug administration or when drug levels have established in body fluids and tissues; which follows a known standard response of the suspected drug; which is confirmed by the improvement after stopping the drug and reappears on repeated exposure.

2. Likely: A reaction that occurs within a reasonable time sequence after administration of the drug; which follows a known standard response of the suspected drug; which is confirmed by the improvement after stopping of the drug and which cannot be reasonably explained by the known characteristics of the individual's clinical condition.

3. Possible: A reaction that occurs within a reasonable time sequence after drug administration; following a known standard response of the suspected drug but which may be produced by the characteristics of the individual's clinical condition or other modes of therapy administered to the subject.

4. Remote (Probably Not): A reaction that occurs within a reasonable time sequence after drug administration; which follows a known standard response of the suspected drug but which can be reasonably explained by the characteristics of the individual's clinical condition.

5. Definitely No: Any reaction that does not meet the above criteria.

A 7.7 Medication allowed during the test

There will be no restrictions on the use of symptomatic medications and other diseases with the exception of those listed in the exclusion criteria (tuberculostatic, immunosuppressive and chemotherapy for cancer).

A 7.8 Handling of adverse effects

The AEs shall be recorded in an appropriate form, including: the description of the adverse effect, intensity, relationship to the investigated drug, date of onset, date of termination, duration and conduct taken.

As a general rule, appropriate measures will be taken to deal with AE described in this section.

A 7.9 Monitoring Parameters

The effectiveness and safety parameters (outcomes) will be monitored according to the implementation schedule (item A 7.7).

Ascertainment biases will be minimized through the adoption of a standardized data collection form to be completed at each consultation by the team of trained professionals. This data sheet will include adherence data to the protocol, information on the periodicity of the correct (or not) administration of the drug, the collection of biological samples for exams and the occurrence of adverse effects and outcomes of interest.

A 7.10 Monitoring adherence

Follow-up losses will be bypassed / minimized by active search: by telephone (two) and telegram (one) if no previous response is obtained. These features will be offered to all patients who miss a scheduled appointment. The patient will be asked to return the unused ampules, in order to account for the drug used.

A 7.11 Masking

It was decided that a physician who was not aware of the therapeutic scheme would perform measurements of clinical (interest) and adverse (clinical) outcomes, in order to preserve masking between intervention and outcome. This step was taken to minimize the risk of ascertainment biases originated from differentiated outcomes according to the treatment scheme to which each patient belongs. The results of laboratory tests will be provided by the clinical pathology laboratory without information on the treatment group. Similarly, for analysis purposes, groups will not be identified. The database manager will preserve the confidentiality of this information by encoding the groups for analysis by the epidemiologist(s).

A 7.12 Criteria for definitive discontinuation of study treatment

1. interruption caused by clinical, laboratory or electrocardiographic AE Grade 4

2. interruption longer than 10 days due to clinical, laboratory or electrocardiographic adverse effects Grade <3

3. spontaneous interruption of the use of prescribed medication in an amount greater than five consecutive doses due to failure of administration (non-adherence)

A 7.13 Criteria for withdrawal from the study (but not excluded from data analysis)

1. definitive discontinuation of the treatment regimen for which it was randomized for any reason;

2. pregnancy;

3. need for introduction of immunosuppressive or potentially toxic drug (chemotherapy for cancer, tuberculosis or leprosy scheme);

4. Intercurrent disease, unrelated to the drug studied, but with manifestations equivalent or superior to clinical AD Grade 3;

5. need for re-treatment due to poor initial or late therapeutic response;

6. withdrawal of the patient from continuing in the study.

All patients will receive medical care at the leishmaniasis outpatient clinic of IPEC during the occurrence of adverse events. If necessary, the medication will be suspended for a maximum of 10 days and all affected patients will receive symptomatic treatment until the complete relief of the events temporally associated with the medication. They may have their treatment continued in an alternative regimen (but for the purposes of this clinical trial they will be analyzed by intention to treat, i.e. according to the group for which they were randomized). Whenever treatment is interrupted for safety reasons for more than 10 days, the patient will be withdrawn from the trial and restarted by intralesional treatment (subproject G).

A 7.14 Procedures for breach of confidentiality

Randomization codes generated by software and used in the allocation of numbers and allocation of patients may be unraveled in case of extreme need and always considering

the well-being of the patient. For this purpose, a copy of the randomization scheme will be in possession of an epidemiologist physician not connected to the patient and / or data analysis team, who may be contacted at any time to clarify in case of emergency the type of dose and schedule which the patient belongs.

A 7.15 Study monitoring

A 7.15.1 Coordinators and Field Monitor

The main investigator and coordinators will oversee the fieldwork, controlling for quality and protocol deviations. Important items to monitor are: adequate completion of outcome records and adverse events; adequacy of stored medicines; quality of laboratory examination procedures; minimization of missing data; periodically sending data for typing. Written field reports will be kept for consideration by committees. The main investigator and coordinators will also be in charge of reporting on any serious adverse events to the Research Ethics Committee / IPEC and deciding when to interrupt the trial.

A 7.15.2 External Committee

An external monitoring committee will be constituted in this trial consisting of three expert members in the treatment of leishmaniasis and the execution of clinical trials. Members will be chosen from curriculum Lattes database according to the appropriateness of their function- and competence-based profiles. The committee will audit the documentation and activities pertinent to the clinical trial, assessing possible deviations from the protocol.

A 7.16 Control of dispensing and storage of medications

All ampoules needed for the complete treatment of the entire study population will be stored at the IPEC pharmacy. A trained team professional will recruit the patients at the day 1 appointment, following the randomization list from the trial previously provided by the Epidemiology sector. A trained pharmacist will dispense the medication prescribed by the Infectious Diseases expert physician by submitting the test card and the prescription.

A 7.17 Data Analysis Plan

Data analysis will be performed following the intent-to-treat principle, supplemented by a per-protocol analysis of the primary endpoint. Data from those patients who need a definitive interruption of treatment will be analyzed according to the group to which they were initially allocated, and will not be reassigned in another group to resume treatment (there will be no cross-over between the groups for the purposes of this study). The non-inferiority hypothesis will be tested based on the non-inferiority margin of 15% and a one-sided confidence interval of 95%.

Simple frequencies of categorical variables (gender, race, location of lesions, co-morbidity, adverse events, treatment completion or non-recurrence, relapse) and measures of central tendency and dispersion of continuous quantitative variables (age, number of lesions; time to treatment in days, time to reach the initial and late effectiveness outcomes) for each antimonial scheme used (20 mg or 5 mg) will be described in this study.

Healing frequencies will be compared through the chi-square test, the mean time to healing through a three-way or more (ANOVA) means-comparison test and survival analysis for time-related outcomes in days, non-parametric tests will be used if necessary. To assess effectiveness and safety, relative risk (RR), as well as absolute risk reduction (ARR) and relative risk reduction (RRR) will also be estimated.

For the matched comparison of healing rates on days 20, 30 and 50, the Mann-Whitney test will be used.

A 8. Ethical considerations

A 8.1 Risks and benefits

The main potential benefit of this trial is the possibility of subsidizing the use of lower, potentially less toxic and lower cost antimony doses for the treatment of cutaneous leishmaniasis that affects a large number of Brazilians, including elderly patients with comorbidities (heart, kidney and liver diseases). Risks consist of general adverse effects, which will be carefully scrutinized and treated in accordance with the attached schedule. This project will be submitted to CEP / IPEC and CONEP. All patients will sign an informed consent form approved by CEP / IPEC. This project follows the recommendations contained in resolution 196/96 of the National Health Council.

A 8.2 Term of Free and Informed Consent

In accessible language and clarifying objectives, risk, benefits and identifying those responsible for the research.

A 8.3 Incentives for volunteers

Volunteers will receive transportation assistance and medications.

A 9. Expected Results

It is expected that the alternative regimen with 5mg for 30 days will be non-inferior in effectiveness to the scheme currently recommended in Brazil for the treatment of cutaneous leishmaniasis (20mg Sb5 + / kg / day for 20 days).

However, a significant difference in the toxicity of the different schedules is expected, which should show adverse effects (in frequency and intensity) in the following decreasing order: 1) 20mg Sb5 + / kg / day for 20 days; 2) 5mg Sb5 + / kg / day for 30 days.

Regardless of the therapeutic scheme, lesions located above the knees are expected to be epithelialized at the end of treatment, while the epithelization of localized lesions in the legs and feet occurs more frequently after the end of the administration period. Particularly in those cases with associated vascular insufficiency. It is also expected that, regardless of the therapeutic regimen, patients over 50 years old present adverse effects more frequently and more intensely than younger patients.

The results of this project should be published in indexed journals and in scientific events in the areas of parasitology, molecular biology, infectious diseases and tropical medicine. The coordinators and some researchers will be responsible for organizing the manuscript and communicating the results.

A 10. Financial support

This project is partially funded with resources approved by the Edict MCT / CNPq / MS-SCTIE-DECIT 25/2006 - Neglected Diseases Study - and will be submitted to other relevant edicts of the development agencies.

A 11. Foreign cooperation, storage of biological samples and intellectual property

In this project, there will be no cooperation with foreign entities nor storage of biological samples. There is also no expectation of any patent applications for products and procedures.

BIBLIOGRAPHIC REFERENCES

Adult AIDS Clinical Trials Group August, 1992. AIDS table for grading severity of adult adverse experiences. AACTC. www.aactg.s-3.com. Acessed July 6th, 2004.

Al Jaser M, el-Yazigi A, Croft SL 1995. Pharmacokinetics of antimony in patients treated with sodium stibogluconate for cutaneous leishmaniasis. *Pharmaceutical Research 12*: 113-116.

Alexander J, Russell DG 1992. The interaction of Leishmania species with macrophages. *Adv Parasitol 31*: 175-254.

Amato V, Amato J, Nicodemo A, Uip D, Amato-Neto V, Duarte M 1998. [Treatment of mucocutaneous leishmaniasis with pentamidine isothionate]. *Ann Dermatol Venereol 125*: 492-495.

Andrade L, Machado C, Chiari E, Pena S, Macedo A 1999. Differential tissue distribution of diverse clones of *Trypanosoma cruzi* in infected mice. *Mol Biochem Parasitol 100*: 163-172.

Antezana G, Zeballos R, Mendoza C, Lyevre P, Valda L, Cardenas F, Noriega I, Ugarte H, Dedet JP 1992. Electrocardiographic alterations during treatment of mucocutaneous leishmaniasis with meglumine antimoniate and allopurinol. *Trans R Soc Trop Med Hyg 86*: 31-33.

Antoine JC, Prina E, Jouanne C, Bongrand P 1990. Parasitophorous vacuoles of Leishmania amazonensis-infected macrophages maintain an acidic pH. *Infect Immun 58*: 779-787.

Aronson NE, Wortmann GW, Johnson SC, Jackson JE, Gasser Jr RA, Magill AJ, Endy TP, Coyne PE, Grogl M, Benson PM, Beard JS, Tally JD, Gambel JM, Kreutzer RD, Oster CN 1998. Safety and efficacy of intravenous sodium stibogluconate in the treatment of leishmaniasis: recent U.S. military experience. *Clinical Infectious Diseases 27*: 1457-1464.

Ashford RW 2000. The leishmaniases as emerging and reemerging zoonoses. *Int J Parasitol 30*: 1269-1281.

Aviles H, Belli A, Armijos R, Monroy FP, Harris E 1999. PCR detection and identification of Leishmania parasites in clinical specimens in Ecuador: a comparison with classical diagnostic methods. *J Parasitol 85*: 181-187.

Azeredo-Coutinho RB, Mendonca SC 2002. An intermittent schedule is better than continuous regimen of antimonial therapy for cutaneous leishmaniasis in the municipality of Rio de Janeiro, Brazil. *Revista da Sociedade Brasileira de Medicina Tropical 3*: 477-481.

Azeredo-Coutinho RB, Mendonça SCF 1997. Comparative study of two antimonial therapy schedules for treating cutaneous leishmaniasis. In XXIV Annual Meeting on Basic Research in Chagas Disease, Caxambu, 92, Memórias do Instituto Oswaldo Cruz, 11-14 November 1997.

Azeredo-Coutinho RBG 1999. Estudo comparativo de dois esquemas de tratamento antimonial da leishmaniose cutânea causada por Leishmania braziliensis no município do Rio de Janeiro*. Curso de Pós-graduação em Medicina Tropical*. Instituto Oswaldo Cruz, Fiocruz, Rio de Janeiro, p. 75.

Bacellar O, Lessa H, Schriefer A, Machado P, Ribeiro de Jesus A, Dutra WO, Gollob KJ, Carvalho EM 2002. Up-regulation of Th1-type responses in mucosal leishmaniasis patients. *Infect Immun 70*: 6734-6740.

Barkirtzief Z 1996. Identificando barreiras para aderência no tratamento da hanseníase. *Cadernos de Saúde Pública 12*: 497-505.

Barral-Netto M, Machado P, Bittencourt A, Barral A 1997. Recent advances in the pathophysiology and treatment of human cutaneous leishmaniasis. *Current Opinion in Dermatology 4*: 51-58.

Barros MBL, Schubach A, Francesconi-do-Valle AC, Gutierrez-Galhardo MC, Schubach TMP, Conceição-Silva F, Salgueiro MM, Mouta-Confort E, Reis RS, Madeira MF, Cuzzi T, Quintella LP, Passos JPS, Conceição MJ, Marzochi MCA 2005. Positive Montenegro skin test among patients with sporotrichosis in Rio de Janeiro. *Acta Tropica 93*: 41-47.

Beers MH 1997. Medicamentos para idosos. In PR Katz, *Geriatria Prática*, Revinter, Rio de Janeiro, p. 34-52.

Belli A, Rodriguez B, Aviles H, Harris E 1998. Simplified polymerase chain reaction detection of new world Leishmania in clinical specimens of cutaneous leishmaniasis. *Am J Trop Med Hyg 58*: 102-109.

Berman JD 1988. Chemotherapy for leishmaniasis: biochemical mechanisms, clinical efficacy, and future strategies. *Reviews Infectious Diseases 10*: 560-586.

Berman JD, Chulay JD, Hendricks LD, Oster CN 1982. Susceptibility of clinically sensitive and resistant Leishmania to pentavalent antimony in vitro. *Am J Trop Med Hyg 31*: 459-465.

Berman JD, Gallalee JF, Gallalee JV 1988. Pharmacokinetics of pentavalent antimony (Pentostam) in hamsters. *Am J Trop Med Hyg 39*: 41-45.

Berman JD, Lee LS 1983. Activity of oral drugs against Leishmania tropica in human macrophages in vitro. *Am J Trop Med Hyg 32*: 947-951.

Berman JD, Waddell D, Hanson BD 1985. Biochemical mechanisms of the antileishmanial activity of sodium stibogluconate. *Antimicrob Agents Chemother 27*: 916-920.

Berman JD, Wyler DJ 1980. An in vitro model for investigation of chemotherapeutic agents in leishmaniasis. *J Infect Dis 142*: 83-86.

Beverley SM, Ismach RB, Pratt DM 1987. Evolution of the genus Leishmania as revealed by comparisons of nuclear DNA restriction fragment patterns. *Proc Natl Acad Sci U S A 84*: 484-488.

Beverley SM, Turco SJ 1998. Lipophosphoglycan (LPG) and the identification of virulence genes in the protozoan parasite Leishmania. *Trends Microbiol 6*: 35-40.

Botega N 2001. *Prática psiquiátrica no hospital geral*. Artmed, Porto Alegre.

Brasil, Ministério da Saúde, Fundação Nacional de Saúde 2000. *Manual de Controle da Leishmaniose Tegumentar Americana*, Brasília, 62 pp.

Brasil, Ministério da Saúde, Fundação Nacional de Saúde, Centro Nacional de Epidemiologia, Coordenação Nacional de Dermatologia Sanitária 1997. Relatório da Oficina de Trabalho de Leishmanioses, Brasília.

Breniere SF, Telleria J, Bosseno MF, Buitrago R, Bastrenta B, Cuny G, Banuls AL, Brewster S, Barker DC 1999. Polymerase chain reaction-based identification of New World Leishmania species complexes by specific kDNA probes. *Acta Trop 73*: 283-293.

Brochu C, Wang J, Roy G, Messier N, Wang XY, Saravia NG, Ouellette M 2003. Antimony uptake systems in the protozoan parasite *Leishmania* and accumulation differences in antimony-resistant parasites. *Antimicrob Agents Chemother 47*: 3073-3079.

Brummitt CF, Porter JA, Herwaldt BL 1996. Reversible peripheral neuropathy associated with sodium stibogluconate therapy for American cutaneous leishmaniasis. *Clin Infect Dis 22*: 878-879.

Bryceson AD, Bray RS, Wolstencroft RA, Dumonde DC 1970. Cell mediated immunity in cutaneous leishmaniasis of the guinea-pig. *Trans R Soc Trop Med Hyg 64*: 472.

Bryceson AD, Chulay JD, Ho M, Mugambii M, Were JB, Muigai R, Chunge C, Gachihi G, Meme J, Anabwani G, et al. 1985a. Visceral leishmaniasis unresponsive to antimonial drugs. I. Clinical and immunological studies. *Trans R Soc Trop Med Hyg 79*: 700-704.

Bryceson AD, Chulay JD, Mugambi M, Were JB, Gachihi G, Chunge CN, Muigai R, Bhatt SM, Ho M, Spencer HC, Meme J, Anabwani G 1985b. Visceral leishmaniasis unresponsive to antimonial drugs. II. Response to high dosage sodium stibogluconate or prolonged treatment with pentamidine. *Trans R Soc Trop Med Hyg 79*: 705-714.

Cabrera M, Blackwell JM, Castes M, Trujillo D, Convit J, Shaw MA 2000. Immunotherapy with live BCG plus heat killed Leishmania induces a T helper 1-like response in American cutaneous leishmaniasis patients. *Parasite Immunol 22*: 73-79.

Cabrine-Santos M, Silva E, Chapadeiro E, Ramírez L 2001. *Trypanosoma cruzi*: characterization of reinfection and search for tissue tropism in hamsters (*Mesocricetus auratus*). *Exp Parasitol 99*: 160-167.

Callahan HL, Beverley SM 1991. Heavy metal resistance: a new role for P-glycoproteins in Leishmania. *J Biol Chem 266*: 18427-18430.

Callahan HL, Roberts WL, Rainey PM, Beverley SM 1994. The PGPA gene of Leishmania major mediates antimony (SbIII) resistance by decreasing influx and not by increasing efflux. *Mol Biochem Parasitol 68*: 145-149.

Carvalho EM, Barral A, Costa JM, Bittencourt A, Marsden P 1994. Clinical and immunopathological aspects of disseminated cutaneous leishmaniasis. *Acta Trop 56*: 315-325.

Carvalho EM, Teixeira RS, Johnson WD, Jr. 1981. Cell-mediated immunity in American visceral leishmaniasis: reversible immunosuppression during acute infection. *Infect Immun 33*: 498-500.

Carvalho JAM, Garcia RA 2003. [The aging process in the Brazilian population: a demographic approach]. *Cadernos de Saúde Pública 19*: 725-733.

Castes M, Agnelli A, Verde O, Rondon AJ 1983. Characterization of the cellular immune response in American cutaneous leishmaniasis. *Clin Immunol Immunopathol 27*: 176-186.

Castes M, Cabrera M, Ujillo DT, Convit J 1988. T-cell subpopulations, expression of interleukin-2 receptor, and production of interleukin-2 and gamma interferon in human American cutaneous leishmaniasis. *Journal of Clinical Microbiology 26*: 1207-1213.

Castes M, Moros Z, Martinez A, Trujillo D, Castellanos PL, Rondon AJ, Convit J 1989. Cell-mediated immunity in localized cutaneous leishmaniasis patients before and

after treatment with immunotherapy or chemotherapy. *Parasite Immunol 11*: 211-222.

CENEPI 1997. *Informe Epidemiológico do SUS*

Chicharro C, Morales MA, Serra T, Ares M, Salas A, Alvar J 2002. Molecular epidemiology of Leishmania infantum on the island of Majorca: a comparison of phenotypic and genotypic tools. *Trans R Soc Trop Med Hyg 96 Suppl 1*: S93-99.

Chulay JD, Fleckenstein L, Smith DH 1988. Pharmacokinetics of antimony during treatment of visceral leishmaniasis with sodium stibogluconate or meglumine antimoniate. *Trans R Soc Trop Med Hyg 82*: 69-72.

Chulay JD, Spencer HC, Mugambi M 1985. Electrocardiographic changes during treatment of leishmaniasis with pentavalent antimony (sodium stibogluconate). *Am J Trop Med Hyg 34*: 702-709.

Claros P, Wienberg P, Gonzalez MA, Claros A, Claveria MA, Lopez P 1996. [Intralesional treatment of cutaneous leishmaniasis: a report of two cases]. *Acta Otorrinolaringol Esp 47*: 67-70.

Conceição-Silva F, Dorea RC, Pirmez C, Schubach A, Coutinho SG 1990. Quantitative study of *Leishmania braziliensis braziliensis* reactive T cells in peripheral blood and in the lesions of patients with American mucocutaneous leishmaniasis. *Clin Exp Immunol 79*: 221-226.

Convit J, Pinardi ME, Rondon AJ 1972. Diffuse cutaneous leishmaniasis: a disease due to an immunological defect of the host. *Trans R Soc Trop Med Hyg 66*: 603-610.

Correia D, Macedo VO, Carvalho EM, Barral A, Magalhaes AV, de Abreu MV, Orge ML, Marsden P 1996. [Comparative study of meglumine antimoniate, pentamidine isethionate and aminosidine sulfate in the treatment of primary skin lesions caused by Leishmania (Viannia) braziliensis]. *Rev Soc Bras Med Trop 29*: 447-453.

Costa JM, Vale KC, Franca F, Saldanha AC, Silva JO, Lago EL, Marsden PD, Magalhães AV, Silva CM, Serra Neto A 1990. [Spontaneous healing of leishmaniasis caused by *Leishmania Viannia braziliensis* in cutaneous lesions]. *Revista da Sociedade Brasileira de Medicina Tropical 23*: 205-208.

Coutinho SG, Da-Cruz AM, Bertho AL, Santiago MA, De-Luca P 1998. Immunologic patterns associated with cure in human American cutaneous leishmaniasis. *Braz J Med Biol Res 31*: 139-142.

Coutinho SG, Pirmez C, Da-Cruz AM 2002. Parasitological and immunological follow-up of American tegumentary leishmaniasis patients. *Trans R Soc Trop Med Hyg 96 Suppl 1*: S173-178.

Coutinho SG, Pirmez C, Mendonca SCF, Conceição-Silva F, Dorea RCC 1987. Pathogenesis and immunopathology of leishmaniasis. *Memórias do Instituto Oswaldo Cruz 82*: 214-228.

Cramer J, Mattson R, Prevey M, Scheyer R, Ouellette V 1989. How often is medication taken as prescribed? *JAMA 261*: 3273-3277.

Crofts MA 1976. Use of amphotericin B in mucocutaneous leishmaniasis. *J Trop Med Hyg 79*: 111-113.

Croop J, Gros P, Housman D 1988. Genetics of multidrug resistance. *Journal of Clinical Investigation 81*: 1303-1309.

Cullen W, McBride B, Reglinski J 1984. The reaction of methylarsenicals with thiols: Some biological implications. *Journal of Inorganic Biochemistry 21*: 179-194.

Cupolillo E, Brahim LR, Toaldo CB, de Oliveira-Neto MP, de Brito ME, Falqueto A, de Farias Naiff M, Grimaldi G, Jr. 2003. Genetic polymorphism and molecular epidemiology of Leishmania (Viannia) braziliensis from different hosts and geographic areas in Brazil. *J Clin Microbiol 41*: 3126-3132.

Cupolillo E, Grimaldi Jr. G, Momen H 1994. A general classificatiom of new world *Leishmania* using numeral zymotaxomomy. *American Journal Tropical Medicine Hygiene 50*: 296-311.

Da Silva RP, Hall BF, Joiner KA, Sacks DL 1989. CR1, the C3b receptor, mediates binding of infective Leishmania major metacyclic promastigotes to human macrophages. *J Immunol 143*: 617-622.

Da-Cruz AM, de Oliveira MP, De Luca PM, Mendonca SC, Coutinho SG 1996. Tumor necrosis factor-alpha in human american tegumentary leishmaniasis. *Mem Inst Oswaldo Cruz 91*: 225-229.

Da-Cruz AM, Machado ES, Menezes JA, Rutowitsch MS, Coutinho SG 1992. Cellular and humoral immune responses of a patient with American cutaneous leishmaniasis and AIDS. *Trans R Soc Trop Med Hyg 86*: 511-512.

de Bruijn MH, Barker DC 1992. Diagnosis of New World leishmaniasis: specific detection of species of the Leishmania braziliensis complex by amplification of kinetoplast DNA. *Acta Trop 52*: 45-58.

Deane LM, Grimaldi Jr. G 1985. Leishmaniasis in Brazil. In RS Bray, *Leishmania*, Elsevier, Amsterdan, p. 247-281.

Degrave W, Fernandes O, Campbell D, Bozza M, Lopes U 1994. Use of molecular probes and PCR for detection and typing of *Leishmania* - a mini-review. *Memórias do Instituto Oswaldo Cruz 89*: 463-469.

Deps PD, Viana MC, Falqueto A, Dietze R 2000. [Comparative assessment of the efficacy and toxicity of N-methyl- glucamine and BP88 sodium stibogluconate in the

treatment of localized cutaneous leishmaniasis]. *Revista da Sociedade Brasileira de Medicina Tropical 33*: 535-543.

Desjardins M, Descoteaux A 1997. Inhibition of phagolysosomal biogenesis by the Leishmania lipophosphoglycan. *J Exp Med 185*: 2061-2068.

Desjeux P 2001. The increase in risk factors for leishmaniasis worldwide. *Trans R Soc Trop Med Hyg 95*: 239-243.

D'Oliveira A, Jr., Machado P, Bacellar O, Cheng LH, Almeida RP, Carvalho EM 2002. Evaluation of IFN-gamma and TNF-alpha as immunological markers of clinical outcome in cutaneous leishmaniasis. *Rev Soc Bras Med Trop 35*: 7-10.

Dujardin JC, Victoir K, De Doncker S, Guerbouj S, Arevalo J, Le Ray D 2002. Molecular epidemiology and diagnosis of Leishmania: what have we learnt from genome structure, dynamics and function? *Trans R Soc Trop Med Hyg 96 Suppl 1*: S81-86.

Durbar-Jacob J, Mortimer-Stephens MK 2001. Treatment adherence in chronic disease. *J Clin Epidemiol 54*: S57-S60.

D'Utra e Silva O 1915. Sobre a leishmaniose tegumentar e seu tratamento. *Memórias do Instituto Oswaldo Cruz 7*: 213-248.

Falqueto A, Sessa PA 1997. Leishmaniose Tegumentar Americana. In R Focaccia, *Veronesi Tratado de Infectologia*, Atheneu, São Paulo, p. 1221-1233.

Feldmann J, Haas K 2000. Sampling of trace volatile metal(loid) compounds in ambient air using polymer bags: A convenient method. *Anal Chem 72*: 4205-4211.

Fernandes O, Murthy VK, Kurath U, Degrave WM, Campbell DA 1994. Mini-exon gene variation in human pathogenic Leishmania species. *Mol Biochem Parasitol 66*: 261-271.

Ferreira-Pinto KC, Miranda-Vilela AL, Anacleto C, Fernandes AP, Abdo MC, Petrillo-Peixoto ML, Moreira ES 1996. Leishmania (V.) guyanensis: isolation and characterization of glucantime-resistant cell lines. *Can J Microbiol 42*: 944-949.

Filella M, Belzile N, Chen Y 2002. Antimony in the environment: a review focused on natural waters - I. Ocurrence. *Earth Sciences Reviews 57*: 125-176.

Filgueiras S, Deslandes S 1999. Avaliação das ações de aconselhamento. Análise de uma perspectiva de prevenção centrada na pessoa. *Cadernos de Saúde Pública 15*: 121-131.

Fletcher R, Fletcher S, Wagner E 1989. *Epidemiologia clínica*. Artes Médicas, Porto Alegre.

Franco D, Vago A, Chiari E, Meira F, Galvão L, Machado C 2003. *Trypanosoma cruzi*: mixture of two populations can modify virulence and tissue tropism in rat. *Exp Parasitol 104*: 54-61.

Franke ED, Wignall FS, Cruz ME, Rosales E, Tovar AA, Lucas CM, Llanos-Cuentas A, Berman JD 1990. Efficacy and toxicity of sodium stibogluconate for mucosal leishmaniasis. *Annals Internal Medicine 113*: 934-940.

Fundação Nacional de Saúde 2000. *Manual de Controle da Leishmaniose Tegumentar Americana, Ministério da Saúde*, Brasília, 62 pp.

Furtado T 1994. Leishmaniose Tegumentar Americana. In J Machado-Pinto, *Doenças infecciosas com manisfestações dermatológicas*, Editora Médica e Científica Ltda, Rio de Janeiro, p

Garcia R 2003. Os fatores de aderência ao tratamento farmacológico das hiperlipidemias em pacientes atendidos na Secretaria Municipal de Ribeirão Preto. *Faculdade de Medicina de Ribeirão Preto*. Universidade de São Paulo, Ribeirão Preto, p. 104.

Gebel T 1997. Arsenic and antimony: comparative approach on mechanistic toxicology. *Chemico-Biological Interactions 107*: 131-144.

Gebel T 1998. Human biomonitoring of arsenic and antimony in case of an elevated geogenic exposure. *Environmental Health Perspectives 106*: 33-39.

Georges E, Bradley G, Gariepy J, Ling V 1990. Detection of P-glycoprotein isoforms by gene-specific monoclonal antibodies. *Proc Natl Acad Sci U S A 87*: 152-156.

Gervasio A, Lavorante A, Moraes M, Giné M, Miranda C, Carrilho E 2003. Eletroforese capilar acoplada à espectrometria com plasma: uma ferramenta eficiente para a especiação. *Química Nova 26*: 65-74.

Giatti L, Barreto SM 2003. [Health, work, and aging in Brazil]. *Cadernos de Saúde Pública 19*: 759-771.

Gil V, Paya M, Asensio M, Torres M, Pastor R, Merino J 1999. Incumplimiento del tratamiento con antibióticos en infecciones agudas no graves. *Med Clin 112*: 731-773.

Goldberg A, Cohen G, Rubin A 1998. Physician assessment of patient compliance with compliance treatment. *Soc Sci Med 47*: 1873-1876.

Gomes M, Silva E, Macedo A, Vago A, Melo M 1997. LSSP-PCR for characterization of strains of *Entamoeba histolytica* isolated in Brazil. *Parasitology*: 517-520.

Gomes RF, Macedo AM, Pena SD, Melo MN 1995. Leishmania (Viannia) braziliensis: genetic relationships between strains isolated from different areas of Brazil as revealed by DNA fingerprinting and RAPD. *Exp Parasitol 80*: 681-687.

Goodwin LG 1995. Pentostan (sodium stibogluconate); a 50-year personal reminiscence. *Transactions Royal Society Tropical Medicine Hygiene 89*: 339-341.

Grimaldi G, Jr., Tesh RB 1993. Leishmaniases of the New World: current concepts and implications for future research. *Clin Microbiol Rev 6*: 230-250.

Grimaldi G, McMahon-Pratt D 1996. Monoclonal antibodies for the identification of New World *Leishmania* species. *Memórias do Instituto Oswaldo Cruz 91*: 37-42.

Grogl M, Martin RK, Oduola AM, Milhous WK, Kyle DE 1991. Characteristics of multidrug resistance in Plasmodium and Leishmania: detection of P-glycoprotein-like components. *Am J Trop Med Hyg 45*: 98-111.

Grogl M, Oduola AM, Cordero LD, Kyle DE 1989. *Leishmania* spp.: development of pentostam-resistant clones in vitro by discontinuous drug exposure. *Exp Parasitol 69*: 78-90.

Grogl M, Thomason TN, Franke ED 1992. Drug resistance in leishmaniasis: its implication in systemic chemotherapy of cutaneous and mucocutaneous disease. *Am J Trop Med Hyg 47*: 117-126.

Guerbouj S, Victoir K, Guizani I, Seridi N, Nuwayri-Salti N, Belkaid M, Ismail RB, Le Ray D, Dujardin JC 2001. Gp63 gene polymorphism and population structure of Leishmania donovani complex: influence of the host selection pressure? *Parasitology 122 Pt 1*: 25-35.

Guevara P, Alonso G, Silveira JF, Mello M, Scorza JV, Añez N, Ramirez JL 1992. Identification of new world *Leishmania* using ribosomal gene spacer probes. *Molecular and Biochemical Parasitology 56*: 15-26.

Gupta P 1990. Electrocardiographic changes occurring after brief antimony administration in the presence of dilated cardiomyopathy. *Postgrad Med J 66*: 1089.

Halim MA, Alfurayh O, Kalin ME, Dammas S, al-Eisa A, Damanhouri G 1993. Successful treatment of visceral leishmaniasis with allopurinol plus ketoconazole in a renal transplant recipient after the occurrence of pancreatitis due to stibogluconate. *Clinical Infectious Diseases 16*: 397-399.

Hanafi R, Barhoumi M, Ali SB, Guizani I 2001. Molecular analyses of Old World Leishmania RAPD markers and development of a PCR assay selective for parasites of the L. donovani species Complex. *Exp Parasitol 98*: 90-99.

Handman E, Goding JW 1985. The Leishmania receptor for macrophages is a lipid-containing glycoconjugate. *Embo J 4*: 329-336.

Harms G, Chehade AK, Douba M, Roepke M, Mouakeh A, Rosenkaimer F, Bienzle U 1991. A randomized trial comparing a pentavalent antimonial drug and recombinant interferon-gamma in the local treatment of cutaneous leishmaniasis. *Trans R Soc Trop Med Hyg 85*: 214-216.

Haynes R 1981a. Determinants of compliance: the disease end mechanics of treatment. In R Haynes, D Taylor, D Sackett (eds), *Compliance in health care*, The Johns Hopkins University Press, Baltimore, p. 49-61.

Haynes R 1981b. Introduction. In R Haynes, D Taylor, D Sackett (eds), *Compliance in health care*, The Johns Hopkins University Press, Baltimore, p. 1-7.

Hepburn NC 2000. Cutaneous leishmaniasis. *Clin Exp Dermatol 25*: 363-370.

Hepburn NC, Nolan J, Fenn L, Herd RM, Neilson JM, Sutherland GR, Fox KA 1994a. Cardiac effects of sodium stibogluconate: myocardial, electrophysiological and biochemical studies. *Qjm 87*: 465-472.

Hepburn NC, Siddique I, Howie AF, Beckett GJ, Hayes PC 1994b. Hepatotoxicity of sodium stibogluconate therapy for American cutaneous leishmaniasis. *Trans R Soc Trop Med Hyg 88*: 453-455.

Herwaldt BL 1999. Leishmaniasis. *Lancet 354*: 1191-1199.

Herwaldt BL, Berman JD 1992. Recommendations for treating leishmaniasis with sodium stibogluconate (Pentostam) and review of pertinent clinical studies. *American Journal of Tropical Medicine and Hygiene 46*: 296-306.

IBGE 1981. *Censo Demográfico 1980*. Fundação Instituto Brasileiro de Geografia e Estatística (IBGE), Rio de Janeiro.

IBGE 2001. *Censo Demográfico 2000*. Fundação Instituto Brasileiro de Geografia e Estatística (IBGE), Rio de Janeiro.

Ishikawa EA, Silveira FT, Magalhaes AL, Guerra junior RB, Melo MN, Gomes R, Silveira TG, Shaw JJ 2002. Genetic variation in populations of Leishmania species in Brazil. *Trans R Soc Trop Med Hyg 96 Suppl 1*: S111-121.

Jackson PR, Lawrie JM, Stiteler JM, Hawkins DW, Wohlhieter JA, Rowton ED 1986. Detection and characterization of Leishmania species and strains from mammals and vectors by hybridization and restriction endonuclease digestion of kinetoplast DNA. *Vet Parasitol 20*: 195-215.

Jones TC, Johnson WD, Jr., Barretto AC, Lago E, Badaro R, Cerf B, Reed SG, Netto EM, Tada MS, Franca TF, Wiese K, Golightly L, Fikrig E, Costa JML, Cuba CC, Marsden PD 1987. Epidemiology of American cutaneous leishmaniasis due to *Leishmania braziliensis braziliensis*. *Journal of Infectious Diseases 156*: 73-83.

Krachler M, Emons H 2001. Speciation analysis of antimony by high-performance liquid chromatography inductively coupled plasma mass spectrometry using ultrasonic nebulization. *Analytica Chimica Acta 429*: 125-133.

Kubba R, al-Gindan Y, el-Hassan AM, Omer AH, Kutty MK, Saeed MB 1988. Dissemination in cutaneous leishmaniasis. II. Satellite papules and subcutaneous induration. *Int J Dermatol 27*: 702-706.

Kubba R, el-Hassan AM, Al-Gindan Y, Omer AH, Kutty MK, Saeed MB 1987. Dissemination in cutaneous leishmaniasis. I. Subcutaneous nodules. *Int J Dermatol 26*: 300-304.

Kurita G, Pimenta C 2003. Adesão ao tratamento da dor crônica: estudo de varáveis demográficas, terapêuticas e psicossociais. *Arq Neuropsiquiatr 61*: 416-425.

Lainson R 1983. The American leishmaniases: some observations on their ecology and epidemiology. *Transactions Royal Society Tropical Medicine Hygiene 77*: 569-596.

Lainson R, Shaw JJ 1987. Evolution, classification and geographical distribution. In K Killick-Kendrick, *The leishmaniasis in biology and medicine*, Academic Press, London, p. 1-120.

Legare D, Hettema E, Ouellette M 1994. The P-glycoprotein-related gene family in *Leishmania*. *Mol Biochem Parasitol 68*: 81-91.

Legare D, Papadopoulou B, Roy G, Mukhopadhyay R, Haimeur A, Dey S, Grondin K, Brochu C, Rosen BP, Ouellette M 1997. Efflux systems and increased trypanothione levels in arsenite-resistant Leishmania. *Exp Parasitol 87*: 275-282.

Leite J, Dracheler M, Centeno M, Pinheiro C, Silveira V 2002. Desenvolvimento de uma escala de auto-eficácia para adesão ao tratamento anti-retroviral. *Psicol Reflex Crit 15*: 121-133.

Leite S, Vasconcellos M 2003. Adesão à terapêutica medicamentosa: elementos para discussão de conceitos e pressupostos adotados na literatura. *Ciência & Saúde Coletiva 8*: 775-782.

Levy R, Feld A 1999. Increasing patient adherence to gastroenterology treatment and prevention regimens. *Am J Gastroenterol 94*: 1733-1742.

Llanos-Cuentas EA, Arana M, Cuba CAC, Rosa AC, Marsden PD 1985. Leishmaniasis cutanea diseminada asociada a metastasis en mucosas, causada por *Leishmania braziliensis braziliensis*: fracaso en el hallazgo de parasitos circulantes. *Rev Soc Bras Med Trop 18*: 271-272.

Lopes UG, Momen H, Grimaldi Jr. G, Marzochi MCA, Pacheco RS, Morel CM 1984. Schizodeme and zymodeme characterization of *Leishmania* in the investigation of focci of visceral and cutaneous leishmaniasis. *Journal of Parasitology 70*: 89-98.

Lopez M, Inga R, Cangalaya M, Echevarria J, Llanos-Cuentas A, Orrego C, Arevalo J 1993. Diagnosis of Leishmania using the polymerase chain reaction: a simplified procedure for field work. *Am J Trop Med Hyg 49*: 348-356.

Lyons LW, Johnston CB, Covinsky KE, Resnick NM 2001. Geriatric Medicine. In MA Papadakis, *Current Medical Diagnosis & Treatment*, McGraw-Hill, New York, p. 44-61.

Machado P, Araujo C, Da Silva AT, Almeida RP, D'Oliveira Jr A, Bittencourt A, Carvalho EM 2002. Failure of early treatment of cutaneous leishmaniasis in preventing the development of an ulcer. *Clin Infect Dis 34*: E69-73.

Marsden PD 1979. Current concepts in parasitology. Leishmaniasis. *N Engl J Med 300*: 350-352.

Marsden PD 1985. Pentavalent antimonials: old drugs for new diseases. *Revista da Sociedade Brasileira de Medicina Tropical 18*: 187-198.

Marsden PD 1986. Mucosal leishmaniasis ("espundia" Escomel, 1911). *Trans R Soc Trop Med Hyg 80*: 859-876.

Marsden PD, Jones TC 1985. Clinical manifestations, diagnosis and treatment of leishmaniasis. In RS Bray, *Leishmaniasis*, Elsevier, London, p. 183-198.

Marsden PD, Netto EM, Badaro R, Cuba CA, Costa JL, Barreto AC 1986. Apparent cure of a difficult treatment problem in a patient with mucosal leishmaniasis. *Am J Trop Med Hyg 35*: 449.

Marsden PD, Tada MS, Barreto AC, Cuba CC 1984. Spontaneous healing of Leishmania braziliensis braziliensis skin ulcers. *Trans R Soc Trop Med Hyg 78*: 561-562.

Martinez E, Alonso V, Quispe A, Thomas MC, Alonso R, Pinero JE, Gonzalez AC, Ortega A, Valladares B 2003. RAPD method useful for distinguishing Leishmania species: design of specific primers for L. braziliensis. *Parasitology 127*: 513-517.

Marzochi MAC, Marzochi KBF 1994. Tegumentary and visceral leishmaniasis in Brazil. Emerging anthropozoonosis and possibilities for their control. *Cadernos de Saúde Pública 10*: 359-375.

Marzochi MC, Coutinho SG, De Souza WJ, De Toledo LM, Grimaldi Junior G, Momen H, Pacheco R, Sabroza PC, De Souza MA, Rangel Junior FB, Tramontano NC 1985. Canine visceral leishmaniasis in Rio de Janeiro, Brazil. Clinical, parasitological, therapeutical and epidemiological findings (1977-1983). *Memórias do Instituto Oswaldo Cruz 80*: 349-357.

Marzochi MCA 1992. Leishmanioses no Brasil: As leishmanioses tegumentares. *Jornal Brasileiro de Medicina 63*: 82-104.

Matos DS, Azeredo-Coutinho RB, Schubach A, Conceição-Silva F, Baptista C, Moreira JS, Mendonça SC 2005. Differential interferon- gamma production characterizes the cytokine responses to Leishmania and Mycobacterium leprae antigens in concomitant mucocutaneous leishmaniasis and lepromatous leprosy. *Clinical Infectious Diseases 40*: e5-12.

McBride MO, Linney M, Davidson RN, Weber JN 1995. Pancreatic necrosis following treatment of leishmaniasis with sodium stibogluconate. *Clin Infect Dis 21*: 710.

McMahon-Pratt D, David JR 1981. Monoclonal antibodies that distinguish between New World species of Leishmania. *Nature 291*: 581-583.

McMahon-Pratt D, Jaffe CL, Bennett E, David JR, Grimaldi Jr. G 1986. Studies employing monoclonal antibodies for the analysis of the genus *Leishmania* Ross, 1903. In JA Rioux, *Leishmania Taxonomy and Phylogeny*, IMEEE, Montpellier, p. 173-178.

Mendonça MG, De Brito ME, Rodrigues EH, Bandeira V, Jardim ML, Abath FG 2004. Persistence of leishmania parasites in scars after clinical cure of american cutaneous leishmaniasis: is there a sterile cure? *J Infect Dis 189*: 1018-1023.

Mendonça SC, Coutinho SG, Amendoeira RR, Marzochi MC, Pirmez C 1986. Human American cutaneous leishmaniasis (*Leishmania b. braziliensis*) in Brazil: lymphoproliferative responses and influence of therapy. *Clinical and Experimental Immunology 64*: 269-276.

Mendonça SC, Russell DG, Coutinho SG 1991. Analysis of the human T cell responsiveness to purified antigens of Leishmania: lipophosphoglycan (LPG) and glycoprotein 63 (gp 63). *Clinical Experimental Immunology 83*: 472-478.

Mendonça SC, Souza WJ, Nunes MP, Marzochi MC, Coutinho SG 1988. Indirect immunofluorescence test in New World leishmaniasis: serological and clinical relationship. *Mem Inst Oswaldo Cruz 83*: 347-355.

Mendoza-Leon A, Havercroft JC, Barker DC 1995. The RFLP analysis of the beta-tubulin gene region in New World Leishmania. *Parasitology 111*: 1-9.

Miekeley N, Mortari SR, Schubach AO 2002. Monitoring of total antimony and its species by ICP-MS and on-line ion chromatography in biological samples from patients treated for leishmaniasis. *Analytical Bioanalytical Chemistry 372*: 495-502.

Misago C, Marshall T, Fonseca W, Kikwood B 1997. Out-patient drug treatment of pneumonia among children under two years of age in Fortaleza, Brazil. *Cadernos de Saúde Pública 13*: 37-43.

Momen H, Grimaldi Jr G, Pacheco RS, Jaffe CL, McMahon-Pratt D, Marzochi MC 1985. Brazilian *Leishmania* stocks phenotypically similar to *Leishmania major*. *American Journal Tropical Medicine Hygiene 34*: 1076-1084.

Monjour L, Neogy AB, Vouldoukis I, Silva OA, Boisnic S, Brito ME, Lesot A, Vignot N, Martins JS, Jardim ML 1994. Exploitation of parasite derived antigen in therapeutic success of human cutaneous leishmaniasis in Brazil. *Mem Inst Oswaldo Cruz 89*: 479-483.

Moreira JS 1994. Tese. Estudo da Laringite Leishmaniótica*. Departamento de Cirurgia*. Pontifícia Universidade Católica do Rio de Janeiro, Rio de Janeiro, p. 118.

Morisky D, Levine M, Green L, Smith C 1982. Health education program effects on the managements of hipertension in the elderly. *Archives Internal Medicine 172*: 1335-1838.

Mortari SR 2001. Determinação da concentração total de antimônio e de suas espécies químicas em amostras clínicas de pacientes com leishmanioses. *Departamento de Química*. Pontifícia Universidade Católica (PUC), Rio de Janeiro, p. 142.

Nakashima S 1980. Selective determination of antimony(III) and antimony(V) by atomic-absorption spectrophotometry following stibine generation. *Analyst 105*: 732-733.

Navin TR, Arana BA, Arana FE, Berman JD, Chajon JF 1992. Placebo-controlled clinical trial of sodium stibogluconate (Pentostam) versus ketoconazole for treating cutaneous leishmaniasis in Guatemala. *Journal of Infectious Diseases 165*: 528-534.

Nigro G, Angelini G, Grosso S, Caula G, Sategna-Guidetti C 2001. Psychatric predictors of non compliance in inflammatory bowel disease. *J Clin Gastroenterol 32*: 61-68.

Oliveira FS, Pirmez C, Pires MQ, Brazil RP, Pacheco RS 2005. PCR-based diagnosis for detection of Leishmania in skin and blood of rodents from an endemic area of cutaneous and visceral leishmaniasis in Brazil. *Vet Parasitol 129*: 219-227.

Oliveira M, Caballero O, Vago A, Harskeerl R, Romanha A, Pena S, Simpson A, Koury M 2003. Low-stringency single specific primer PCR for identification of *Leptospira*. *J Med Microbiol 52*: 127-135.

Oliveira-Neto MP, Mattos M, Pirmez C, Fernandes O, Goncalves-Costa SC, Souza CF, Grimaldi G, Jr. 2000. Mucosal leishmaniasis ("espundia") responsive to low dose of N-methyl glucamine (Glucantime) in Rio de Janeiro, Brazil. *Rev Inst Med Trop Sao Paulo 42*: 321-325.

Oliveira-Neto MP, Schubach A, Araujo ML, Pirmez C 1996. High and low doses of antimony (Sb^{v}) in American cutaneous leishmaniasis. A five years follow-up study of 15 patients. *Memórias do Instituto Oswaldo Cruz 91*: 207-209.

Oliveira-Neto MP, Schubach A, Mattos M, Gonçalves Da Costa SC, Pirmez C 1997a. Intralesional therapy of American cutaneous leishmaniasis with pentavalent antimony in Rio de Janeiro, Brazil - an area of *Leishmania (V.) braziliensis* transmission. *International Journal Dermatology 36*: 463-468.

Oliveira-Neto MP, Schubach A, Mattos M, Goncalves-Costa SC, Pirmez C 1997b. A low dose antimony treatment In 159 patients with American cutaneous leishmaniasis. Extensive follow-up studies (up to 10 years). *American Journal Tropical Medicine Hygiene 57*: 651-655.

Oliveira-Neto MP, Schubach A, Mattos M, Goncalves-Costa SC, Pirmez C 1997c. Treatment of American cutaneous leishmaniasis: a comparison between low dosage (5mg/kg/day) and high dosage (20mg/kg/day) antimony regimens. *Pathologie Biologie 45*: 496-469.

Ooteman M, Vago A, Koury M 2004. Potencial application of low-stringency single primer PCR in the identification of *Leptospira* in the serum of patients with suspected leptospirosis. *Can J Microbiol 50*: 1073-1079.

Osorio Y, Gonzalez SJ, Gama VL, Travi BL 1998. Reinfection in American cutaneous leishmaniasis: evaluation of clinical outcomes in the hamster model. *Mem Inst Oswaldo Cruz 93*: 353-356.

Oster CN, Chulay JD, Hendricks LD, Pamplin CL, 3rd, Ballou WR, Berman JD, Takafuji ET, Tramont EC, Canfield CJ 1985. American cutaneous leishmaniasis: a comparison of three sodium stibogluconate treatment schedules. *American Journal Tropical Medicine Hygiene 34*: 856-860.

Osterberg L, Blaschke T 2005. Adherence to medication. *New England Journal of Medicine 353*: 487-497.

Ouellette M, Papadopoulou B 1993. Mechanisms of drug resistance in *Leishmania*. *Parasitol Today 9*: 150-153.

Pacheco R, Brito C, Sarquis O, Pires M, Borges-Pereira J, Lima M 2005. Genetic heterogeneity in *Trypanosoma cruzi* stains from naturally infected triatomine vectors in Northeastern Brazil: Epidemiological implications. *Biochemical Genetics 43*: 519-530.

Pacheco RS, Brito CM 1999. Reflections on the population dynamics of Trypanosoma cruzi: heterogeneity versus plasticity. *Mem Inst Oswaldo Cruz 94*: 199-201.

Pacheco RS, Fernandes O, Salinas G, Segura I, Momen H, Degrave W, Saravia NG, Campbell DA 2000. Intraspecific heterogeneity in the mini-exon gene localization of Leishmania (Viannia) panamensis and Leishmania (Viannia) guyanensis from Colombia. *J Parasitol 86*: 1250-1253.

Pacheco RS, Lopes UG, Morel CM, Grimaldi Jr. G, Momen H 1986. Schizodeme analysis of *Leishmania* and comparison with some phenotypic techniques. In AJ Rioux, *Leishmania Taxonomie et Phylogenese Application Eco-Epidemiologique*, IMEEE, Montpellier, p. 57-65.

Passos VM, Barreto SM, Romanha AJ, Krettli AU, Volpini AC, Gontijo CM, Falcao AL, Lima-Costa MF 2001. [Cutaneous leishmaniasis in the Metropolitan Region of Belo Horizonte: clinical, laboratorial, therapeutic and prospective aspects]. *Rev Soc Bras Med Trop 34*: 5-12.

Pearson RD, Sousa AQ 1996. Clinical spectrum of Leishmaniasis. *Clin Infect Dis 22*: 1-13.

Pena SDJ, Barreto G, Vago AR, De Marco L, Reinach FC, Dias Neto E, Simpson AJG 1984. Sequence-specific "gene signatures" can be obtained by PCR with single

specific primers at low stringency. *Proceedings National Academy Sciences United States America 91*: 1946-1949.

Pereira SB, Fonseca HHR 1994. Leishmaniose Tegumentar Americana: Epidemiologia e controle. *Revista da Sociedade Brasileira de Medicina Tropical 27*: 45-50.

Pessôa SB, Barretto MP 1948. *Leishmaniose Tegumentar Americana*. Ministério da Educação e Saúde, Serviço de Documentação, Rio de Janeiro, 527 pp.

Prasad LS, Sen S 1996. Migration of Leishmania donovani amastigotes in the cerebrospinal fluid. *Am J Trop Med Hyg 55*: 652-654.

Pupo JA 1946. Estudo clínico de leishmaniose tegumentar americana (*Leishmania braziliensis* - Vianna 1911). *Revista do Hospital das Clínicas 1*: 113-164.

Ramalhinho I 1994. Adesão à terapêutica anti-hipertensiva: contributo para seu estudo. *Faculdade de Ciências Médicas*. Universidade Nova de Lisboa, Lisboa, p. 84.

Rees PH, Keating MI, Kager PA, Hockmeyer WT 1980. Renal clearance of pentavalent antimony (sodium stibogluconate). *Lancet 2*: 226-229.

Ribeiro AL, Drummond JB, Volpini AC, Andrade AC, Passos VM 1999. Electrocardiographic changes during low-dose, short-term therapy of cutaneous leishmaniasis with the pentavalent antimonial meglumine. *Brazilian Journal Medical Biological Research 32*: 297-301.

Ribeiro S, Amado S, Camelier A, Fernandes M, Shenckman S 2000. Estudo caso-controle de indicadores de abandono em doentes com tuberculose. *J Pneumologia 26*

Rioux JA, Lanotte G, Serres E, Pratlong F, Bastien P, Perieres J 1990. Taxonomy of Leishmania. Use of isoenzymes. Suggestions for a new classification. *Ann Parasitol Hum Comp 65*: 111-125.

Roberts WL, Berman JD, Rainey PM 1995. In vitro antileishmanial properties of tri- and pentavalent antimonial preparations. *Antimicrobial Agents Chemotherapy 39*: 1234-1239.

Rocha C, Faggiani F, Schroeter G, Souza A, De Carli G 2006. Adesão à Prescrição Médica em Idosos de Porto Alegre. Revista Ciência & Saúde Coletiva da Associação Brasileira de Pós-Graduação em Saúde Coletiva. Pontifícia Universidade Católica do Rio Grande do Sul. http://www.abrasco.org.br/cienciaesaudecoletiva/artigos/artigo_int.php?id_artigo=488.

Rodgers MR, Popper SJ, Wirth DF 1990. Amplification of kinetoplast DNA as a tool in the detection and diagnosis of Leishmania. *Exp Parasitol 71*: 267-275.

Rodrigues AM, Hueb M, Santos TA, Fontes CJ 2006. [Factors associated with treatment failure of cutaneous leishmaniasis with meglumine antimoniate]. *Rev Soc Bras Med Trop 39*: 139-145.

Rodrigues ML, Costa RS, Souza CS, Foss NT, Roselino AM 1999. Nephrotoxicity attributed to meglumine antimoniate (Glucantime) in the treatment of generalized cutaneous leishmaniasis. *Revista do Instituto de Medicina Tropical de São Paulo 41*: 33-37.

Rodriguez LV, Dedet JP, Paredes V, Mendoza C, Cardenas F 1995. A randomized trial of amphotericin B alone or in combination with itraconazole in the treatment of mucocutaneous leishmaniasis. *Mem Inst Oswaldo Cruz 90*: 525-528.

Rodriguez N, Guzman B, Rodas A, Takiff H, Bloom BR, Convit J 1994. Diagnosis of cutaneous leishmaniasis and species discrimination of parasites by PCR and hybridization. *J Clin Microbiol 32*: 2246-2252.

Rodriguez NM, De Guglielmo Z, Barrios MA, Barrios RM, Zerpa O, Feliciangeli MD 2005. Genetic homogeneity within Leishmania (L.) infantum isolated from human and dogs: the relationship with the sandfly fauna distribution in endemic areas of Nueva Esparta State, Venezuela. *Parasitology 130*: 611-619.

Rojas R, Valderama L, Valderama M, Varona M, Ouellette M, Saravia N 2006. Resistance to antimony and treatment failure in human *Leishmania (Viannia)* infection. *Journal Infectious Diseases 193*: 1375-1383.

Romero GAS, Hueb M, D'Oliveira Jr A, Schubach A 2001a. Simpósio sobre Tratamento das Leishmanioses. *Revista da Sociedade Brasileira de Medicina Tropical 34*: 58-68.

Romero GAS, Schubach A, Oliveira-Neto MP, Hueb M, Dietze R 2001b. Relatório Final do Simpósio: Tratamento das leishmanioses: Existem evidências para recomendar esquemas de tratamento universais na leishmaniose tegumentar? In V Reunião de Pesquisa Aplicada em Leishmanioses, Uberaba.

Rozenfeld S 2003. [Prevalence, associated factors, and misuse of medication in the elderly: a review]. *Cadernos de Saúde Pública 19*: 717-724.

Rozenfeld S, Pepe VLE 1992/93. *Guia Terapêutico Ambulatorial*. Artes Médicas, Porto Alegre, 404 pp.

Russell DG, Wilhelm H 1986. The involvement of the major surface glycoprotein (gp63) of Leishmania promastigotes in attachment to macrophages. *J Immunol 136*: 2613-2620.

Saenz RE, de Rodriguez CG, Johnson CM, Berman JD 1991. Efficacy and toxicity of pentostam against Panamanian mucosal leishmaniasis. *American Journal Tropical Medicine Hygiene 44*: 394-398.

Saiki RB, Scharf S, Fallona F, Mullis KB, Horn GT, Erlich H, Arhein N 1985. Enzimatic amplification of b-globin genomic sequences and restriction site diagnosis of sickle cell anemia. *Science 230*: 1350-1354.

Saldanha AC, Romero GA, Guerra C, Merchan-Hamann E, Macedo VO 2000. [Comparative study between sodium stibogluconate BP 88 and meglumine antimoniate in cutaneous leishmaniasis treatment. II. Biochemical and cardiac toxicity]. *Revista da Sociedade Brasileira de Medicina Tropical 33*: 383-388.

Sampaio RN, Paula CD, Sampaio JH, Furtado RS, Leal PP, Rosa TT, Rodrigues ME, Veiga JP 1997. [The evaluation of the tolerance and nephrotoxicity of pentavalent antimony administered in a dose of 40mg Sb^V/kg/day, 12/12hr, for 30 days in the mucocutaneous form of leishmaniasis. *Revista da Sociedade Brasileira de Medicina Tropical 30*: 457-463.

Sampaio RN, Salaro CP, Resende P, Paula CD 2002. [American cutaneous leishmaniasis associated with HIV/AIDS: report of four clinical cases]. *Rev Soc Bras Med Trop 35*: 651-654.

Sampaio SA, Castro RM, Dillon NL, Martins JE 1971. Treatment of mucocutaneous (American) leishmaniasis with amphotericin B: report of 70 cases. *Int J Dermatol 10*: 179-181.

Santos MA, Marques RC, Farias CA, Vasconcelos DM, Stewart JM, Costa DL, Costa CH 2002. Predictors of an unsatisfactory response to pentavalent antimony in the treatment of American visceral leishmaniasis. *Rev Soc Bras Med Trop 35*: 629-633.

Saravia NG, Weigle K, Segura I, Giannini SH, Pacheco R, Labrada LA, Goncalves A 1990. Recurrent lesions in human *Leishmania braziliensis* infection - reactivation or reinfection? *Lancet 336*: 398-402.

Schubach A 1990. Tese. Estudo da evolução da leishmaniose tegumentar americana em pacientes tratados*. Medicina Tropical*. Instituto Oswaldo Cruz, FIOCRUZ, Rio de Janeiro, p. 141.

Schubach A, Cuzzi-Maya T, Oliveira AV, Sartori A, de Oliveira-Neto MP, Mattos MS, Araujo ML, Souza WJ, Haddad F, Perez Mde A, Pacheco RS, Momen H, Coutinho SG, de Almeida Marzochi MC, Marzochi KB, da Costa SC 2001. Leishmanial antigens in the diagnosis of active lesions and ancient scars of American tegumentary leishmaniasis patients. *Mem Inst Oswaldo Cruz 96*: 987-996.

Schubach A, Haddad F, Oliveira-Neto MP, Degrave W, Pirmez C, Grimaldi G, Jr., Fernandes O 1998a. Detection of *Leishmania* DNA by the polymerase chain reaction in scars of treated human patients. *Journal of Infectious Diseases 178*: 911-914.

Schubach A, Marzochi MC, Cuzzi-Maya T, Oliveira AV, Araújo ML, Oliveira AL, Pacheco RS, Momen H, Conceição-Silva F, Coutinho SG, Marzochi KB 1998b. Cutaneous

scars in American tegumentary leishmaniasis patients: a site of *Leishmania (Viannia) braziliensis* persistence and viability eleven years after antimonial therapy and clinical cure. *American Journal Tropical Medicine Hygiene 58*: 824-827.

Schubach A, Miekeley N, Mortari SR, Moreira JS, Conceição-Silva F, Salgueiro MM, Campos FV, Marzochi KBF, Marzochi MCA 2002. Estudos sobre o metabolismo de antimônio e de suas espécies químicas no tratamento da Leishmaniose Tegumentar Americana com baixas doses de antimônio. *Revista da Sociedade Brasileira de Medicina Tropical 35*: 102-103.

Schubach AO, Marzochi KBF, Moreira JS, Schubach TMP, Araújo ML, Francesconi-do-Vale AC, Passos SRL, Marzochi MCA 2005. Retrospective study of 151 patients with cutaneous leishmaniasis treated with meglumine antimoniate. *Revista da Sociedade Brasileira de Medicina Tropical 38*: 213-217.

Seaton RA, Morrison J, Man I, Watson J, Nathwani D 1999. Out-patient parenteral antimicrobial therapy - a viable option for the management of cutaneous leishmaniasis. *Quarterly Journal Medicine 92*: 659-667.

Sergiev VP, Uzbekov MK, Polevoi NI 1968. [A case of relapse of zoonotic cutaneous leishmaniasis running a course of tuberculoid type]. *Med Parazitol (Mosk) 37*: 331-332.

Sharples CE, Shaw MA, Castes M, Convit J, Blackwell JM 1994. Immune response in healthy volunteers vaccinated with BCG plus killed leishmanial promastigotes: antibody responses to mycobacterial and leishmanial antigens. *Vaccine 12*: 1402-1412.

Sharquie KE 1995. A new intralesional therapy of cutaneous leishmaniasis with hypertonic sodium chloride solution. *J Dermatol 22*: 732-737.

Shaw JJ 1994. Taxonomy of the genus Leishmania: present and future trends and their implications. *Mem Inst Oswaldo Cruz 89*: 471-478.

Shaw JJ, Lainson R, McMahon-Pratt D, David JR 1986. Serodeme of *Leishmania braziliensis* complex. In JA Rioux, *Leishmania Taxonomy and Phylogeny*, IMEEE, Montpellier, p. 179-183.

Silveira FT, Ishikawa EA, De Souza AA, Lainson R 2002. An outbreak of cutaneous leishmaniasis among soldiers in Belem, Para State, Brazil, caused by Leishmania (Viannia) lindenbergi n. sp. A new leishmanial parasite of man in the Amazon region. *Parasite 9*: 43-50.

Silveira FT, Lainson R, Shaw JJ, Garcez LM, Souza AA, Braga RR, Ishikawa EA 1990. [Experimental skin leishmaniasis: II--course of the infection in the Cebus apella primate (Cebidae) caused by Leishmania (V.) braziliensis and L. (L.) amazonensis]. *Rev Soc Bras Med Trop 23*: 5-12.

Silveira L, Ribeiro V 2005. Grupo de adesão ao tratamento: espaço de "ensinagem" para profissionais de saúde e pacientes. *Interface (Botucatu) 9*

Simpson L 1987. The mitochondrial genoma of Kinetoplastid protozoa: genomic organization, transcription, replication and evolution. *Annual Review of Microbiology 41*: 363-382.

SINAN 2003. Leishmaniose Tegumentar - Brasil*. Frequência por UF de residência e faixa etária*. Sistema de Informação e Agravos de Notificação Compulsória, Ministério da Saúde, Brasília.

Soto J, Buffet P, Grogl M, Berman J 1994. Successful treatment of Colombian cutaneous leishmaniasis with four injections of pentamidine. *Am J Trop Med Hyg 50*: 107-111.

Soto-Mancipe J, Grogl M, Berman JD 1993. Evaluation of pentamidine for the treatment of cutaneous leishmaniasis in Colombia. *Clin Infect Dis 16*: 417-425.

Southern EM 1975. Detection of specific sequences among DNA fragments separated by gel electrophoresis. *J Mol Biol 98*: 503-517.

Stamenkovic G, Guduric J, Velickovic Z, Skerl V, Krtolica K, Veljkovic E, Dimitrijevic B 2001. Analysis of 5' non-coding region in hepatitis C virus by single-strand conformation polymorphism and low-stringency single specific primer PCR. *Clin Chem Lab Med 39*: 948-952.

Tallab TM, Bahamdam KA, Mirdad S, Johargi H, Mourad MM, Ibrahim K, el Sherbini AH, Karkashan E, Khare AK, Jamal A 1996. Cutaneous leishmaniasis: schedules for intralesional treatment with sodium stibogluconate. *Int J Dermatol 35*: 594-597.

Teodoro U, Alberton D, Kuhl JB, dos Santos ES, dos Santos DR, dos Santos AR, Oliveira O, Silveira TG, Lonardoni MV 2003. [Ecology of Lutzomyia (Nyssomyia) whitmani in an urban area in Maringa, Parana, Brazil]. *Rev Saude Publica 37*: 651-656.

Thakur CP, Kumar K 1990. Efficacy of prolonged therapy with stibogluconate in post kala-azar dermal leishmaniasis. *Indian Journal Medical Research 91*: 144-148.

Thakur CP, Kumar M, Pandey AK 1991. Comparison of regimes of treatment of antimony-resistant kala-azar patients: a randomized study. *Am J Trop Med Hyg 45*: 435-441.

Thomas-Soccol V, Lanotte G, Rioux JA, Pratlong F, Martini-Dumas A, Serres E 1993. Monophyletic origin of the genus *Leishmania* Ross, 1903. *Annales de Parasitologie Humaine et Comparée 68*: 107-108.

Tibayrenc M, Neubauer K, Barnabe C, Guerrini F, Skarecky D, Ayala FJ 1993. Genetic characterization of six parasitic protozoa: parity between random-primer DNA typing and multilocus enzyme electrophoresis. *Proc Natl Acad Sci U S A 90*: 1335-1339.

Torre-Cisneros J, Prada JL, Villanueva JL, Ververde F, Sanchez-Guijo P 1994. Sucessful treatment of antimony-resistant cutaneous leishmaniasis with liposomal amphotericin B. *Clinical Infectious Diseases 178*: 1024-1025.

Ulrich N 1998. Speciation of antimony(III), antimony(V) and trimethylstiboxide by ion chromatography with inductively coupled plasma atomic spectrometric and mass spectrometric detection. *Anal Chim Acta 359*: 245-253.

Upcroft P 1994. Multiple drug resistance in the pathogenic protozoa. *Acta Trop 56*: 195-212.

Vago A, Andrade L, Leite A, Reis D, Macedo A, Adad S, Tostes Jr S, Moreira M, Brasileiro Filho G, Pena S 2000. Genetic Characterization of *Trypanosoma cruzi* directly from tissue of patients with chronic chagas disease. *Am J Pathol 156*: 1805-1809.

Vago AR, Macedo AM, Oliveira RP, Andrade LO, Chiari E, Galvão LMC, Reis DA, Pereira MES, Simpson AJG, Tostes Jr. S, Pena SDJ 1996. Kinetoplast DNA signatures of *Trypanosoma cruzi* strains obtained directly from infected tissues. *American Journal of Pathology 149*: 2153-2159.

Van Belkum A 1995. Low-stringency single specific primer PCR, DNA sequencing and single-strand conformation polymorphism of PCR products for identification of genetic variants of human papillomavirus type 16. *J Virol Methods 43*: 233-239.

Veiga JP, Wolff ER, Sampaio RN, Marsden PD 1983. Renal tubular dysfunction in patients with mucocutaneous leishmaniasis treated with pentavalent antimonials. *Lancet 2*: 569.

Vermeire E, Hearnshaw H, Van Royen P, Denekens J 2001. Patient adherence to treatment: three decades of research. A comprehensive review. *J Clin Pharm Ther 26*: 331-342.

Victoir K, Banuls AL, Arevalo J, Llanos-Cuentas A, Hamers R, Noel S, De Doncker S, Le Ray D, Tibayrenc M, Dujardin JC 1998. The gp63 gene locus, a target for genetic characterization of Leishmania belonging to subgenus Viannia. *Parasitology 117*: 1-13.

Vieira FA 2003. Desenvolvimento de uma metodologia para determinação de trimetil antimônio, Sb(V) e Sb(III) em amostras clínicas por IC-ICPMS. *Departamento de Química*. Pontifícia Universidade Católica do Rio de Janeiro, Rio de Janeiro, p. 110p.

Walton BC, Chinel LV, Eguia y Eguia O 1973. Onset of espundia after many years of occult infection with *Leishmania braziliensis*. *American Journal Tropical Medicine Hygiene 22*: 696-698.

Weigle KA, Labrada LA, Lozano C, Santrich C, Barker DC 2002. PCR-Based Diagnosis of Acute and Chronic Cutaneous Leishmaniasis Caused by Leishmania (Viannia). *J Clin Microbiol* *40*: 601-606.

Wirth DF, McMahon Pratt D 1982. Rapid identification of Leishmania species by specific hybridization of kinetoplast DNA in cutaneous lesions. *Proc Natl Acad Sci U S A* *79*: 6999-7003.

World Health Organization 1984. The Leishmaniases. World Health Organization.

World Health Organization 1990. Control of Leishmaniases: report of a WHO Expert Committee. World Health Organization, Geneva, p. 1-158.

World Health Organization 2001. Letter to Aventis Pharma Drug Regulatory Affairs, Europe. Essential Drugs and Medicines Policy - W.H.O.

World Health Organization 2003. Adherence a long-term therapies: evidence for action. http://www.who.int/chronic_conditions/en/adherence_report.pdf.

Wyler DJ, Weinbaum FI, Herrod HR 1979. Characterization of in vitro proliferative responses of human lymphocytes to leishmanial antigens. *J Infect Dis* *140*: 215-221.

Yarbuth AL, Anez N, Pena YP, Burguera JL, Burguera A 1994. Antimony determination in tissues and serum of hamsters infected with meglulmine antimoniate. *Annals Tropical Medicine Parasitology* *88*: 37-41.

Zhang X, Cornelis R, Mees L 1998. Speciation of Antimony (III) and Antimony (V) Species by using High Performance Liquid Chromatography coupled to Hydride Generation Atomic Absorption Spectrometry. *J Anal At Spectrom* *13*: 205-207.

Zheng J, Ohata M, Furuta N 2000a. Antimony speciation in environmental samples by using HPLC-ICP-MS. *Analytical Science* *16*: 75-80.

Zheng J, Ohata M, Furuta N 2000b. Studies on the speciation of inorganic and organic antimony compounds in airborne particulate matter by HPLC-ICP-MS. *Analyst* *125*: 1025-1028.

ATTACHMENT

Free and informed consent form

INSTITUTION: Evandro Chagas Clinical Research Institute - Fiocruz

RESEARCH COORDINATOR: Armando de Oliveira Schubach

ADDRESS: Av. Brasil 4365 - Manguinhos - Rio de Janeiro - RJ - CEP 21040-900

TELEPHONES: (0xx21) 3865-9525 / 3865-9541 / FAX (0xx21) 3865-9541

NAME OF THE RESEARCH PROJECT:

Phase III clinical trial for American Cutaneous Leishmaniasis. Equivalence between the standard and alternative scheme with meglumine antimoniate

NAME OF VOLUNTEER: ____

This document seeks to clarify the health problem under study and the research that will be performed, providing information, detailing the procedures and examinations, benefits, drawbacks and potential risks.

American tegumentary leishmaniasis (ATL) is a disease caused by parasites called *Leishmania* and presents as wounds on the skin that is difficult to heal. Sometimes the ATL may become more severe, involving the lining of the nose and throat, even several years after the healing of the wound on the skin. Currently, we cannot quite predict which patient will fall ill again and which will remain permanently cured.

In Brazil, the Ministry of Health (MS) recommends treating patients with ATL with meglumine antimoniate in high doses (20mg per kilogram of body weight per day) for 20 to 30 days, respecting the maximum limit of 3 daily ampoules. However, changes in the

kidneys, heart, liver, pancreas and blood tests are frequent. In addition to joint pain and discomfort at the site of intramuscular injections.

In the IPEC Reference Center on Leishmaniasis, Fiocruz, a low dose of meglumine antimoniate (5 mg per kilogram of body weight per day) has been shown to be effective and well tolerated in the treatment of patients with ATL. Patients with cutaneous form are treated for 30 days. Patients with mucosal form are treated continuously for a minimum of 30 days, preferably without interruption, until mucosal healing, which usually occurs between 30 and 90 days of treatment. Elderly patients or other associated diseases are treated with low doses in series of 10 days at 10 day intervals without medication. Patients who are contraindicated for intramuscular treatment or who show signs of intoxication during treatment may be treated with one or two applications of meglumine antimoniate directly into the skin lesion.

Our accumulated experience suggests that alternative treatment regimens have the same good results as the standard regimen recommended by the Brazilian Ministry of Health, but with fewer adverse effects. However, it is only after the conclusion of this study that we can suggest that the Brazilian Ministry of Health should change recommendations for the treatment of ATL.

Now that your LTA diagnosis has been confirmed, you are being invited to participate in a clinical investigation to be conducted at IPEC-Fiocruz, with the following objectives:

- To evaluate the response to the treatment of ATL with the use of different doses or forms of application of antimonials.
- Describe the behavior of antimonials in the human body according to the different treatment schemes.
- To compare the immunological response of patients treated with different regimens.
- Characterize the isolates of *Leishmania* and check sensitivity to antimonial.

Your participation in this study is entirely voluntary. You may refuse to participate in one or all stages of the research, or even withdraw from it at any time, without this fact causing you any embarrassment or penalty on the part of the Institution. Your medical care will not be affected if you decide not to participate or if you decide to leave the study already started. Your doctors may also interrupt your participation at any time if they deem it convenient for your health.

Their participation in relation to the project is to authorize the indication of their treatment for cutaneous form of ATL with meglumine antimoniate intramuscularly, be done by lot

for one of the following groups: 1) high dose for 20 continuous days; 2) low dose for 30 continuous days. If there is any contraindication for you to receive any of these regimens or intolerance in need of interrupting a started regimen, treatment will be performed with one or two medication applications, with a two-week interval, directly on the skin lesion. In the case of a mucosal form you can be drawn to one of the following groups: 1) high dose for 30 days; 2) low dose daily until curing. If there is any intolerance with the need to interrupt one of the initiated regimens, the treatment will be performed at low dose in series of 10 days with intervals of rest, then until the cure. Doctors who will evaluate your treatment will not know which regimen is used and you will not know if you are being treated at high or low dose so as not to be influenced in the trial.

Your authorization will also be required: 1) for the use of photographic documentation or filming of your lesions for study; 2) so that part of the material collected periodically to perform examinations to monitor the evolution of their disease, as well as the results of these routine exams and their treatment are used in this study; 3) so that part of the collected samples are stored in order to serve other studies aimed at better understanding the disease, the development and evaluation of new diagnostic methods; evaluation of treatment response; and so on, provided that such study is previously analyzed and authorized by a Research Ethics Committee.

By participating in this study, you will have some responsibilities: follow your doctor's instructions; attend the health facility on the scheduled dates; and report to your physician all reactions you present during treatment, both positive and negative. The examinations and procedures applied will be free of charge. You will receive all appropriate medical care for your illness. If you need medical care, during the period you are participating in the study, even outside your appointment, contact the Evandro Chagas Clinical Research Institute - Fiocruz. In case of need, call Dr. Armando de Oliveira Schubach, Dr. Cláudia Maria Valete Rosalino or Dr. Maria Inês Pimentel in the above telephones. If you have any problem that requires hospitalization, the medical team will provide your bed at the Evandro Chagas Clinical Research Institute - Fiocruz.

Your identity will be kept confidential. The results of the study may be published without revealing your identity and your images may be released as long as you cannot be recognized. However, if necessary, your medical records will be available for consultation to the team involved in the study, to the Research Ethics Committee, to the Health Authorities and to you.

You can and should ask any questions you deem necessary before agreeing to participate in the study, as well as at any time during treatment. Your doctor should

provide all necessary information regarding your health, your rights, and any risks and benefits related to your participation in this study.

Disadvantages and main risks known to date: meglumine antimony agent usually causes undesirable effects, should not be used during pregnancy and its use in women of reproductive age should be accompanied by the use of an effective contraceptive method as a male or female latex condom ("Condom"), female diaphragm or oral contraceptive ("pill").

Forms of reimbursement: When necessary, on the days of their attendance, food may be provided according to routine of the Nutrition and Social Service of IPEC for outpatients.

Expected benefits: You are expected to be cured of the ATL at the end of treatment, although return visits for several years after treatment are necessary to confirm cure. The results of this study may not benefit you directly, but, in the future, may benefit others, as this study is expected to contribute to a better monitoring of treatment of patients with ATL, which may be done more effectively and safely.

I declare that I have read and understood all the information regarding this study and that all my questions have been adequately answered by the medical staff, who will be on hand to answer my questions whenever I have questions.

I have received a copy of this consent form and hereby consented voluntarily to participate in this research study.

____ ____________

Patient Name: Date

____ ____________

Medical Name: Date

ANNEX (TERM OF CONSENT FOR DIAGNOSTIC PROCEDURES)

Free and informed consent form

INSTITUTION: Evandro Chagas Clinical Research Institute - Fiocruz

RESEARCH COORDINATOR: Armando de Oliveira Schubach

ADDRESS: Av. Brasil 4365 - Manguinhos - Rio de Janeiro - RJ - CEP 21040-900

TELEPHONES: (0xx21) 3865-9525 / 3865-9541 / FAX (0xx21) 3865-9541

NAME OF THE RESEARCH PROJECT:

Study for the systematization of the care of patients with American Cutaneous Leishmaniasis in the Reference Center in ATL - Evandro Chagas Clinical Research Institute - Fiocruz

NAME OF VOLUNTEER: ____

American tegumentary leishmaniasis (ATL) is a disease that strikes humans and animals, including the dog, caused by parasites called *Leishmania*. The disease is transmitted by the "straw mosquito", which lives in areas of forest, banana plantations, mango etc and are located near human dwellings, where they usually enter to feed on the blood of people and domestic animals. The ATL presents as hard-to-heal wounds on the skin. Sometimes the ATL may become more severe, involving the lining of the nose and throat, even several years after the healing of the wound on the skin. Currently, we cannot quite predict which patient will fall ill again and which will remain permanently cured.

Other diseases such as bacterial infections, tuberculosis, syphilis, sporotrichosis, other mycoses, tumors etc can manifest in a manner similar to leishmaniasis and need to be differentiated in order to initiate the correct treatment. However, with the exams currently available, you cannot always be absolutely certain about the disease in question.

At the moment, several questions need to be answered like: In what other ways can ATL manifest itself? How do laboratory tests behave before, during and after treatment? Which patients, even after treatment, will reopen their scars or develop disease inside the nose or throat? Which other similar diseases are being confused with the ATL and which exams should be used for clarification? What is the role of humans as reservoirs

of disease? What are the best forms of treatment? What steps should be taken to control the problem?

You are being invited to participate in a clinical investigation to be held at IPEC-Fiocruz, with the following objectives:

- Describe aspects of ATL: clinical manifestations and laboratory tests, trying to establish patterns of presentation of the disease and its mode of evolution, comparing with other diseases.
- To evaluate the use of antimonials and other drugs used in the treatment of ATL, taking into consideration treatment time, toxicity, ease of administration, cost and absence of involvement of the mucous membranes of the nose and throat.
- Isolate, identify and compare ATL-causing *Leishmania* parasites from several localities.

This document seeks to clarify the health problem under study and the research that will be performed, providing information, detailing the procedures and examinations, benefits, drawbacks and potential risks.

Your participation in this study is voluntary. You may refuse to participate in one or all stages of the research, or even withdraw from it at any time, without this fact causing you any embarrassment or penalty on the part of the Institution. Your medical care will not be affected if you decide not to participate or if you decide to leave the study already started. Your doctors may also interrupt your participation at any time if they deem it convenient for your health.

Your participation in relation to the Project is to authorize a series of tests to diagnose our illness, and part of this material, as well as the results of these routine exams, are used in this study. Your authorization will also be required: 1) for the use of photographic documentation or filming of your lesions for study, 2) so that part of the material collected periodically to perform tests to monitor the evolution of your disease, as well as the results of these routine tests and its treatment are used in this study, 3) so that part of the collected samples is stored in order to serve other studies aimed at better understanding the disease, the development and evaluation of new diagnostic methods; evaluation of response to treatment etc., provided that such study is previously analyzed and authorized by a Research Ethics Committee.

The examinations and any procedures applied will be free of charge. You will receive all appropriate medical care for your illness.

By participating in this study you will have some responsibilities: strictly follow your doctor's instructions; attend the health facility on the scheduled dates; tell your doctor all the reactions you present during treatment, both positive and negative. If you need medical attention, during the period in which you are participating in the study, go to the Evandro Chagas Clinical Research Institute - Fiocruz, even outside your appointment day. In case of need, please call Dr. Armando de Oliveira Schubach, Dr. Fátima Conceição-Silva or Dr. Mariza Salgueiro on the above telephones. If you present any clinical condition that requires hospitalization, the medical team will provide your hospital bed at the Evandro Chagas - Fiocruz Clinical Research Institute. Your animals with suspected LTA may be treated free of charge by veterinarian Dr. Tânia Maria Valente Pacheco at the IPEC Zoonoses Service.

Your identity will be kept confidential. The results of the study may be published without revealing your identity and your images may be released as long as you cannot be recognized. However, if necessary, your medical records will be available for consultation to the team involved in the study, to the Research Ethics Committee, to the Health Authorities and to you.

You can and should ask any questions you deem necessary before agreeing to participate in the study, as well as at any time during treatment. Your doctor should provide all necessary information regarding your health, your rights, and any risks and benefits related to your participation in this study.

Procedures, exams and tests to be used:

Before treatment there will be collection of information about the disease; general medical examination and examination of the skin with description and photographic documentation or filming of the lesions; Internal examination of the nose and throat with a device called optical fiber, which allows viewing of small lesions or difficult to reach sites for description and photographic documentation or filming of the lesions (local anesthetic spray if necessary). Removal with local anesthesia of a small fragment of the skin, mucosal or "inguinal" lesion to perform diagnostic tests (microscopic appearance of diseased tissue and cultures to try to isolate possible disease agents such as fungi, bacteria and *Leishmania* parasites) and for research (identification of cells and other components of the inflammatory response, as well as new methods for identifying possible agents of the disease). Other materials may also be collected in an attempt to isolate the causative agent: syringe and needle aspiration from the edge of the lesion and secretions from closed skin lesions.

Other tests will also be done to diagnose other diseases that may be confused with ATL, to classify disease severity, and to evaluate the effects of medications to be used during your treatment: one to four skin tests (one-tenth of a milliliter reaction for a certain disease in the skin of the anterior region of the forearm, which should be reviewed between 2 and 3 days after the injection); Blood samples (equivalent to approximately three tablespoons), saliva (collected with a type of swab), radiography of the lungs and face (if necessary supplemented by computed tomography); and electrocardiogram.

The treatment of ATL in human patients is usually with intramuscular (IM), intravenous (IV) meglumine antimonia agent an injection a day, usually for a continuous period of 30 days or with rest intervals. Exceptionally, for the elderly, patients with severe diseases or who do not tolerate normal treatment, the intralesional route (IL) may be used. Treatment time may be shortened or increased as needed. Other treatment options are amphotericin B (IV) and pentamidine (IM), both injectables and requiring follow-up measures similar to those of meglumine antimoniate.

After initiation of treatment, you should attend approximately three appointments within 10, 20, and 30 days. If the lesions do not heal completely, treatment may be continued for as long as necessary. Upon clinical cure, you should return for reassessment at 1, 3, 6, 9, and 12 months after the end of treatment. And thereafter, at least once a year for an indefinite period (at least 5 years).

Medical evaluation and blood tests should be performed at each evaluation day (in the approximate amount of one or two tablespoons) to evaluate the effects of the medicines used in their treatment and / or to evaluate the evolution of the disease. Other tests, such as the electrocardiogram during treatment, may be performed when indicated.

Main drawbacks and risks known to date:

The collection of blood may cause some pain at the time of venipuncture and, eventually, there may be a purple area on the site, which will return to normal within a few days.

Occasionally, skin tests may show a strong reaction with local inflammation, blistering, and, more rarely, wound formation. The whole process usually recedes within a few days to a few weeks.

Both skin tests and the anesthetic injected at the time of biopsy (removal of a small piece of skin for examination) may cause allergy, usually limited to the appearance of red, itchy, itchy areas on the skin and respond well to anti-allergic drugs. More rarely, there may be a more severe reaction to breathing and the need for a more intensive care in IPEC.

At the site of the biopsy, inflammation and pain may occur, with or without bacterial infection. If this occurs, you may need to use pain medications and antibiotics. Medications such as meglumine antimoniate and pentamidine usually cause undesirable effects, should not be used in pregnancy and their use in women of reproductive age should be accompanied by the use of effective contraceptive method as a condom for male or female latex (female condom), female diaphragm or oral contraceptive ("pill"). When treatment cannot be delayed, amphotericin B may be used during pregnancy. X-rays should also not be performed on pregnant women.
Forms of compensation:

When necessary, on the days of its attendance, food may be provided according to routine of the Nutrition and Social Service of IPEC for outpatients.
Expected benefits:

It is expected that at the end of the treatment you will be cured of ATL, although return visits for several years after treatment are necessary to confirm cure. The results of this study may or may not benefit you directly, but in the future, it may benefit others, as it is expected that this study will contribute to a better diagnosis and follow-up of patients with ACL, and may provide a basis for the treatment to be done more effectively and safely.

If your research demonstrates a different diagnosis than ACL, you will be properly advised to seek the most appropriate treatment for your case.
I declare that I have read and understood all the information regarding this study and that all my questions have been adequately answered by the medical staff, who will be on hand to answer my questions whenever I have questions.
I have received a copy of this consent form and hereby consented voluntarily to participate in this research study.

____________________________________ ____________

Patient Name: Date

____________________________________ ____________

Medical Name: Date

Witness[1] Name: Date

Witness name[2]: Date

Research Ethics Committee (REC)

CONSOLIDATED AUDIT OPINION

Protocol 0055.0.009.000-07

1. Identification:

Project Title: "Phase III clinical trial for American tegumentary leishmaniasis. Equivalence between the standard and alternative scheme with meglumine antimoniate ".

Subproject A: "Controlled, randomized, double-blind, and phase III clinical trial to verify the effectiveness and compare the safety between standard and alternative dose regimens of meglumine antimoniate in the treatment of cutaneous leishmaniasis"

Researcher in charge: Armando de Oliveira Schubach.

Institution in charge: Evandro Chagas Institute of Clinical Research / Fiocruz.

Date Submitted to the REC: 25/09/2007.

In addition to the Consolidated Audit Opinion dated from October 17th, 2007, we would like to inform that in 2008 there were additional information about some changes related to the Subproject A:

1) Modification of the clinical trial of equivalence to a non-inferiority trial with a non-inferiority margin of 15%.
2) Number of patients needed to be included in this subproject will be 72 patients in total.
3) Patients will be recruited in two groups of treatment: 20 mg Sb5+/kg/day for 20 days and 5 mg Sb5+/kg/day for 30 days.

In this sense, we would like to clarify that on the date of the first Consolidated Audit Opinion, this addition was not previously and clearly informed.

For this reason, we would like to request that from this date onwards that the new information should be incorporated into the final modification of the aforementioned Consolidated Audit Opinion.

The Consolidated Audit Opinion of 2008 has the following connotation:

Signature of coordinator:

Dra. Lea Ferreira Camillo-Coura

Research Ethics Committee Coordinator

IPEC – FIOCRUZ

Research Ethics Committee (REC)

CONSOLIDATED AUDIT OPINION

Protocol 0055.0.009.000-07

1. Identification:

Project Title: "Phase III clinical trial for American tegumentary leishmaniasis. Equivalence between the standard and alternative scheme with meglumine antimoniate ".

Subproject A: "Controlled, randomized, double-blind, and phase III clinical trial to verify the effectiveness and compare the safety between standard and alternative dose regimens of meglumine antimoniate in the treatment of cutaneous leishmaniasis"

Researcher in charge: Armando de Oliveira Schubach.

Institution in charge: Evandro Chagas Institute of Clinical Research / Fiocruz.

Date Submitted to the REC: 25/09/2007.

2. Summary:

This constitutes an amendment to Subproject A of the main project, with modification of the equivalence study for a non-inferiority study with a margin of 15%.

This subproject is a phase III randomized controlled clinical trial in 72 patients with cutaneous Leishmaniasis (LC) treated at the Reference Center on Leishmaniasis - IPEC / Fiocruz. Eligible individuals who agree to participate will be allocated randomly in one of two treatment groups: 20 mg Sb5 + / kg / day for 20 days and 5 mg Sb5 + / kg / day for 30 days. It has as main objective to compare the schemes with meglumine antimoniate recommended in Brazil for American Tegumentary Leishmaniasis (ACL) with the alternative scheme. Specific objectives: to compare effectiveness with a 15% non-inferiority margin and safety between the groups in the treatment of cutaneous leishmaniasis (CL). The main potential benefit of this trial is the possibility of subsidizing the use of lower, potentially less toxic and lower cost antimony doses for the treatment of cutaneous leishmaniasis, including elderly patients with comorbidities (heart, kidney and liver diseases).

3. General Comments: (In compliance with Resolution CNS 196/96).

The research project has an adequate design plan. The term of free and informed consent was prepared in a language accessible to the research subject. This project is partially funded with resources approved by edict MCT / CNPq / MS-SCTIE-DECIT 25/2006.

4. Diligences:

Yes. They were satisfied.

5. Opinion: APPROVED.

Date: September 1st, 2008

Signature of coordinator:

Dr. Lea Ferreira Camillo-Coura

Research Ethics Committee Coordinator

IPEC - FIOCRUZ
