# Supplementary material for: Low versus high dose of antimony for American cutaneous leishmaniasis: A randomized controlled blind non-inferiority trial in Rio de Janeiro, Brazil
Source: PLoS One. 2017 May 30;12(5):e0178592. doi: 10.1371/journal.pone.0178592 (PMC5448803; doi:10.1371/journal.pone.0178592)
Supplement: S6 Appendix — (PDF) [file pone.0178592.s006.pdf]

Ministério da Saúde

FIOCRUZ
Fundação Oswaldo Cruz
INSTITUTO DE PESQUISA CLÍNICA EVANDRO CHAGAS

IPEC

ENSAIO CLÍNICO FASE III PARA LEISHMANIOSE TEGUMENTAR AMERICANA. EQUIVALÊNCIA ENTRE O ESQUEMA PADRÃO E ALTERNATIVOS COM ANTIMONIATO DE MEGLUMINA

Subprojetos

A. **Ensaio clínico controlado, randomizado, duplo-cego e de fase III para verificar a equivalência da efetividade e comparar a segurança entre o esquema padrão e alternativo com antimoniato de meglumina no tratamento da leishmaniose cutânea**
B. **Desenvolvimento e aplicação de metodologias para análise de especiação de antimônio em pacientes com leishmaniose tratados com antimoniato de meglumina**
C. **Comparação da resposta imune anti-*Leishmania* de pacientes com leishmaniose tegumentar americana tratados com o esquema padrão ou alternativos com antimoniato de meglumina**
D. **Comparação da resposta imune celular *in vitro* a antígenos de referência e de parasitos isolados dos respectivos pacientes com leishmaniose tegumentar americana que evoluiram para cura ou reativação das lesões após terapia antimonial**
E. **Avaliação da variabilidade genética e da sensibilidade ao antimonial *in vitro* de amostras de *Leishmania (V.) braziliensis* isoladas de pacientes antes e após o tratamento com o esquema padrão ou alternativos com antimoniato de meglumina**
F. **Estudo cego para avaliação da efetividade e segurança do antimoniato de meglumina intralesional em pacientes com leishmaniose cutânea e contra-indicação de terapia sistêmica**

G. Estudo cego para avaliação da efetividade e segurança do antimoniato de meglumina intralesional no tratamento de pacientes com leishmaniose cutânea excluídos do subprojeto A (tratamento sistêmico com antimoniato de meglumina)

H. Ensaio clínico fase III para leishmaniose mucocutânea ou mucosa. Comparação entre o esquema padrão e alternativo com antimoniato de meglumina

I. Estudo cego para avaliação da efetividade e segurança do antimoniato de meglumina em dose baixa intermitente no tratamento de pacientes com leishmaniose mucosa excluídos do subprojeto H (esquema padrão ou dose baixa contínua)

J. Avaliação da adesão dos pacientes envolvidos no Ensaio Clínico fase III com o esquema padrão e alternativo com antimoniato de meglumina no tratamento da leishmaniose tegumentar americana

K. Estudo clínico-molecular na leishmaniose mucosa: diagnóstico e rastreamento de subpopulações de *Leishmania (Viannia) braziliensis*

Instituições Participantes

FUNDAÇÃO OSWALDO CRUZ (FIOCRUZ)
Av. Brasil, 4365- Manguinhos, Rio de Janeiro
CEP: 21.040-900 Tel: (xx 21) 3865-8235

Instituto de Pesquisa Clínica Evandro Chagas (IPEC)
Av. Brasil, 4365 Manguinhos, Rio de Janeiro
CEP: 21040-900 Tel: (21) 3865-9595 Fax: (21) 3865-9541

1. Serviço de Infectologia
2. Centro de Referência em Leishmanioses
3. Departamento de Epidemiologia
4. Serviço de Zoonoses
5. Serviço de Patologia
6. Serviço de Parasitologia
7. Serviço de Especialidades Clínicas
8. Serviço de Farmacocinética
9. Serviço de Farmácia
10. Serviço de Patologia Clínica

Instituto Oswaldo Cruz (IOC)
Av. Brasil, 4365 Manguinhos, Rio de Janeiro
CEP: 21040-900 Tel: (21) 3865-9595 Fax: (21) 3865-9541

11. Laboratório de Imunoparasitologia
12. Laboratório de Bioquímica de Tripanosomatídeos
13. Laboratório de Sistemática Bioquímica

PONTIFÍCIA UNIVERSIDADE CATÓLICA DO RIO DE JANEIRO (PUC)
Av. Brasil, 4365 Manguinhos, Rio de Janeiro
CEP: 21040-900 Tel: (21) 3865-9595 Fax: (21) 3865-9541

14. Departamento de Química

SUBPROJETO A

Ensaio clínico controlado, randomizado, duplo-cego e de fase III para verificar a equivalência da efetividade e comparar a segurança entre o esquema padrão e alternativo com antimoniato de meglumina no tratamento da leishmaniose cutânea

A 5. Hipóteses a serem testadas

A 5.1 Não-inferioridade de efetividade

H_{0E} = **Não** existe não-inferioridade entre o esquema atualmente recomendado no Brasil para o tratamento da leishmaniose cutânea (20 mg Sb^{5+}/kg/dia por 20 dias) e o esquema alternativo com 5 mg por 30 dias, isto é o esquema alternativo **não** é não-inferior ao padrão.

H_{1E} = o esquema alternativo com 5 mg é não-inferior ao atualmente recomendado com 20 mg.

A 5.2 Segurança

H_{0S} = Existe equivalência de toxicidade entre o esquema atualmente recomendado no Brasil para o tratamento da leishmaniose cutânea (20mg Sb^{5+}/kg/dia por 20) e o esquema alternativo com 5mg por 30 dias, isto é não existe diferença de toxicidade entre os esquemas

H_{1S} = o esquema atualmente recomendado com 20 mg é mais tóxico que o esquema alternativo

Entretanto, independente do esquema terapêutico, espera-se que as lesões localizadas acima dos joelhos encontrem-se epitelizadas por ocasião do término do tratamento, enquanto a epitelização das lesões localizadas na pernas e pés ocorra, com mais frequência, após o término do período de administração dos medicamentos, particularmente naqueles casos com insuficiência vascular associada. Também é esperado que, independente do esquema terapêutico, pacientes acima de 50 anos apresentem efeitos adversos com maior frequência e maior intensidade que pacientes mais jovens.

A 6. Objetivos

A 6.1 Objetivo geral

Comparar a efetividade e a segurança do antimoniato de meglumina na dose de 20 mg Sb^{5+}/kg/dia por 20 dias ou com 5 mg por 30 dias, no tratamento de pacientes com leishmaniose cutânea

A 6.2 Objetivos específicos

1. Comparar a efetividade imediata (cura inicial) e em um ano após o tratamento (cura definitiva), de um grupo de tratamento antimonial para leishmaniose cutânea alternativo com o esquema padrão preconizado pelo Ministério da Saúde, com uma margem de não-inferioridade de 15%.
2. Comparar a a frequência e gravidade de efeitos adversos clínicos, laboratoriais e eletrocardiográficos entre os diferentes grupos de tratamento antimonial.
3. Comparar a frequência e gravidade de efeitos adversos e a efetividade entre grupos conforme idade, gênero e raça.
4. Comparar as frequências de epitelização alcançadas nos dias 20, 30 e 50 de tratamento em um mesmo paciente.
5. Comparar o tempo em dias até a epitelização das lesões segundo a localização acima e abaixo dos joelhos, entre os esquemas de tratamento antimonial padrão e alternativo, controlando para a concomitância com insuficiência vascular associada.

A 7. Sujeitos e métodos

A 7.1 Delineamento do estudo

Ensaio clínico controlado com o tratamento padrão, randomizado, duplo-cego e de fase III.

A 7.2 Descrição da medicação e esquema de intervenções

No Brasil, o antimoniato de meglumina (Aventis, São Paulo, Brasil) é estocado em temperatura ambiente e distribuído para a rede de saúde pela Secretaria de Vigilância em Saúde - SVS/MS, a qual fornecerá um único lote para ser utilizado em todos os pacientes.

Este medicamento é de aplicação intramuscular (deltóide ou glúteos) em dose única diária. Serão empregadas seringas descartáveis, de plástico, esterilizadas, de 5 a 20 mL e agulhas descartáveis, esterilizadas, de 25 X 7 mm.

O estudo com a utilização da via intramuscular (I.M.) sem supervisão direta permitirá a avaliação do uso nas condições reais (efetividade) empregadas pelos serviços de saúde no estado do Rio de Janeiro, pois embora, seja possível a prescrição por via intravenosa, não é prática usual devido à difícil exeqüibilidade em nível ambulatorial.

Cada paciente será incluído em um dos seguintes grupos de tratamento com antimoniato de meglumina por via IM:

1. 20mg Sb^{5+}/kg/dia por 20 dias
2. 5mg Sb^{5+}/kg/dia por 30 dias

Não haverá "cross-over" entre os grupos para efeitos deste estudo. Os dados daqueles pacientes que necessitarem interrupção definitiva de um esquema serão analisados segundo o grupo ao qual foram randomizados, isto é, pela intenção de tratar. A coleta de dados se dará conforme o cronograma de consultas.

A 7.3 Plano de amostragem

A 7.3.1 Tamanho amostral

Optou-se por constituir os grupos com o tamanho amostral mínimo necessário (36 pacientes) para responder a todos os desfechos de interesse.

Acreditamos que a comparação da efetividade entre os dois esquemas revele resultados não inferiores para o esquema alternativo para os seguintes desfechos:

1. frequência de boa resposta inicial (avaliada nos dias 20, 30 e 50)
2. tempo (em dias) até alcançar a epitelização de todas as lesões
3. tempo (em dias) para alcançar a cicatrização total de todas as lesões
4. frequência de boa resposta tardia (1 ano de seguimento ambulatorial conforme cronograma)
5. frequência de reativação após o tratamento (até 2 anos de seguimento ambulatorial conforme cronograma)

Utilizou-se o nível de significância de 5% e poder de 80% para calcular o tamanho necessário das amostras para se comparar as frequências dos desfechos de interesse no grupo padrão com o outro grupo. Para testar a não-inferioridade entre as proporções nos desfechos primários de efetividade, considerou-se um limite aceitável de 15% de diferença entre essas proporções de cicatrização. Para isso serão necessários 36 pacientes em cada grupo de esquema terapêutico.

Prevendo-se a utilização do teste pareado de McNemar, para comparar as proporções de cicatrização ou epitelização nos dias 20, 30 e 50 serão necessários no mínimo 36 pacientes em cada grupo (conforme assumirmos uma diferença clinicamente irrelevante de 30%).

Para comparar a efetividade de tratamento segundo a localização das lesões, acima ou abaixo do joelho, serão necessários somente 12 pacientes em cada grupo de tratamento.

Espera-se que o grupo alternativo (baixa dose, 5 mg/kg) apresente resultados não-inferiores com uma margem de 15%, e com uma frequência menor de efeitos adversos clínicos, além de uma baixa frequência de efeitos adversos laboratoriais e eletrocardiográficos. Entretanto, espera-se encontrar uma diferença expressiva nos

seguintes desfechos, quando compararmos a segurança do tratamento padrão de 20mg com o esquema alternativo:

1. frequência de efeitos adversos clínicos, laboratoriais ou eletrocardiográficos em qualquer grau de intensidade
2. frequência de efeitos adversos clínicos, laboratoriais ou eletrocardiográficos de maior intensidade
3. frequência de interrupção do tratamento provocada por Eventos Adversos.
4. frequência de abandonos de tratamento

No caso de pacientes idosos, espera-se encontrar uma acentuada elevação na frequência e na gravidade de EA no grupo 1 (20mg) quando comparado ao grupo 2 (5mg).

Considerou-se o nível de significância de 5% e o poder de 80% para calcular o tamanho das amostras necessário para se comparar o grupo padrão com o outro grupo.

A 7.3.2 Estratégia alocação (randomização)

Os indivíduos elegíveis (ver critérios de elegibilidade) e que concordarem em participar (firmando o termo de consentimento livre e esclarecido) serão alocados aleatoriamente em um dos grupos de tratamento, conforme ordem de chegada, até o preenchimento total dos grupos. A lista de alocação aleatória (numerada) será construída em EPI-INFO 6.4 com o número total de sujeitos necessários para a pesquisa e disponibilizada na farmácia do IPEC. Haverá estratificação com análise de interação para os aspectos clínico-epidemiológicos que podem ter um efeito modificador sobre os desfechos (idade, sexo e raça) e blocagem com blocos pré-definidos de 12 a fim de garantir o equilíbrio entre os grupos de tratamento a qualquer momento em caso de necessidade de interrupção da pesquisa.

A 7.4 Critérios de elegibilidade

A 7.4.1 Critérios de inclusão

1. Leishmaniose cutânea com diagnóstico parasitológico por um ou mais dos seguintes métodos: exame direto (raspado ou *imprint*), histopatológico, cultura, imunohistoquímica ou PCR
2. história de exposição em área endêmica do estado do Rio de Janeiro
3. ausência de tratamento anterior com antimoniato de meglumina

A 7.4.2 Critérios de exclusão

1. mulheres que não fazem uso de métodos contraceptivos ou o fazem de forma inadequada
2. gestantes
3. menores de 13 anos

4. tratamento prévio com antimoniato de meglumina
5. uso de terapia imunossupressora (corticoterapia, quimioterapia para câncer) ou uso de medicações para tuberculose ou hanseníase.
6. presença de alterações basais clínicas equivalentes a efeito adverso nível $\geq$G3
7. presença de alterações basais laboratoriais equivalentes a efeito adverso nível $\geq$G2
8. presença de alterações basais eletrocardiográficas equivalentes a efeito adverso nível $\geq$G4 e/ou QTc basal $\geq$0,46ms (equivalente a EA nível G1)

A 7.5 Pacientes do estudo e cronograma para inclusão

Serão incluídos no estudo 72 pacientes com LC provenientes do Estado do Rio de Janeiro, atendidos no Centro de Referência em Leishmanioses - IPEC - Fiocruz.

Como as lesões localizadas abaixo do joelho geralmente só estarão totalmente epitelizadas várias semanas após o término do tratamento, poderia ocorrer viés na avaliação da efetividade de tratamento caso pacientes com esta localização de lesão estivessem presentes com maior frequência em determinado grupo de tratamento. Do mesmo modo pacientes com idade acima de 50 anos costumam apresentar efeitos adversos com mais frequência e maior intensidade, podendo influenciar negativamente na avaliação da segurança, caso estivessem presentes com maior frequência em determinado grupo. Esses vieses de inclusão serão evitados mediante a estratégia de randomização em blocos e controlados com análise multivariada, considerando as seguintes variáveis como confundidoras potenciais: idade acima e abaixo de 50 anos e presença de lesões acima e abaixo dos joelhos.

A 7.6 Desfechos

A 7.6.1 Desfechos de Efetividade: definição

1. Resposta terapêutica inicial - presença ou ausência de epitelização total de todas as lesões até a consulta do dia 110 (cura inicial).
2. Resposta terapêutica tardia - presença ou ausência dos seguintes elementos na progressão esperada para cicatrização total:
    - desaparecimento de crostas até a consulta do dia 140.
    - desaparecimento de descamação (superfície lisa) até a consulta do dia 230.
    - desaparecimento de infiltração até a consulta do dia 320.
    - desaparecimento de eritema até a consulta do dia 360 (cura definitiva).
    - não surgimento de lesão mucosa até a consulta do dia 770.
    - reaparecimento de qualquer estágio anterior ao alcançado, mantida em 2 observações realizadas com intervalo de pelo menos duas semanas.

A 7.6.2 Desfechos de segurança (eventos adversos): definição, intensidade e relação com a droga do estudo

Considera-se evento adverso (EA) qualquer intercorrência, desfavorável ou inesperada, constatada pelo pesquisador ou relatada pelo paciente, com início durante o uso do medicamento ou até 30 dias após a suspensão deste. A averiguação de EA se fará por rememoração espontânea e questionada pelo médico segundo uma ficha padronizada nos dias 10, 20, 30, 50, 60 e 80.

A classificação da gravidade de eventos adversos (clínicos, laboratoriais e eletrocardiográficos) se dará segundo as tabelas nos **anexos 1 e 2**, adaptadas da "AIDS Table for Grading Severity of Adult Adverse Experiences, 1992" (Adult AIDS Clinical Trials Group August, 1992).

A relação causal com a droga do estudo (= efeito adverso) será avaliada pelo pesquisador e classificada da seguinte forma:

1. Definitivo (Altamente Provável): Uma reação que ocorre numa seqüência de tempo razoável após a administração da droga ou quando os níveis da droga se estabeleceram nos fluídos corporais e nos tecidos; que segue uma resposta padrão conhecida da droga suspeita; que é confirmada pela melhora após parada da droga e reaparece na exposição repetida.
2. Provável: Uma reação que ocorre numa seqüência de tempo razoável após a administração da droga; que segue uma resposta padrão conhecida da droga suspeita; que é confirmada pela melhora após parada da droga e que não pode ser

razoavelmente explicada pelas características conhecidas do estado clínico do indivíduo.

3. Possível: Uma reação que ocorre numa seqüência de tempo razoável após a administração da droga; que segue uma resposta padrão conhecida da droga suspeita mas que pode ser produzida pelas características do estado clínico do indivíduo ou outros modos de terapia administrada ao indivíduo.
4. Remota (Provavelmente Não): Uma reação que ocorre numa seqüência de tempo razoável após a administração da droga; que segue uma resposta padrão conhecida da droga suspeita mas que pode ser razoavelmente explicada pelas características do estado clínico do indivíduo.
5. Definitivamente Não: Qualquer reação que não preencha os critérios acima.

A 7.7 Medicações permitidas durante o ensaio

Não haverá restrições de uso de medicações sintomáticas e para outras doenças com exceção das listadas nos critérios de exclusão (tuberculostáticos, imunossupressor e quimioterapia para câncer).

A 7.8 Manuseio de efeitos adversos

Os EA serão anotados em formulário adequado, devendo constar: a descrição do efeito adverso, intensidade, relação com a droga investigada, data do início, data do término, duração e conduta tomada.

Como regra geral, poderão ser tomadas as condutas adequadas a cada EA.

A 7.9 Parâmetros de acompanhamento

Os parâmetros (desfechos) de efetividade e segurança serão monitorados segundo cronograma de execução (item A 7.7).

Os vieses de aferição serão minimizados através da adoção de uma ficha padronizada para coleta de dados a ser preenchida a cada consulta pela equipe de profissionais treinados. Nessa ficha constarão dados de adesão ao protocolo, informações sobre periodicidade da administração correta (ou não) da droga, a coleta de amostras biológicas para exames e a ocorrência de efeitos adversos e dos desfechos de interesse.

A 7.10 Monitoramento da adesão

As perdas de seguimento tentarão ser contornadas / minimizadas mediante busca ativa: através de telefonema (dois) e telegrama (um), caso não seja obtida resposta anterior. Esses recursos serão oferecidos a todos os pacientes que faltarem uma consulta agendada.

Será solicitado que o paciente retorne as ampolas não utilizadas, visando a contabilização da droga utilizada.

A 7.11 Mascaramento

Optou-se por realizar as aferições de desfechos clínicos de interesse (efetividade) e adversos (clínico) por outro médico que não aquele ciente do esquema terapêutico utilizado pelo paciente, a fim de preservar o mascaramento entre intervenção e desfecho. Pretende-se minimizar viéses de aferição dos desfechos diferenciados segundo o esquema de tratamento ao qual cada paciente pertence. Os resultados de exames laboratoriais serão fornecidos pelo laboratório de patologia clínica sem informação sobre o grupo de tratamento. Da mesma forma, para fins de análise, os grupos não serão identificados. O gerenciador de banco de dados preservará o sigilo desta informação codificando os grupos para fins de análise pelo(s) epidemiologista(s).

A 7.12 Critério para interrupção definitiva do tratamento do estudo

1. interrupção motivada por EA clínico, laboratorial ou eletrocardiográfico Grau 4
2. interrupção superior a 10 dias motivada por efeito adverso clínico, laboratorial ou eletrocardiográfico Grau$\leq$3
3. interrupção espontânea do uso da medicação prescrita em quantidade superior a cinco doses consecutivas, por falha de administração (não adesão)

A 7.13 Critérios para retirada do estudo (mas não da análise de dados)

1. interrupção definitiva do esquema de tratamento para o qual foi aleatorizado, por qualquer causa
2. gravidez
3. necessidade de introdução de droga imunossupressora ou potencialmente tóxica (quimioterapia para câncer, esquema para tuberculose ou hanseníase)
4. doença intercorrente, sem relação com a droga estudada, mas com manifestações equivalentes ou superiores a EA clínico Grau 3
5. necessidade de re-tratamento por má resposta terapêutica inicial ou tardia
6. desistência do paciente em prosseguir o estudo

Todos os pacientes receberão atendimento médico no IPEC (ambulatório de leishmanioses) durante a ocorrência de eventos adversos. Caso necessário terão a medicação suspensa por no máximo 10 dias e receberão tratamento sintomático até o alívio total dos eventos temporalmente associados ao uso da medicação. Poderão ter seu tratamento continuado em esquema alternativo (mas, para efeitos deste ensaio clínico serão

analisados pela intenção de tratar, isto é, segundo o grupo para o qual foram randomizados). Sempre que houver interrupção do tratamento, por motivo de segurança, por mais de 10 dias, o paciente será retirado do ensaio e reiniciará o tratamento por via intralesional (subprojeto G).

A 7.14 Procedimentos para quebra de sigilo

Os códigos de aleatorização gerados por software e utilizados para alocação de numeração e alocação dos pacientes poderão ser desvendados em caso de extrema necessidade e sempre considerando o bem estar do paciente. Para isto cópia do esquema de aleatorização ficará de posse de médico epidemiologista não ligado à equipe executora do atendimento e aos pacientes e /ou da análise dos dados que poderá ser contactada à qualquer hora para esclarecer em caso de emergência o tipo de dose e esquema ao qual o paciente pertence.

A 7.15 Monitoramento do estudo

A 7.15.1 Coordenadores e Monitor de Campo

O investigador Principal e coordenadores supervisionarão o trabalho de campo, controlando para desvios de qualidade e do presente protocolo. São itens importantes a serem monitorados: preenchimento adequado dos registros de desfechos e dos eventos adversos; adequação dos medicamentos armazenados; qualidade dos procedimentos relativos a exames laboratoriais; minimização de dados faltantes; envio periódico dos dados para digitação. Relatórios escritos de campo serão mantidos para apreciação pelos comitês. Relato de eventos adversos graves ao CEP /IPEC e decisão de interrupção do ensaio.

A 7.15.2 Comitê Externo

Será constituído um comitê de monitoramento externo do ensaio composto de três membros "experts" em tratamento de leishmaniose e execução de ensaios clínicos. Os membros serão escolhidos entre aqueles com currículo Lattes compatível com a atribuição. O comitê realizará auditoria das documentações e atividades pertinentes ao ensaio clínico, controlando para eventuais desvios do protocolo.

A 7.16 Controle da dispensação e armazenamento das medicações

As ampolas necessárias para o tratamento completo de toda a amostra serão armazenadas na Farmácia do IPEC. Um profissional da equipe treinado incluirá os pacientes, na consulta do dia 1, seguindo a lista de randomização do ensaio previamente fornecida pelo setor de Epidemiologia. Um farmacêutico treinado dispensará a medicação

prescrita pelo médico infectologista mediante a apresentação do cartão de inclusão no ensaio e da receita.

A 7.17 Plano de Análise dos dados

A análise dos dados será realizada seguindo o principio de intenção de tratamento, complementado pela análise por protocolo do desfecho principal. Os dados daqueles pacientes que necessitarem interrupção definitiva de um esquema serão analisados segundo o grupo ao qual foram alocados inicialmente, não sendo novamente alocados em outro grupo para reinício do tratamento (não haverá "cross-over" entre os grupos para efeitos deste estudo). A hipótese de não-inferioridade será testada baseada na margem de não-inferioridade de 15% com um intervalo de confiança unicaudal de 95%.

Serão descritas as frequências simples das variáveis categóricas (sexo; raça; localização das lesões; co-morbidade; eventos adversos; conclusão ou não do tratamento; ocorrência de recidiva) e as medidas de tendência central e dispersão das variáveis quantitativas contínuas (idade; número de lesões; tempo de evolução da doença desde o diagnóstico; tempo de tratamento em dias; tempo até alcançar os desfechos de efetividade inicial e tardio) para cada esquema antimonial utilizado (20 mg ou 5mg).

As frequências de cicatrização serão comparadas por teste para proporção do tipo qui-quadrado, o tempo médio até a cicatrização através de teste de comparação de médias (tipo t de student) três ou mais (ANOVA) e análise de sobrevida para desfechos que envolvam tempo em dias, caso necessário serão utilizados testes não paramétricos. Para avaliação da efetividade e de segurança, também serão estimados o risco relativo (RR), bem como a redução absoluta de risco (RAR) e a redução relativa de risco (RRR).

Para comparação pareada das proporções de cicatrização nos dias 20, 30 e 50 será empregado o teste de Mann-Whitney.

A 8. Considerações éticas

A 8.1 Riscos e benefícios

O principal benefício potencial deste ensaio consiste na possibilidade de subsidiar a utilização de doses de antimônio mais baixas, potencialmente menos tóxicas e de menor custo, para o tratamento de leishmaniose tegumentar que afeta um grande número de brasileiros, incluindo pacientes idosos e com co-morbidades (cardiopatias, nefropatias e hepatopatias). Os riscos consistem nos efeitos adversos gerais, que serão minuciosamente escrutinizados e tratados conforme o cronograma anexo. Este projeto será submetido ao CEP/IPEC e CONEP. Todos os pacientes assinarão um termo de consentimento livre e

esclarecido aprovado pelo CEP/IPEC. Este projeto segue as recomendações contidas na resolução 196/96 do Conselho Nacional de Saúde.

A 8.2 Termo de Consentimento Livre e Esclarecido

Em linguagem acessível e esclarecendo os objetivos, risco, benefícios e identificando os responsáveis pela pesquisa.

A 8.3 Incentivos para os voluntários

Os voluntários receberão auxílio transporte e as medicações.

A 9. Resultados esperados

Espera-se que o esquema o esquema alternativo com 5mg por 30 dias seja não-inferior em efetividade ao esquema atualmente recomendado no Brasil para o tratamento da leishmaniose cutânea (20mg Sb^{5+}/kg/dia por 20 dias).

Entretanto, espera-se encontrar diferença significativa na toxicidade dos diferentes esquemas, os quais devem apresentar efeitos adversos (em frequência e intensidade) na seguinte ordem decrescente: 1) 20mg Sb^{5+}/kg/dia por 20 dias; 2) 5mg Sb^{5+}/kg/dia por 30 dias contínuos.

Independente do esquema terapêutico, espera-se que as lesões localizadas acima dos joelhos encontrem-se epitelizadas por ocasião do término do tratamento, enquanto a epitelização das lesões localizadas na pernas e pés ocorra, com mais frequência, após o término do período de administração dos medicamentos, particularmente naqueles casos com insuficiência vascular associada. Também é esperado que, independente do esquema terapêutico, pacientes acima de 50 anos apresentem efeitos adversos com maior frequência e maior intensidade que pacientes mais jovens.

Os resultados deste projeto deverão ser publicados em periódicos indexados e em eventos científicos das áreas de parasitologia, biologia molecular, infectologia e medicina tropical. Os coordenadores e alguns pesquisadores serão responsáveis pela organização do manuscrito e comunicação dos resultados.

A 10. Apoio financeiro

Este projeto é financiado parcialmente com recursos aprovados pelo Edital MCT/CNPq/MS-SCTIE-DECIT 25/2006 - Estudo de Doenças Negligenciadas - e será submetido a outros editais pertinentes das agências de fomento.

A 11. Cooperação estrangeira, armazenamento de amostras biológicas e propriedade intelectual

Neste projeto não haverá cooperação com entidades estrangeiras nem armazenamento de amostras biológicas. Também não é esperado que haja pedido de patente de produtos e procedimentos.

REFERÊNCIAS BIBLIOGRÁFICAS

Adult AIDS Clinical Trials Group August, 1992. AIDS table for grading severity of adult adverse experiences. AACTC. www.aactg.s-3.com. Acessed July 6th, 2004.

Al Jaser M, el-Yazigi A, Croft SL 1995. Pharmacokinetics of antimony in patients treated with sodium stibogluconate for cutaneous leishmaniasis. *Pharmaceutical Research 12*: 113-116.

Alexander J, Russell DG 1992. The interaction of Leishmania species with macrophages. *Adv Parasitol 31*: 175-254.

Amato V, Amato J, Nicodemo A, Uip D, Amato-Neto V, Duarte M 1998. [Treatment of mucocutaneous leishmaniasis with pentamidine isothionate]. *Ann Dermatol Venereol 125*: 492-495.

Andrade L, Machado C, Chiari E, Pena S, Macedo A 1999. Differential tissue distribution of diverse clones of *Trypanosoma cruzi* in infected mice. *Mol Biochem Parasitol 100*: 163-172.

Antezana G, Zeballos R, Mendoza C, Lyevre P, Valda L, Cardenas F, Noriega I, Ugarte H, Dedet JP 1992. Electrocardiographic alterations during treatment of mucocutaneous leishmaniasis with meglumine antimoniate and allopurinol. *Trans R Soc Trop Med Hyg 86*: 31-33.

Antoine JC, Prina E, Jouanne C, Bongrand P 1990. Parasitophorous vacuoles of Leishmania amazonensis-infected macrophages maintain an acidic pH. *Infect Immun 58*: 779-787.

Aronson NE, Wortmann GW, Johnson SC, Jackson JE, Gasser Jr RA, Magill AJ, Endy TP, Coyne PE, Grogl M, Benson PM, Beard JS, Tally JD, Gambel JM, Kreutzer RD, Oster CN 1998. Safety and efficacy of intravenous sodium stibogluconate in the treatment of leishmaniasis: recent U.S. military experience. *Clinical Infectious Diseases 27*: 1457-1464.

Ashford RW 2000. The leishmaniases as emerging and reemerging zoonoses. *Int J Parasitol 30*: 1269-1281.

Aviles H, Belli A, Armijos R, Monroy FP, Harris E 1999. PCR detection and identification of Leishmania parasites in clinical specimens in Ecuador: a comparison with classical diagnostic methods. *J Parasitol 85*: 181-187.

Azeredo-Coutinho RB, Mendonca SC 2002. An intermittent schedule is better than continuous regimen of antimonial therapy for cutaneous leishmaniasis in the municipality of Rio de Janeiro, Brazil. *Revista da Sociedade Brasileira de Medicina Tropical 3*: 477-481.

Azeredo-Coutinho RB, Mendonça SCF 1997. Comparative study of two antimonial therapy schedules for treating cutaneous leishmaniasis. In XXIV Annual Meeting on Basic Research in Chagas Disease, Caxambu, 92, Memórias do Instituto Oswaldo Cruz, 11-14 November 1997.

Azeredo-Coutinho RBG 1999. Estudo comparativo de dois esquemas de tratamento antimonial da leishmaniose cutânea causada por Leishmania braziliensis no município do Rio de Janeiro*. Curso de Pós-graduação em Medicina Tropical*. Instituto Oswaldo Cruz, Fiocruz, Rio de Janeiro, p. 75.

Bacellar O, Lessa H, Schriefer A, Machado P, Ribeiro de Jesus A, Dutra WO, Gollob KJ, Carvalho EM 2002. Up-regulation of Th1-type responses in mucosal leishmaniasis patients. *Infect Immun 70*: 6734-6740.

Barkirtzief Z 1996. Identificando barreiras para aderência no tratamento da hanseníase. *Cadernos de Saúde Pública 12*: 497-505.

Barral-Netto M, Machado P, Bittencourt A, Barral A 1997. Recent advances in the pathophysiology and treatment of human cutaneous leishmaniasis. *Current Opinion in Dermatology 4*: 51-58.

Barros MBL, Schubach A, Francesconi-do-Valle AC, Gutierrez-Galhardo MC, Schubach TMP, Conceição-Silva F, Salgueiro MM, Mouta-Confort E, Reis RS, Madeira MF, Cuzzi T, Quintella LP, Passos JPS, Conceição MJ, Marzochi MCA 2005. Positive Montenegro skin test among patients with sporotrichosis in Rio de Janeiro. *Acta Tropica 93*: 41-47.

Beers MH 1997. Medicamentos para idosos. In PR Katz, *Geriatria Prática*, Revinter, Rio de Janeiro, p. 34-52.

Belli A, Rodriguez B, Aviles H, Harris E 1998. Simplified polymerase chain reaction detection of new world Leishmania in clinical specimens of cutaneous leishmaniasis. *Am J Trop Med Hyg 58*: 102-109.

Berman JD 1988. Chemotherapy for leishmaniasis: biochemical mechanisms, clinical efficacy, and future strategies. *Reviews Infectious Diseases 10*: 560-586.

Berman JD, Chulay JD, Hendricks LD, Oster CN 1982. Susceptibility of clinically sensitive and resistant Leishmania to pentavalent antimony in vitro. *Am J Trop Med Hyg 31*: 459-465.

Berman JD, Gallalee JF, Gallalee JV 1988. Pharmacokinetics of pentavalent antimony (Pentostam) in hamsters. *Am J Trop Med Hyg 39*: 41-45.

Berman JD, Lee LS 1983. Activity of oral drugs against Leishmania tropica in human macrophages in vitro. *Am J Trop Med Hyg 32*: 947-951.

Berman JD, Waddell D, Hanson BD 1985. Biochemical mechanisms of the antileishmanial activity of sodium stibogluconate. *Antimicrob Agents Chemother 27*: 916-920.

Berman JD, Wyler DJ 1980. An in vitro model for investigation of chemotherapeutic agents in leishmaniasis. *J Infect Dis 142*: 83-86.

Beverley SM, Ismach RB, Pratt DM 1987. Evolution of the genus Leishmania as revealed by comparisons of nuclear DNA restriction fragment patterns. *Proc Natl Acad Sci U S A 84*: 484-488.

Beverley SM, Turco SJ 1998. Lipophosphoglycan (LPG) and the identification of virulence genes in the protozoan parasite Leishmania. *Trends Microbiol 6*: 35-40.

Botega N 2001. *Prática psiquiátrica no hospital geral*. Artmed, Porto Alegre.

Brasil, Ministério da Saúde, Fundação Nacional de Saúde 2000. *Manual de Controle da Leishmaniose Tegumentar Americana*, Brasília, 62 pp.

Brasil, Ministério da Saúde, Fundação Nacional de Saúde, Centro Nacional de Epidemiologia, Coordenação Nacional de Dermatologia Sanitária 1997. Relatório da Oficina de Trabalho de Leishmanioses, Brasília.

Breniere SF, Telleria J, Bosseno MF, Buitrago R, Bastrenta B, Cuny G, Banuls AL, Brewster S, Barker DC 1999. Polymerase chain reaction-based identification of New World Leishmania species complexes by specific kDNA probes. *Acta Trop 73*: 283-293.

Brochu C, Wang J, Roy G, Messier N, Wang XY, Saravia NG, Ouellette M 2003. Antimony uptake systems in the protozoan parasite *Leishmania* and accumulation differences in antimony-resistant parasites. *Antimicrob Agents Chemother 47*: 3073-3079.

Brummitt CF, Porter JA, Herwaldt BL 1996. Reversible peripheral neuropathy associated with sodium stibogluconate therapy for American cutaneous leishmaniasis. *Clin Infect Dis 22*: 878-879.

Bryceson AD, Bray RS, Wolstencroft RA, Dumonde DC 1970. Cell mediated immunity in cutaneous leishmaniasis of the guinea-pig. *Trans R Soc Trop Med Hyg 64*: 472.

Bryceson AD, Chulay JD, Ho M, Mugambii M, Were JB, Muigai R, Chunge C, Gachihi G, Meme J, Anabwani G, et al. 1985a. Visceral leishmaniasis unresponsive to antimonial drugs. I. Clinical and immunological studies. *Trans R Soc Trop Med Hyg 79*: 700-704.

Bryceson AD, Chulay JD, Mugambi M, Were JB, Gachihi G, Chunge CN, Muigai R, Bhatt SM, Ho M, Spencer HC, Meme J, Anabwani G 1985b. Visceral leishmaniasis unresponsive to antimonial drugs. II. Response to high dosage sodium stibogluconate or prolonged treatment with pentamidine. *Trans R Soc Trop Med Hyg 79*: 705-714.

Cabrera M, Blackwell JM, Castes M, Trujillo D, Convit J, Shaw MA 2000. Immunotherapy with live BCG plus heat killed Leishmania induces a T helper 1-like response in American cutaneous leishmaniasis patients. *Parasite Immunol 22*: 73-79.

Cabrine-Santos M, Silva E, Chapadeiro E, Ramírez L 2001. *Trypanosoma cruzi*: characterization of reinfection and search for tissue tropism in hamsters (*Mesocricetus auratus*). *Exp Parasitol 99*: 160-167.

Callahan HL, Beverley SM 1991. Heavy metal resistance: a new role for P-glycoproteins in Leishmania. *J Biol Chem 266*: 18427-18430.

Callahan HL, Roberts WL, Rainey PM, Beverley SM 1994. The PGPA gene of Leishmania major mediates antimony (SbIII) resistance by decreasing influx and not by increasing efflux. *Mol Biochem Parasitol 68*: 145-149.

Carvalho EM, Barral A, Costa JM, Bittencourt A, Marsden P 1994. Clinical and immunopathological aspects of disseminated cutaneous leishmaniasis. *Acta Trop 56*: 315-325.

Carvalho EM, Teixeira RS, Johnson WD, Jr. 1981. Cell-mediated immunity in American visceral leishmaniasis: reversible immunosuppression during acute infection. *Infect Immun 33*: 498-500.

Carvalho JAM, Garcia RA 2003. [The aging process in the Brazilian population: a demographic approach]. *Cadernos de Saúde Pública 19*: 725-733.

Castes M, Agnelli A, Verde O, Rondon AJ 1983. Characterization of the cellular immune response in American cutaneous leishmaniasis. *Clin Immunol Immunopathol 27*: 176-186.

Castes M, Cabrera M, Ujillo DT, Convit J 1988. T-cell subpopulations, expression of interleukin-2 receptor, and production of interleukin-2 and gamma interferon in human American cutaneous leishmaniasis. *Journal of Clinical Microbiology 26*: 1207-1213.

Castes M, Moros Z, Martinez A, Trujillo D, Castellanos PL, Rondon AJ, Convit J 1989. Cell-mediated immunity in localized cutaneous leishmaniasis patients before and after treatment with immunotherapy or chemotherapy. *Parasite Immunol 11*: 211-222.

CENEPI 1997. *Informe Epidemiológico do SUS*

Chicharro C, Morales MA, Serra T, Ares M, Salas A, Alvar J 2002. Molecular epidemiology of Leishmania infantum on the island of Majorca: a comparison of phenotypic and genotypic tools. *Trans R Soc Trop Med Hyg 96 Suppl 1*: S93-99.

Chulay JD, Fleckenstein L, Smith DH 1988. Pharmacokinetics of antimony during treatment of visceral leishmaniasis with sodium stibogluconate or meglumine antimoniate. *Trans R Soc Trop Med Hyg 82*: 69-72.

Chulay JD, Spencer HC, Mugambi M 1985. Electrocardiographic changes during treatment of leishmaniasis with pentavalent antimony (sodium stibogluconate). *Am J Trop Med Hyg 34*: 702-709.

Claros P, Wienberg P, Gonzalez MA, Claros A, Claveria MA, Lopez P 1996. [Intralesional treatment of cutaneous leishmaniasis: a report of two cases]. *Acta Otorrinolaringol Esp 47*: 67-70.

Conceição-Silva F, Dorea RC, Pirmez C, Schubach A, Coutinho SG 1990. Quantitative study of *Leishmania braziliensis braziliensis* reactive T cells in peripheral blood and in the lesions of patients with American mucocutaneous leishmaniasis. *Clin Exp Immunol 79*: 221-226.

Convit J, Pinardi ME, Rondon AJ 1972. Diffuse cutaneous leishmaniasis: a disease due to an immunological defect of the host. *Trans R Soc Trop Med Hyg 66*: 603-610.

Correia D, Macedo VO, Carvalho EM, Barral A, Magalhaes AV, de Abreu MV, Orge ML, Marsden P 1996. [Comparative study of meglumine antimoniate, pentamidine isethionate and aminosidine sulfate in the treatment of primary skin lesions caused by Leishmania (Viannia) braziliensis]. *Rev Soc Bras Med Trop 29*: 447-453.

Costa JM, Vale KC, Franca F, Saldanha AC, Silva JO, Lago EL, Marsden PD, Magalhães AV, Silva CM, Serra Neto A 1990. [Spontaneous healing of leishmaniasis caused by *Leishmania Viannia braziliensis* in cutaneous lesions]. *Revista da Sociedade Brasileira de Medicina Tropical 23*: 205-208.

Coutinho SG, Da-Cruz AM, Bertho AL, Santiago MA, De-Luca P 1998. Immunologic patterns associated with cure in human American cutaneous leishmaniasis. *Braz J Med Biol Res 31*: 139-142.

Coutinho SG, Pirmez C, Da-Cruz AM 2002. Parasitological and immunological follow-up of American tegumentary leishmaniasis patients. *Trans R Soc Trop Med Hyg 96 Suppl 1*: S173-178.

Coutinho SG, Pirmez C, Mendonca SCF, Conceição-Silva F, Dorea RCC 1987. Pathogenesis and immunopathology of leishmaniasis. *Memórias do Instituto Oswaldo Cruz 82*: 214-228.

Cramer J, Mattson R, Prevey M, Scheyer R, Ouellette V 1989. How often is medication taken as prescribed? *JAMA 261*: 3273-3277.

Crofts MA 1976. Use of amphotericin B in mucocutaneous leishmaniasis. *J Trop Med Hyg 79*: 111-113.

Croop J, Gros P, Housman D 1988. Genetics of multidrug resistance. *Journal of Clinical Investigation 81*: 1303-1309.

Cullen W, McBride B, Reglinski J 1984. The reaction of methylarsenicals with thiols: Some biological implications. *Journal of Inorganic Biochemistry 21*: 179-194.

Cupolillo E, Brahim LR, Toaldo CB, de Oliveira-Neto MP, de Brito ME, Falqueto A, de Farias Naiff M, Grimaldi G, Jr. 2003. Genetic polymorphism and molecular epidemiology of Leishmania (Viannia) braziliensis from different hosts and geographic areas in Brazil. *J Clin Microbiol 41*: 3126-3132.

Cupolillo E, Grimaldi Jr. G, Momen H 1994. A general classificatiom of new world *Leishmania* using numeral zymotaxomomy. *American Journal Tropical Medicine Hygiene 50*: 296-311.

Da Silva RP, Hall BF, Joiner KA, Sacks DL 1989. CR1, the C3b receptor, mediates binding of infective Leishmania major metacyclic promastigotes to human macrophages. *J Immunol 143*: 617-622.

Da-Cruz AM, de Oliveira MP, De Luca PM, Mendonca SC, Coutinho SG 1996. Tumor necrosis factor-alpha in human american tegumentary leishmaniasis. *Mem Inst Oswaldo Cruz 91*: 225-229.

Da-Cruz AM, Machado ES, Menezes JA, Rutowitsch MS, Coutinho SG 1992. Cellular and humoral immune responses of a patient with American cutaneous leishmaniasis and AIDS. *Trans R Soc Trop Med Hyg 86*: 511-512.

de Bruijn MH, Barker DC 1992. Diagnosis of New World leishmaniasis: specific detection of species of the Leishmania braziliensis complex by amplification of kinetoplast DNA. *Acta Trop 52*: 45-58.

Deane LM, Grimaldi Jr. G 1985. Leishmaniasis in Brazil. In RS Bray, *Leishmania*, Elsevier, Amsterdan, p. 247-281.

Degrave W, Fernandes O, Campbell D, Bozza M, Lopes U 1994. Use of molecular probes and PCR for detection and typing of *Leishmania* - a mini-review. *Memórias do Instituto Oswaldo Cruz 89*: 463-469.

Deps PD, Viana MC, Falqueto A, Dietze R 2000. [Comparative assessment of the efficacy and toxicity of N-methyl- glucamine and BP88 sodium stibogluconate in the treatment of localized cutaneous leishmaniasis]. *Revista da Sociedade Brasileira de Medicina Tropical 33*: 535-543.

Desjardins M, Descoteaux A 1997. Inhibition of phagolysosomal biogenesis by the Leishmania lipophosphoglycan. *J Exp Med 185*: 2061-2068.

Desjeux P 2001. The increase in risk factors for leishmaniasis worldwide. *Trans R Soc Trop Med Hyg 95*: 239-243.

D'Oliveira A, Jr., Machado P, Bacellar O, Cheng LH, Almeida RP, Carvalho EM 2002. Evaluation of IFN-gamma and TNF-alpha as immunological markers of clinical outcome in cutaneous leishmaniasis. *Rev Soc Bras Med Trop 35*: 7-10.

Dujardin JC, Victoir K, De Doncker S, Guerbouj S, Arevalo J, Le Ray D 2002. Molecular epidemiology and diagnosis of Leishmania: what have we learnt from genome structure, dynamics and function? *Trans R Soc Trop Med Hyg 96 Suppl 1*: S81-86.

Durbar-Jacob J, Mortimer-Stephens MK 2001. Treatment adherence in chronic disease. *J Clin Epidemiol 54*: S57-S60.

D'Utra e Silva O 1915. Sobre a leishmaniose tegumentar e seu tratamento. *Memórias do Instituto Oswaldo Cruz 7*: 213-248.

Falqueto A, Sessa PA 1997. Leishmaniose Tegumentar Americana. In R Focaccia, *Veronesi Tratado de Infectologia*, Atheneu, São Paulo, p. 1221-1233.

Feldmann J, Haas K 2000. Sampling of trace volatile metal(loid) compounds in ambient air using polymer bags: A convenient method. *Anal Chem 72*: 4205-4211.

Fernandes O, Murthy VK, Kurath U, Degrave WM, Campbell DA 1994. Mini-exon gene variation in human pathogenic Leishmania species. *Mol Biochem Parasitol 66*: 261-271.

Ferreira-Pinto KC, Miranda-Vilela AL, Anacleto C, Fernandes AP, Abdo MC, Petrillo-Peixoto ML, Moreira ES 1996. Leishmania (V.) guyanensis: isolation and characterization of glucantime-resistant cell lines. *Can J Microbiol 42*: 944-949.

Filella M, Belzile N, Chen Y 2002. Antimony in the environment: a review focused on natural waters - I. Ocurrence. *Earth Sciences Reviews 57*: 125-176.

Filgueiras S, Deslandes S 1999. Avaliação das ações de aconselhamento. Análise de uma perspectiva de prevenção centrada na pessoa. *Cadernos de Saúde Pública 15*: 121-131.

Fletcher R, Fletcher S, Wagner E 1989. *Epidemiologia clínica*. Artes Médicas, Porto Alegre.

Franco D, Vago A, Chiari E, Meira F, Galvão L, Machado C 2003. *Trypanosoma cruzi*: mixture of two populations can modify virulence and tissue tropism in rat. *Exp Parasitol 104*: 54-61.

Franke ED, Wignall FS, Cruz ME, Rosales E, Tovar AA, Lucas CM, Llanos-Cuentas A, Berman JD 1990. Efficacy and toxicity of sodium stibogluconate for mucosal leishmaniasis. *Annals Internal Medicine 113*: 934-940.

Fundação Nacional de Saúde 2000. *Manual de Controle da Leishmaniose Tegumentar Americana, Ministério da Saúde*, Brasília, 62 pp.

Furtado T 1994. Leishmaniose Tegumentar Americana. In J Machado-Pinto, *Doenças infecciosas com manisfestações dermatológicas*, Editora Médica e Científica Ltda, Rio de Janeiro, p

Garcia R 2003. Os fatores de aderência ao tratamento farmacológico das hiperlipidemias em pacientes atendidos na Secretaria Municipal de Ribeirão Preto*. Faculdade de Medicina de Ribeirão Preto*. Universidade de São Paulo, Ribeirão Preto, p. 104.

Gebel T 1997. Arsenic and antimony: comparative approach on mechanistic toxicology. *Chemico-Biological Interactions 107*: 131-144.

Gebel T 1998. Human biomonitoring of arsenic and antimony in case of an elevated geogenic exposure. *Environmental Health Perspectives 106*: 33-39.

Georges E, Bradley G, Gariepy J, Ling V 1990. Detection of P-glycoprotein isoforms by gene-specific monoclonal antibodies. *Proc Natl Acad Sci U S A 87*: 152-156.

Gervasio A, Lavorante A, Moraes M, Giné M, Miranda C, Carrilho E 2003. Eletroforese capilar acoplada à espectrometria com plasma: uma ferramenta eficiente para a especiação. *Química Nova 26*: 65-74.

Giatti L, Barreto SM 2003. [Health, work, and aging in Brazil]. *Cadernos de Saúde Pública 19*: 759-771.

Gil V, Paya M, Asensio M, Torres M, Pastor R, Merino J 1999. Incumplimiento del tratamiento con antibióticos en infecciones agudas no graves. *Med Clin 112*: 731-773.

Goldberg A, Cohen G, Rubin A 1998. Physician assessment of patient compliance with compliance treatment. *Soc Sci Med 47*: 1873-1876.

Gomes M, Silva E, Macedo A, Vago A, Melo M 1997. LSSP-PCR for characterization of strains of *Entamoeba histolytica* isolated in Brazil. *Parasitology*: 517-520.

Gomes RF, Macedo AM, Pena SD, Melo MN 1995. Leishmania (Viannia) braziliensis: genetic relationships between strains isolated from different areas of Brazil as revealed by DNA fingerprinting and RAPD. *Exp Parasitol 80*: 681-687.

Goodwin LG 1995. Pentostan (sodium stibogluconate); a 50-year personal reminiscence. *Transactions Royal Society Tropical Medicine Hygiene 89*: 339-341.

Grimaldi G, Jr., Tesh RB 1993. Leishmaniases of the New World: current concepts and implications for future research. *Clin Microbiol Rev 6*: 230-250.

Grimaldi G, McMahon-Pratt D 1996. Monoclonal antibodies for the identification of New World *Leishmania* species. *Memórias do Instituto Oswaldo Cruz 91*: 37-42.

Grogl M, Martin RK, Oduola AM, Milhous WK, Kyle DE 1991. Characteristics of multidrug resistance in Plasmodium and Leishmania: detection of P-glycoprotein-like components. *Am J Trop Med Hyg 45*: 98-111.

Grogl M, Oduola AM, Cordero LD, Kyle DE 1989. *Leishmania* spp.: development of pentostam-resistant clones in vitro by discontinuous drug exposure. *Exp Parasitol 69*: 78-90.

Grogl M, Thomason TN, Franke ED 1992. Drug resistance in leishmaniasis: its implication in systemic chemotherapy of cutaneous and mucocutaneous disease. *Am J Trop Med Hyg 47*: 117-126.

Guerbouj S, Victoir K, Guizani I, Seridi N, Nuwayri-Salti N, Belkaid M, Ismail RB, Le Ray D, Dujardin JC 2001. Gp63 gene polymorphism and population structure of Leishmania donovani complex: influence of the host selection pressure? *Parasitology 122 Pt 1*: 25-35.

Guevara P, Alonso G, Silveira JF, Mello M, Scorza JV, Añez N, Ramirez JL 1992. Identification of new world *Leishmania* using ribosomal gene spacer probes. *Molecular and Biochemical Parasitology 56*: 15-26.

Gupta P 1990. Electrocardiographic changes occurring after brief antimony administration in the presence of dilated cardiomyopathy. *Postgrad Med J 66*: 1089.

Halim MA, Alfurayh O, Kalin ME, Dammas S, al-Eisa A, Damanhouri G 1993. Successful treatment of visceral leishmaniasis with allopurinol plus ketoconazole in a renal transplant recipient after the occurrence of pancreatitis due to stibogluconate. *Clinical Infectious Diseases 16*: 397-399.

Hanafi R, Barhoumi M, Ali SB, Guizani I 2001. Molecular analyses of Old World Leishmania RAPD markers and development of a PCR assay selective for parasites of the L. donovani species Complex. *Exp Parasitol 98*: 90-99.

Handman E, Goding JW 1985. The Leishmania receptor for macrophages is a lipid-containing glycoconjugate. *Embo J 4*: 329-336.

Harms G, Chehade AK, Douba M, Roepke M, Mouakeh A, Rosenkaimer F, Bienzle U 1991. A randomized trial comparing a pentavalent antimonial drug and recombinant interferon-gamma in the local treatment of cutaneous leishmaniasis. *Trans R Soc Trop Med Hyg 85*: 214-216.

Haynes R 1981a. Determinants of compliance: the disease end mechanics of treatment. In R Haynes, D Taylor, D Sackett (eds), *Compliance in health care*, The Johns Hopkins University Press, Baltimore, p. 49-61.

Haynes R 1981b. Introduction. In R Haynes, D Taylor, D Sackett (eds), *Compliance in health care*, The Johns Hopkins University Press, Baltimore, p. 1-7.

Hepburn NC 2000. Cutaneous leishmaniasis. *Clin Exp Dermatol 25*: 363-370.

Hepburn NC, Nolan J, Fenn L, Herd RM, Neilson JM, Sutherland GR, Fox KA 1994a. Cardiac effects of sodium stibogluconate: myocardial, electrophysiological and biochemical studies. *Qjm 87*: 465-472.

Hepburn NC, Siddique I, Howie AF, Beckett GJ, Hayes PC 1994b. Hepatotoxicity of sodium stibogluconate therapy for American cutaneous leishmaniasis. *Trans R Soc Trop Med Hyg 88*: 453-455.

Herwaldt BL 1999. Leishmaniasis. *Lancet 354*: 1191-1199.

Herwaldt BL, Berman JD 1992. Recommendations for treating leishmaniasis with sodium stibogluconate (Pentostam) and review of pertinent clinical studies. *American Journal of Tropical Medicine and Hygiene 46*: 296-306.

IBGE 1981. *Censo Demográfico 1980*. Fundação Instituto Brasileiro de Geografia e Estatística (IBGE), Rio de Janeiro.

IBGE 2001. *Censo Demográfico 2000*. Fundação Instituto Brasileiro de Geografia e Estatística (IBGE), Rio de Janeiro.

Ishikawa EA, Silveira FT, Magalhaes AL, Guerra junior RB, Melo MN, Gomes R, Silveira TG, Shaw JJ 2002. Genetic variation in populations of Leishmania species in Brazil. *Trans R Soc Trop Med Hyg 96 Suppl 1*: S111-121.

Jackson PR, Lawrie JM, Stiteler JM, Hawkins DW, Wohlhieter JA, Rowton ED 1986. Detection and characterization of Leishmania species and strains from mammals and vectors by hybridization and restriction endonuclease digestion of kinetoplast DNA. *Vet Parasitol 20*: 195-215.

Jones TC, Johnson WD, Jr., Barretto AC, Lago E, Badaro R, Cerf B, Reed SG, Netto EM, Tada MS, Franca TF, Wiese K, Golightly L, Fikrig E, Costa JML, Cuba CC, Marsden PD 1987. Epidemiology of American cutaneous leishmaniasis due to *Leishmania braziliensis braziliensis*. *Journal of Infectious Diseases 156*: 73-83.

Krachler M, Emons H 2001. Speciation analysis of antimony by high-performance liquid chromatography inductively coupled plasma mass spectrometry using ultrasonic nebulization. *Analytica Chimica Acta 429*: 125-133.

Kubba R, al-Gindan Y, el-Hassan AM, Omer AH, Kutty MK, Saeed MB 1988. Dissemination in cutaneous leishmaniasis. II. Satellite papules and subcutaneous induration. *Int J Dermatol 27*: 702-706.

Kubba R, el-Hassan AM, Al-Gindan Y, Omer AH, Kutty MK, Saeed MB 1987. Dissemination in cutaneous leishmaniasis. I. Subcutaneous nodules. *Int J Dermatol 26*: 300-304.

Kurita G, Pimenta C 2003. Adesão ao tratamento da dor crônica: estudo de varáveis demográficas, terapêuticas e psicossociais. *Arq Neuropsiquiatr 61*: 416-425.

Lainson R 1983. The American leishmaniases: some observations on their ecology and epidemiology. *Transactions Royal Society Tropical Medicine Hygiene 77*: 569-596.

Lainson R, Shaw JJ 1987. Evolution, classification and geographical distribution. In K Killick-Kendrick, *The leishmaniasis in biology and medicine*, Academic Press, London, p. 1-120.

Legare D, Hettema E, Ouellette M 1994. The P-glycoprotein-related gene family in *Leishmania*. *Mol Biochem Parasitol 68*: 81-91.

Legare D, Papadopoulou B, Roy G, Mukhopadhyay R, Haimeur A, Dey S, Grondin K, Brochu C, Rosen BP, Ouellette M 1997. Efflux systems and increased trypanothione levels in arsenite-resistant Leishmania. *Exp Parasitol 87*: 275-282.

Leite J, Dracheler M, Centeno M, Pinheiro C, Silveira V 2002. Desenvolvimento de uma escala de auto-eficácia para adesão ao tratamento anti-retroviral. *Psicol Reflex Crit 15*: 121-133.

Leite S, Vasconcellos M 2003. Adesão à terapêutica medicamentosa: elementos para discussão de conceitos e pressupostos adotados na literatura. *Ciência & Saúde Coletiva 8*: 775-782.

Levy R, Feld A 1999. Increasing patient adherence to gastroenterology treatment and prevention regimens. *Am J Gastroenterol 94*: 1733-1742.

Llanos-Cuentas EA, Arana M, Cuba CAC, Rosa AC, Marsden PD 1985. Leishmaniasis cutanea diseminada asociada a metastasis en mucosas, causada por *Leishmania braziliensis braziliensis*: fracaso en el hallazgo de parasitos circulantes. *Rev Soc Bras Med Trop 18*: 271-272.

Lopes UG, Momen H, Grimaldi Jr. G, Marzochi MCA, Pacheco RS, Morel CM 1984. Schizodeme and zymodeme characterization of *Leishmania* in the investigation of focci of visceral and cutaneous leishmaniasis. *Journal of Parasitology 70*: 89-98.

Lopez M, Inga R, Cangalaya M, Echevarria J, Llanos-Cuentas A, Orrego C, Arevalo J 1993. Diagnosis of Leishmania using the polymerase chain reaction: a simplified procedure for field work. *Am J Trop Med Hyg 49*: 348-356.

Lyons LW, Johnston CB, Covinsky KE, Resnick NM 2001. Geriatric Medicine. In MA Papadakis, *Current Medical Diagnosis & Treatment*, McGraw-Hill, New York, p. 44-61.

Machado P, Araujo C, Da Silva AT, Almeida RP, D'Oliveira Jr A, Bittencourt A, Carvalho EM 2002. Failure of early treatment of cutaneous leishmaniasis in preventing the development of an ulcer. *Clin Infect Dis 34*: E69-73.

Marsden PD 1979. Current concepts in parasitology. Leishmaniasis. *N Engl J Med 300*: 350-352.

Marsden PD 1985. Pentavalent antimonials: old drugs for new diseases. *Revista da Sociedade Brasileira de Medicina Tropical 18*: 187-198.

Marsden PD 1986. Mucosal leishmaniasis ("espundia" Escomel, 1911). *Trans R Soc Trop Med Hyg 80*: 859-876.

Marsden PD, Jones TC 1985. Clinical manifestations, diagnosis and treatment of leishmaniasis. In RS Bray, *Leishmaniasis*, Elsevier, London, p. 183-198.

Marsden PD, Netto EM, Badaro R, Cuba CA, Costa JL, Barreto AC 1986. Apparent cure of a difficult treatment problem in a patient with mucosal leishmaniasis. *Am J Trop Med Hyg 35*: 449.

Marsden PD, Tada MS, Barreto AC, Cuba CC 1984. Spontaneous healing of Leishmania braziliensis braziliensis skin ulcers. *Trans R Soc Trop Med Hyg 78*: 561-562.

Martinez E, Alonso V, Quispe A, Thomas MC, Alonso R, Pinero JE, Gonzalez AC, Ortega A, Valladares B 2003. RAPD method useful for distinguishing Leishmania species: design of specific primers for L. braziliensis. *Parasitology 127*: 513-517.

Marzochi MAC, Marzochi KBF 1994. Tegumentary and visceral leishmaniasis in Brazil. Emerging anthropozoonosis and possibilities for their control. *Cadernos de Saúde Pública 10*: 359-375.

Marzochi MC, Coutinho SG, De Souza WJ, De Toledo LM, Grimaldi Junior G, Momen H, Pacheco R, Sabroza PC, De Souza MA, Rangel Junior FB, Tramontano NC 1985. Canine visceral leishmaniasis in Rio de Janeiro, Brazil. Clinical, parasitological, therapeutical and epidemiological findings (1977-1983). *Memórias do Instituto Oswaldo Cruz 80*: 349-357.

Marzochi MCA 1992. Leishmanioses no Brasil: As leishmanioses tegumentares. *Jornal Brasileiro de Medicina 63*: 82-104.

Matos DS, Azeredo-Coutinho RB, Schubach A, Conceição-Silva F, Baptista C, Moreira JS, Mendonça SC 2005. Differential interferon- gamma production characterizes the cytokine responses to Leishmania and Mycobacterium leprae antigens in concomitant mucocutaneous leishmaniasis and lepromatous leprosy. *Clinical Infectious Diseases 40*: e5-12.

McBride MO, Linney M, Davidson RN, Weber JN 1995. Pancreatic necrosis following treatment of leishmaniasis with sodium stibogluconate. *Clin Infect Dis 21*: 710.

McMahon-Pratt D, David JR 1981. Monoclonal antibodies that distinguish between New World species of Leishmania. *Nature 291*: 581-583.

McMahon-Pratt D, Jaffe CL, Bennett E, David JR, Grimaldi Jr. G 1986. Studies employing monoclonal antibodies for the analysis of the genus *Leishmania* Ross, 1903. In JA Rioux, *Leishmania Taxonomy and Phylogeny*, IMEEE, Montpellier, p. 173-178.

Mendonça MG, De Brito ME, Rodrigues EH, Bandeira V, Jardim ML, Abath FG 2004. Persistence of leishmania parasites in scars after clinical cure of american cutaneous leishmaniasis: is there a sterile cure? *J Infect Dis 189*: 1018-1023.

Mendonça SC, Coutinho SG, Amendoeira RR, Marzochi MC, Pirmez C 1986. Human American cutaneous leishmaniasis (*Leishmania b. braziliensis*) in Brazil: lymphoproliferative responses and influence of therapy. *Clinical and Experimental Immunology 64*: 269-276.

Mendonça SC, Russell DG, Coutinho SG 1991. Analysis of the human T cell responsiveness to purified antigens of Leishmania: lipophosphoglycan (LPG) and glycoprotein 63 (gp 63). *Clinical Experimental Immunology 83*: 472-478.

Mendonça SC, Souza WJ, Nunes MP, Marzochi MC, Coutinho SG 1988. Indirect immunofluorescence test in New World leishmaniasis: serological and clinical relationship. *Mem Inst Oswaldo Cruz 83*: 347-355.

Mendoza-Leon A, Havercroft JC, Barker DC 1995. The RFLP analysis of the beta-tubulin gene region in New World Leishmania. *Parasitology 111*: 1-9.

Miekeley N, Mortari SR, Schubach AO 2002. Monitoring of total antimony and its species by ICP-MS and on-line ion chromatography in biological samples from patients treated for leishmaniasis. *Analytical Bioanalytical Chemistry 372*: 495-502.

Misago C, Marshall T, Fonseca W, Kikwood B 1997. Out-patient drug treatment of pneumonia among children under two years of age in Fortaleza, Brazil. *Cadernos de Saúde Pública 13*: 37-43.

Momen H, Grimaldi Jr G, Pacheco RS, Jaffe CL, McMahon-Pratt D, Marzochi MC 1985. Brazilian *Leishmania* stocks phenotypically similar to *Leishmania major*. *American Journal Tropical Medicine Hygiene 34*: 1076-1084.

Monjour L, Neogy AB, Vouldoukis I, Silva OA, Boisnic S, Brito ME, Lesot A, Vignot N, Martins JS, Jardim ML 1994. Exploitation of parasite derived antigen in therapeutic success of human cutaneous leishmaniasis in Brazil. *Mem Inst Oswaldo Cruz 89*: 479-483.

Moreira JS 1994. Tese. Estudo da Laringite Leishmaniótica. *Departamento de Cirurgia*. Pontifícia Universidade Católica do Rio de Janeiro, Rio de Janeiro, p. 118.

Morisky D, Levine M, Green L, Smith C 1982. Health education program effects on the managements of hipertension in the elderly. *Archives Internal Medicine 172*: 1335-1838.

Mortari SR 2001. Determinação da concentração total de antimônio e de suas espécies químicas em amostras clínicas de pacientes com leishmanioses. *Departamento de Química*. Pontifícia Universidade Católica (PUC), Rio de Janeiro, p. 142.

Nakashima S 1980. Selective determination of antimony(III) and antimony(V) by atomic-absorption spectrophotometry following stibine generation. *Analyst 105*: 732-733.

Navin TR, Arana BA, Arana FE, Berman JD, Chajon JF 1992. Placebo-controlled clinical trial of sodium stibogluconate (Pentostam) versus ketoconazole for treating cutaneous leishmaniasis in Guatemala. *Journal of Infectious Diseases 165*: 528-534.

Nigro G, Angelini G, Grosso S, Caula G, Sategna-Guidetti C 2001. Psychatric predictors of non compliance in inflammatory bowel disease. *J Clin Gastroenterol 32*: 61-68.

Oliveira FS, Pirmez C, Pires MQ, Brazil RP, Pacheco RS 2005. PCR-based diagnosis for detection of Leishmania in skin and blood of rodents from an endemic area of cutaneous and visceral leishmaniasis in Brazil. *Vet Parasitol 129*: 219-227.

Oliveira M, Caballero O, Vago A, Harskeerl R, Romanha A, Pena S, Simpson A, Koury M 2003. Low-stringency single specific primer PCR for identification of *Leptospira*. *J Med Microbiol 52*: 127-135.

Oliveira-Neto MP, Mattos M, Pirmez C, Fernandes O, Goncalves-Costa SC, Souza CF, Grimaldi G, Jr. 2000. Mucosal leishmaniasis ("espundia") responsive to low dose of N-methyl glucamine (Glucantime) in Rio de Janeiro, Brazil. *Rev Inst Med Trop Sao Paulo 42*: 321-325.

Oliveira-Neto MP, Schubach A, Araujo ML, Pirmez C 1996. High and low doses of antimony (Sb^{v}) in American cutaneous leishmaniasis. A five years follow-up study of 15 patients. *Memórias do Instituto Oswaldo Cruz 91*: 207-209.

Oliveira-Neto MP, Schubach A, Mattos M, Gonçalves Da Costa SC, Pirmez C 1997a. Intralesional therapy of American cutaneous leishmaniasis with pentavalent antimony in Rio de Janeiro, Brazil - an area of *Leishmania (V.) braziliensis* transmission. *International Journal Dermatology 36*: 463-468.

Oliveira-Neto MP, Schubach A, Mattos M, Goncalves-Costa SC, Pirmez C 1997b. A low dose antimony treatment In 159 patients with American cutaneous leishmaniasis. Extensive follow-up studies (up to 10 years). *American Journal Tropical Medicine Hygiene 57*: 651-655.

Oliveira-Neto MP, Schubach A, Mattos M, Goncalves-Costa SC, Pirmez C 1997c. Treatment of American cutaneous leishmaniasis: a comparison between low dosage (5mg/kg/day) and high dosage (20mg/kg/day) antimony regimens. *Pathologie Biologie 45*: 496-469.

Ooteman M, Vago A, Koury M 2004. Potencial application of low-stringency single primer PCR in the identification of *Leptospira* in the serum of patients with suspected leptospirosis. *Can J Microbiol 50*: 1073-1079.

Osorio Y, Gonzalez SJ, Gama VL, Travi BL 1998. Reinfection in American cutaneous leishmaniasis: evaluation of clinical outcomes in the hamster model. *Mem Inst Oswaldo Cruz 93*: 353-356.

Oster CN, Chulay JD, Hendricks LD, Pamplin CL, 3rd, Ballou WR, Berman JD, Takafuji ET, Tramont EC, Canfield CJ 1985. American cutaneous leishmaniasis: a comparison of three sodium stibogluconate treatment schedules. *American Journal Tropical Medicine Hygiene 34*: 856-860.

Osterberg L, Blaschke T 2005. Adherence to medication. *New England Journal of Medicine 353*: 487-497.

Ouellette M, Papadopoulou B 1993. Mechanisms of drug resistance in *Leishmania*. *Parasitol Today 9*: 150-153.

Pacheco R, Brito C, Sarquis O, Pires M, Borges-Pereira J, Lima M 2005. Genetic heterogeneity in *Trypanosoma cruzi* stains from naturally infected triatomine vectors in Northeastern Brazil: Epidemiological implications. *Biochemical Genetics 43*: 519-530.

Pacheco RS, Brito CM 1999. Reflections on the population dynamics of Trypanosoma cruzi: heterogeneity versus plasticity. *Mem Inst Oswaldo Cruz 94*: 199-201.

Pacheco RS, Fernandes O, Salinas G, Segura I, Momen H, Degrave W, Saravia NG, Campbell DA 2000. Intraspecific heterogeneity in the mini-exon gene localization of Leishmania (Viannia) panamensis and Leishmania (Viannia) guyanensis from Colombia. *J Parasitol 86*: 1250-1253.

Pacheco RS, Lopes UG, Morel CM, Grimaldi Jr. G, Momen H 1986. Schizodeme analysis of *Leishmania* and comparison with some phenotypic techniques. In AJ Rioux, *Leishmania Taxonomie et Phylogenese Application Eco-Epidemiologique*, IMEEE, Montpellier, p. 57-65.

Passos VM, Barreto SM, Romanha AJ, Krettli AU, Volpini AC, Gontijo CM, Falcao AL, Lima-Costa MF 2001. [Cutaneous leishmaniasis in the Metropolitan Region of Belo Horizonte: clinical, laboratorial, therapeutic and prospective aspects]. *Rev Soc Bras Med Trop 34*: 5-12.

Pearson RD, Sousa AQ 1996. Clinical spectrum of Leishmaniasis. *Clin Infect Dis 22*: 1-13.

Pena SDJ, Barreto G, Vago AR, De Marco L, Reinach FC, Dias Neto E, Simpson AJG 1984. Sequence-specific "gene signatures" can be obtained by PCR with single specific primers at low stringency. *Proceedings National Academy Sciences United States America 91*: 1946-1949.

Pereira SB, Fonseca HHR 1994. Leishmaniose Tegumentar Americana: Epidemiologia e controle. *Revista da Sociedade Brasileira de Medicina Tropical 27*: 45-50.

Pessôa SB, Barretto MP 1948. *Leishmaniose Tegumentar Americana*. Ministério da Educação e Saúde, Serviço de Documentação, Rio de Janeiro, 527 pp.

Prasad LS, Sen S 1996. Migration of Leishmania donovani amastigotes in the cerebrospinal fluid. *Am J Trop Med Hyg 55*: 652-654.

Pupo JA 1946. Estudo clínico de leishmaniose tegumentar americana (*Leishmania braziliensis* - Vianna 1911). *Revista do Hospital das Clínicas 1*: 113-164.

Ramalhinho I 1994. Adesão à terapêutica anti-hipertensiva: contributo para seu estudo. *Faculdade de Ciências Médicas*. Universidade Nova de Lisboa, Lisboa, p. 84.

Rees PH, Keating MI, Kager PA, Hockmeyer WT 1980. Renal clearance of pentavalent antimony (sodium stibogluconate). *Lancet 2*: 226-229.

Ribeiro AL, Drummond JB, Volpini AC, Andrade AC, Passos VM 1999. Electrocardiographic changes during low-dose, short-term therapy of cutaneous leishmaniasis with the pentavalent antimonial meglumine. *Brazilian Journal Medical Biological Research 32*: 297-301.

Ribeiro S, Amado S, Camelier A, Fernandes M, Shenckman S 2000. Estudo caso-controle de indicadores de abandono em doentes com tuberculose. *J Pneumologia 26*

Rioux JA, Lanotte G, Serres E, Pratlong F, Bastien P, Perieres J 1990. Taxonomy of Leishmania. Use of isoenzymes. Suggestions for a new classification. *Ann Parasitol Hum Comp 65*: 111-125.

Roberts WL, Berman JD, Rainey PM 1995. In vitro antileishmanial properties of tri- and pentavalent antimonial preparations. *Antimicrobial Agents Chemotherapy 39*: 1234-1239.

Rocha C, Faggiani F, Schroeter G, Souza A, De Carli G 2006. Adesão à Prescrição Médica em Idosos de Porto Alegre. Revista Ciência & Saúde Coletiva da Associação Brasileira de Pós-Graduação em Saúde Coletiva. Pontifícia Universidade Católica do Rio Grande do Sul. http://www.abrasco.org.br/cienciaesaudecoletiva/artigos/artigo_int.php?id_artigo=488.

Rodgers MR, Popper SJ, Wirth DF 1990. Amplification of kinetoplast DNA as a tool in the detection and diagnosis of Leishmania. *Exp Parasitol 71*: 267-275.

Rodrigues AM, Hueb M, Santos TA, Fontes CJ 2006. [Factors associated with treatment failure of cutaneous leishmaniasis with meglumine antimoniate]. *Rev Soc Bras Med Trop 39*: 139-145.

Rodrigues ML, Costa RS, Souza CS, Foss NT, Roselino AM 1999. Nephrotoxicity attributed to meglumine antimoniate (Glucantime) in the treatment of generalized cutaneous leishmaniasis. *Revista do Instituto de Medicina Tropical de São Paulo 41*: 33-37.

Rodriguez LV, Dedet JP, Paredes V, Mendoza C, Cardenas F 1995. A randomized trial of amphotericin B alone or in combination with itraconazole in the treatment of mucocutaneous leishmaniasis. *Mem Inst Oswaldo Cruz 90*: 525-528.

Rodriguez N, Guzman B, Rodas A, Takiff H, Bloom BR, Convit J 1994. Diagnosis of cutaneous leishmaniasis and species discrimination of parasites by PCR and hybridization. *J Clin Microbiol 32*: 2246-2252.

Rodriguez NM, De Guglielmo Z, Barrios MA, Barrios RM, Zerpa O, Feliciangeli MD 2005. Genetic homogeneity within Leishmania (L.) infantum isolated from human and dogs: the relationship with the sandfly fauna distribution in endemic areas of Nueva Esparta State, Venezuela. *Parasitology 130*: 611-619.

Rojas R, Valderama L, Valderama M, Varona M, Ouellette M, Saravia N 2006. Resistance to antimony and treatment failure in human *Leishmania (Viannia)* infection. *Journal Infectious Diseases 193*: 1375-1383.

Romero GAS, Hueb M, D'Oliveira Jr A, Schubach A 2001a. Simpósio sobre Tratamento das Leishmanioses. *Revista da Sociedade Brasileira de Medicina Tropical 34*: 58-68.

Romero GAS, Schubach A, Oliveira-Neto MP, Hueb M, Dietze R 2001b. Relatório Final do Simpósio: Tratamento das leishmanioses: Existem evidências para recomendar esquemas de tratamento universais na leishmaniose tegumentar? In V Reunião de Pesquisa Aplicada em Leishmanioses, Uberaba.

Rozenfeld S 2003. [Prevalence, associated factors, and misuse of medication in the elderly: a review]. *Cadernos de Saúde Pública 19*: 717-724.

Rozenfeld S, Pepe VLE 1992/93. *Guia Terapêutico Ambulatorial*. Artes Médicas, Porto Alegre, 404 pp.

Russell DG, Wilhelm H 1986. The involvement of the major surface glycoprotein (gp63) of Leishmania promastigotes in attachment to macrophages. *J Immunol 136*: 2613-2620.

Saenz RE, de Rodriguez CG, Johnson CM, Berman JD 1991. Efficacy and toxicity of pentostam against Panamanian mucosal leishmaniasis. *American Journal Tropical Medicine Hygiene 44*: 394-398.

Saiki RB, Scharf S, Fallona F, Mullis KB, Horn GT, Erlich H, Arhein N 1985. Enzimatic amplification of b-globin genomic sequences and restriction site diagnosis of sickle cell anemia. *Science 230*: 1350-1354.

Saldanha AC, Romero GA, Guerra C, Merchan-Hamann E, Macedo VO 2000. [Comparative study between sodium stibogluconate BP 88 and meglumine antimoniate in cutaneous leishmaniasis treatment. II. Biochemical and cardiac toxicity]. *Revista da Sociedade Brasileira de Medicina Tropical 33*: 383-388.

Sampaio RN, Paula CD, Sampaio JH, Furtado RS, Leal PP, Rosa TT, Rodrigues ME, Veiga JP 1997. [The evaluation of the tolerance and nephrotoxicity of pentavalent antimony administered in a dose of 40mg Sb^V/kg/day, 12/12hr, for 30 days in the mucocutaneous form of leishmaniasis. *Revista da Sociedade Brasileira de Medicina Tropical 30*: 457-463.

Sampaio RN, Salaro CP, Resende P, Paula CD 2002. [American cutaneous leishmaniasis associated with HIV/AIDS: report of four clinical cases]. *Rev Soc Bras Med Trop 35*: 651-654.

Sampaio SA, Castro RM, Dillon NL, Martins JE 1971. Treatment of mucocutaneous (American) leishmaniasis with amphotericin B: report of 70 cases. *Int J Dermatol 10*: 179-181.

Santos MA, Marques RC, Farias CA, Vasconcelos DM, Stewart JM, Costa DL, Costa CH 2002. Predictors of an unsatisfactory response to pentavalent antimony in the treatment of American visceral leishmaniasis. *Rev Soc Bras Med Trop 35*: 629-633.

Saravia NG, Weigle K, Segura I, Giannini SH, Pacheco R, Labrada LA, Goncalves A 1990. Recurrent lesions in human *Leishmania braziliensis* infection - reactivation or reinfection? *Lancet 336*: 398-402.

Schubach A 1990. Tese. Estudo da evolução da leishmaniose tegumentar americana em pacientes tratados. *Medicina Tropical*. Instituto Oswaldo Cruz, FIOCRUZ, Rio de Janeiro, p. 141.

Schubach A, Cuzzi-Maya T, Oliveira AV, Sartori A, de Oliveira-Neto MP, Mattos MS, Araujo ML, Souza WJ, Haddad F, Perez Mde A, Pacheco RS, Momen H, Coutinho SG, de Almeida Marzochi MC, Marzochi KB, da Costa SC 2001. Leishmanial antigens in the diagnosis of active lesions and ancient scars of American tegumentary leishmaniasis patients. *Mem Inst Oswaldo Cruz 96*: 987-996.

Schubach A, Haddad F, Oliveira-Neto MP, Degrave W, Pirmez C, Grimaldi G, Jr., Fernandes O 1998a. Detection of *Leishmania* DNA by the polymerase chain reaction in scars of treated human patients. *Journal of Infectious Diseases 178*: 911-914.

Schubach A, Marzochi MC, Cuzzi-Maya T, Oliveira AV, Araújo ML, Oliveira AL, Pacheco RS, Momen H, Conceição-Silva F, Coutinho SG, Marzochi KB 1998b. Cutaneous scars in American tegumentary leishmaniasis patients: a site of *Leishmania (Viannia) braziliensis* persistence and viability eleven years after antimonial therapy and clinical cure. *American Journal Tropical Medicine Hygiene 58*: 824-827.

Schubach A, Miekeley N, Mortari SR, Moreira JS, Conceição-Silva F, Salgueiro MM, Campos FV, Marzochi KBF, Marzochi MCA 2002. Estudos sobre o metabolismo de antimônio e de suas espécies químicas no tratamento da Leishmaniose Tegumentar Americana com baixas doses de antimônio. *Revista da Sociedade Brasileira de Medicina Tropical 35*: 102-103.

Schubach AO, Marzochi KBF, Moreira JS, Schubach TMP, Araújo ML, Francesconi-do-Vale AC, Passos SRL, Marzochi MCA 2005. Retrospective study of 151 patients with cutaneous leishmaniasis treated with meglumine antimoniate. *Revista da Sociedade Brasileira de Medicina Tropical 38*: 213-217.

Seaton RA, Morrison J, Man I, Watson J, Nathwani D 1999. Out-patient parenteral antimicrobial therapy - a viable option for the management of cutaneous leishmaniasis. *Quarterly Journal Medicine 92*: 659-667.

Sergiev VP, Uzbekov MK, Polevoi NI 1968. [A case of relapse of zoonotic cutaneous leishmaniasis running a course of tuberculoid type]. *Med Parazitol (Mosk) 37*: 331-332.

Sharples CE, Shaw MA, Castes M, Convit J, Blackwell JM 1994. Immune response in healthy volunteers vaccinated with BCG plus killed leishmanial promastigotes: antibody responses to mycobacterial and leishmanial antigens. *Vaccine 12*: 1402-1412.

Sharquie KE 1995. A new intralesional therapy of cutaneous leishmaniasis with hypertonic sodium chloride solution. *J Dermatol 22*: 732-737.

Shaw JJ 1994. Taxonomy of the genus Leishmania: present and future trends and their implications. *Mem Inst Oswaldo Cruz 89*: 471-478.

Shaw JJ, Lainson R, McMahon-Pratt D, David JR 1986. Serodeme of *Leishmania braziliensis* complex. In JA Rioux, *Leishmania Taxonomy and Phylogeny*, IMEEE, Montpellier, p. 179-183.

Silveira FT, Ishikawa EA, De Souza AA, Lainson R 2002. An outbreak of cutaneous leishmaniasis among soldiers in Belem, Para State, Brazil, caused by Leishmania (Viannia) lindenbergi n. sp. A new leishmanial parasite of man in the Amazon region. *Parasite 9*: 43-50.

Silveira FT, Lainson R, Shaw JJ, Garcez LM, Souza AA, Braga RR, Ishikawa EA 1990. [Experimental skin leishmaniasis: II--course of the infection in the Cebus apella primate (Cebidae) caused by Leishmania (V.) braziliensis and L. (L.) amazonensis]. *Rev Soc Bras Med Trop 23*: 5-12.

Silveira L, Ribeiro V 2005. Grupo de adesão ao tratamento: espaço de "ensinagem" para profissionais de saúde e pacientes. *Interface (Botucatu) 9*

Simpson L 1987. The mitochondrial genoma of Kinetoplastid protozoa: genomic organization, transcription, replication and evolution. *Annual Review of Microbiology 41*: 363-382.

SINAN 2003. Leishmaniose Tegumentar - Brasil*. Frequência por UF de residência e faixa etária*. Sistema de Informação e Agravos de Notificação Compulsória, Ministério da Saúde, Brasília.

Soto J, Buffet P, Grogl M, Berman J 1994. Successful treatment of Colombian cutaneous leishmaniasis with four injections of pentamidine. *Am J Trop Med Hyg 50*: 107-111.

Soto-Mancipe J, Grogl M, Berman JD 1993. Evaluation of pentamidine for the treatment of cutaneous leishmaniasis in Colombia. *Clin Infect Dis 16*: 417-425.

Southern EM 1975. Detection of specific sequences among DNA fragments separated by gel electrophoresis. *J Mol Biol 98*: 503-517.

Stamenkovic G, Guduric J, Velickovic Z, Skerl V, Krtolica K, Veljkovic E, Dimitrijevic B 2001. Analysis of 5' non-coding region in hepatitis C virus by single-strand conformation polymorphism and low-stringency single specific primer PCR. *Clin Chem Lab Med 39*: 948-952.

Tallab TM, Bahamdam KA, Mirdad S, Johargi H, Mourad MM, Ibrahim K, el Sherbini AH, Karkashan E, Khare AK, Jamal A 1996. Cutaneous leishmaniasis: schedules for intralesional treatment with sodium stibogluconate. *Int J Dermatol 35*: 594-597.

Teodoro U, Alberton D, Kuhl JB, dos Santos ES, dos Santos DR, dos Santos AR, Oliveira O, Silveira TG, Lonardoni MV 2003. [Ecology of Lutzomyia (Nyssomyia) whitmani in an urban area in Maringa, Parana, Brazil]. *Rev Saude Publica 37*: 651-656.

Thakur CP, Kumar K 1990. Efficacy of prolonged therapy with stibogluconate in post kala-azar dermal leishmaniasis. *Indian Journal Medical Research 91*: 144-148.

Thakur CP, Kumar M, Pandey AK 1991. Comparison of regimes of treatment of antimony-resistant kala-azar patients: a randomized study. *Am J Trop Med Hyg 45*: 435-441.

Thomas-Soccol V, Lanotte G, Rioux JA, Pratlong F, Martini-Dumas A, Serres E 1993. Monophyletic origin of the genus *Leishmania* Ross, 1903. *Annales de Parasitologie Humaine et Comparée 68*: 107-108.

Tibayrenc M, Neubauer K, Barnabe C, Guerrini F, Skarecky D, Ayala FJ 1993. Genetic characterization of six parasitic protozoa: parity between random-primer DNA typing and multilocus enzyme electrophoresis. *Proc Natl Acad Sci U S A 90*: 1335-1339.

Torre-Cisneros J, Prada JL, Villanueva JL, Ververde F, Sanchez-Guijo P 1994. Sucessful treatment of antimony-resistant cutaneous leishmaniasis with liposomal amphotericin B. *Clinical Infectious Diseases 178*: 1024-1025.

Ulrich N 1998. Speciation of antimony(III), antimony(V) and trimethylstiboxide by ion chromatography with inductively coupled plasma atomic spectrometric and mass spectrometric detection. *Anal Chim Acta 359*: 245-253.

Upcroft P 1994. Multiple drug resistance in the pathogenic protozoa. *Acta Trop 56*: 195-212.

Vago A, Andrade L, Leite A, Reis D, Macedo A, Adad S, Tostes Jr S, Moreira M, Brasileiro Filho G, Pena S 2000. Genetic Characterization of *Trypanosoma cruzi* directly from tissue of patients with chronic chagas disease. *Am J Pathol 156*: 1805-1809.

Vago AR, Macedo AM, Oliveira RP, Andrade LO, Chiari E, Galvão LMC, Reis DA, Pereira MES, Simpson AJG, Tostes Jr. S, Pena SDJ 1996. Kinetoplast DNA signatures of *Trypanosoma cruzi* strains obtained directly from infected tissues. *American Journal of Pathology 149*: 2153-2159.

Van Belkum A 1995. Low-stringency single specific primer PCR, DNA sequencing and single-strand conformation polymorphism of PCR products for identification of genetic variants of human papillomavirus type 16. *J Virol Methods 43*: 233-239.

Veiga JP, Wolff ER, Sampaio RN, Marsden PD 1983. Renal tubular dysfunction in patients with mucocutaneous leishmaniasis treated with pentavalent antimonials. *Lancet 2*: 569.

Vermeire E, Hearnshaw H, Van Royen P, Denekens J 2001. Patient adherence to treatment: three decades of research. A comprehensive review. *J Clin Pharm Ther 26*: 331-342.

Victoir K, Banuls AL, Arevalo J, Llanos-Cuentas A, Hamers R, Noel S, De Doncker S, Le Ray D, Tibayrenc M, Dujardin JC 1998. The gp63 gene locus, a target for genetic characterization of Leishmania belonging to subgenus Viannia. *Parasitology 117*: 1-13.

Vieira FA 2003. Desenvolvimento de uma metodologia para determinação de trimetil antimônio, Sb(V) e Sb(III) em amostras clínicas por IC-ICPMS*. Departamento de Química*. Pontifícia Universidade Católica do Rio de Janeiro, Rio de Janeiro, p. 110p.

Walton BC, Chinel LV, Eguia y Eguia O 1973. Onset of espundia after many years of occult infection with *Leishmania braziliensis*. *American Journal Tropical Medicine Hygiene 22*: 696-698.

Weigle KA, Labrada LA, Lozano C, Santrich C, Barker DC 2002. PCR-Based Diagnosis of Acute and Chronic Cutaneous Leishmaniasis Caused by Leishmania (Viannia). *J Clin Microbiol 40*: 601-606.

Wirth DF, McMahon Pratt D 1982. Rapid identification of Leishmania species by specific hybridization of kinetoplast DNA in cutaneous lesions. *Proc Natl Acad Sci U S A 79*: 6999-7003.

World Health Organization 1984. The Leishmaniases. World Health Organization.

World Health Organization 1990. Control of Leishmaniases: report of a WHO Expert Committee. World Health Organization, Geneva, p. 1-158.

World Health Organization 2001. Letter to Aventis Pharma Drug Regulatory Affairs, Europe. Essential Drugs and Medicines Policy - W.H.O.

World Health Organization 2003. Adherence a long-term therapies: evidence for action. http://www.who.int/chronic_conditions/en/adherence_report.pdf.

Wyler DJ, Weinbaum FI, Herrod HR 1979. Characterization of in vitro proliferative responses of human lymphocytes to leishmanial antigens. *J Infect Dis 140*: 215-221.

Yarbuth AL, Anez N, Pena YP, Burguera JL, Burguera A 1994. Antimony determination in tissues and serum of hamsters infected with meglulmine antimoniate. *Annals Tropical Medicine Parasitology 88*: 37-41.

Zhang X, Cornelis R, Mees L 1998. Speciation of Antimony (III) and Antimony (V) Species by using High Performance Liquid Chromatography coupled to Hydride Generation Atomic Absorption Spectrometry. *J Anal At Spectrom 13*: 205-207.

Zheng J, Ohata M, Furuta N 2000a. Antimony speciation in environmental samples by using HPLC-ICP-MS. *Analytical Science 16*: 75-80.

Zheng J, Ohata M, Furuta N 2000b. Studies on the speciation of inorganic and organic antimony compounds in airborne particulate matter by HPLC-ICP-MS. *Analyst* *125*: 1025-1028.

ANEXO

Termo de consentimento livre e esclarecido

INSTITUIÇÃO: Instituto de Pesquisa Clínica Evandro Chagas - Fiocruz

COORDENADOR DA PESQUISA: Armando de Oliveira Schubach

ENDEREÇO: Av. Brasil 4365 - Manguinhos - Rio de Janeiro - RJ - CEP 21040-900

TELEFONES: (0xx21) 3865-9525 / 3865-9541 / FAX (0xx21) 3865-9541

NOME DO PROJETO DE PESQUISA:
Ensaio clínico fase III para Leishmaniose Tegumentar Americana. Equivalência entre o esquema padrão e alternativo com antimoniato de meglumina

NOME DO VOLUNTÁRIO: ____

Este documento procura esclarecê-lo sobre o problema de saúde em estudo e sobre a pesquisa que será realizada, prestando informações, detalhando os procedimentos e exames, benefícios, inconvenientes e riscos potenciais.

A leishmaniose tegumentar americana (LTA) é uma doença causada por parasitos chamados Leishmanias e que se apresenta como feridas na pele de difícil cicatrização. Algumas vezes, a LTA pode se tornar mais grave, envolvendo as mucosas de revestimento interno do nariz e da garganta, mesmo vários anos após a cicatrização da ferida na pele. Atualmente, não temos como saber qual paciente adoecerá de novo e qual permanecerá curado definitivamente.

No Brasil, o Ministério da Saúde (MS) recomenda tratar os pacientes com LTA com antimoniato de meglumina em altas doses (20mg por kilograma de peso por dia) durante 20 a 30 dias, respeitando o limite máximo de 3 ampolas diárias.. Entretanto, alterações nos rins, coração, fígado, pâncreas e no sangue são freqüentes. Além de dores nas juntas e desconforto no local de aplicação das injeções por via intramuscular.

No Centro de Referência em Leishmanioses (CRLeish) do IPEC, Fiocruz, uma dose baixa de antimoniato de meglumina (5 mg por kilograma de peso por dia) tem se revelado eficaz e bem tolerada no tratamento de pacientes com LTA. Os pacientes com a forma cutânea são

tratados por 30 dias. Os pacientes com forma mucosa são tratados continuamente, por um mínimo de 30 dias, preferencialmente sem interrupção, até a cicatrização das mucosas, o que costuma ocorrer entre 30 e 90 dias de tratamento. Pacientes idosos ou com outras doenças associadas são tratados com doses baixas em séries de 10 dias com intervalos de 10 dias sem medicação. Pacientes que apresentem contra-indicação para receber o tratamento por via intramuscular ou que apresentem sinais de intoxicação durante o tratamento, poderão ser tratados com uma ou duas aplicações de antimoniato de meglumina diretamente na lesão de pele.

Nossa experiência acumulada sugere que os esquemas de tratamento alternativos apresentam os mesmos bons resultados que o esquema padrão recomendado pelo MS, porém, com menos efeitos adversos. Entretanto, somente após a conclusão deste estudo, poderemos sugerir ao MS que altere as recomendações para o tratamento da LTA.

Agora que o seu diagnóstico de LTA foi confirmado, você está sendo convidado a participar de uma investigação clínica a ser realizada no IPEC-Fiocruz, com os seguintes objetivos:

- ✓ Avaliar a resposta ao tratamento da LTA com o uso de diferentes doses ou formas de aplicação de antimoniais
- ✓ Descrever o comportamento dos antimoniais no corpo humano de acordo com os diferentes esquemas de tratamento
- ✓ Comparar a resposta imunológica de pacientes tratados com os diferentes esquemas
- ✓ Caracterizar os isolados de *Leishmania* e verificar a sensibilidade ao antimonial

A sua participação neste estudo é voluntária. Você poderá recusar-se a participar de uma ou todas as etapas da pesquisa ou, mesmo, se retirar dela a qualquer momento, sem que este fato lhe venha causar qualquer constrangimento ou penalidade por parte da Instituição. O seu atendimento médico não será prejudicado caso você decida não participar ou caso decida sair do estudo já iniciado. Os seus médicos poderão também interromper a sua participação a qualquer momento, se julgarem conveniente para a sua saúde.

A sua participação com relação ao Projeto consiste em autorizar que a indicação do seu tratamento para forma cutânea de LTA, com antimoniato de meglumina por via intramuscular, seja feita por sorteio para um dos seguintes grupos: 1) dose alta por 20 dias contínuos; 2) dose baixa por 30 dias contínuos. Caso haja alguma contra-indicação para você receber qualquer desses esquemas ou intolerância com necessidade de interromper um esquema iniciado, o tratamento será realizado com uma ou duas aplicações da medicação, com um intervalo de duas semanas, diretamente na lesão de pele. Em caso de forma mucosa você poderá ser sorteado para um dos seguintes grupos: 1) dose alta por 30 dias; 2) dose baixa diariamente até a cura. Caso haja alguma intolerância com necessidade

de interromper um dos esquemas iniciado, o tratamento será realizado com dose baixa em séries de 10 dias com intervalos de descanso, seguidamente até a cura. Os médicos que irão avaliar o seu tratamento não saberão qual o esquema utilizado e você não saberá se estará tratando com dose alta ou baixa, para não serem influenciados no julgamento.

Também será necessária a sua autorização: 1) para a utilização de documentação fotográfica ou filmagem de suas lesões para estudo; 2) para que parte do material coletado periodicamente para a realização de exames para acompanhamento da evolução da sua doença, assim como os resultados destes exames de rotina e do seu tratamento sejam utilizados neste estudo; 3) para que parte das amostras coletadas seja estocada a fim de servir para outros estudos que tenham como finalidade a melhor compreensão da doença, o desenvolvimento e avaliação de novos métodos diagnósticos; avaliação da resposta ao tratamento etc., desde que tal estudo seja previamente analisado e autorizado por um Comitê de Ética em Pesquisa.

Participando deste estudo você terá algumas responsabilidades: seguir as instruções do seu médico; comparecer à unidade de saúde nas datas marcadas; e relatar a seu médico todas as reações que você apresentar durante o tratamento, tanto positivas quanto negativas. Os exames e procedimentos aplicados lhe serão gratuitos. Você receberá todos os cuidados médicos adequados para a sua doença. Caso você necessite de atendimento médico, durante o período em que estiver participando do estudo, mesmo fora do seu agendamento, procure o Instituto de Pesquisa Clínica Evandro Chagas - Fiocruz. Em caso de necessidade ligue para o Dr. Armando de Oliveira Schubach, Dra. Cláudia Maria Valete Rosalino ou Dra. Maria Inês Pimentel nos telefones acima. Caso você apresente qualquer problema que necessite de internação, a equipe médica providenciará seu leito no Instituto de Pesquisa Clínica Evandro Chagas - Fiocruz.

Sua identidade será mantida como informação confidencial. Os resultados do estudo poderão ser publicados sem revelar a sua identidade e suas imagens poderão ser divulgadas desde que você não possa ser reconhecido. Entretanto, se necessário, os seus registros médicos estarão disponíveis para consulta para a equipe envolvida no estudo, para o Comitê de Ética em Pesquisa, para as Autoridades Sanitárias e para você.

Você pode e deve fazer todas as perguntas que julgar necessárias antes de concordar em participar do estudo, assim como a qualquer momento durante o tratamento. O seu médico deverá oferecer todas as informações necessárias relacionadas à sua saúde, aos seus direitos, e a eventuais riscos e benefícios relacionados à sua participação neste estudo.

Inconvenientes e riscos principais conhecidos até os dias atuais: O medicamento antimoniato de meglumina costuma causar efeitos indesejáveis, não deve ser utilizados na gravidez e seu uso em mulheres em idade reprodutiva deve ser acompanhado de uso de

método anticoncepcional eficaz como preservativo de látex masculino ou feminino ("camisinha"), diafragma feminino ou anticoncepcional oral ("pílula").

Formas de ressarcimento: Sempre que necessário, nos dias de seu atendimento, poderá ser fornecida alimentação conforme rotina do Serviço de Nutrição e Serviço Social do IPEC para pacientes externos.

Benefícios esperados: Espera-se que, ao final do tratamento, você esteja curado da LTA, embora as consultas de retorno por vários anos após o tratamento sejam necessárias para a confirmação da cura. Os resultados deste estudo poderão não beneficiá-lo diretamente, mas no futuro, poderão beneficiar outras pessoas, pois espera-se que este estudo contribua para que o acompanhamento do tratamento de pacientes com LTA possa ser feito de forma mais eficaz e segura.

Declaro que li e entendi todas as informações referentes a este estudo e que todas as minhas perguntas foram adequadamente respondidas pela equipe médica, a qual estará à disposição para responder minhas perguntas sempre que eu tiver dúvidas.

Recebi uma cópia deste termo de consentimento e pelo presente consinto, voluntariamente, em participar deste estudo de pesquisa.

____ ___________

Nome paciente: Data

____ ___________

Nome médico: Data

ANEXO (TERMO DE CONSENTIMENTO PARA PROCEDIMENTOS DIAGNÓSTICOS)

Termo de consentimento livre e esclarecido

INSTITUIÇÃO: Instituto de Pesquisa Clínica Evandro Chagas - Fiocruz
COORDENADOR DA PESQUISA: Armando de Oliveira Schubach
ENDEREÇO: Av. Brasil 4365 - Manguinhos - Rio de Janeiro - RJ - CEP 21040-900
TELEFONES: (0xx21) 3865-9525 / 3865-9541 / FAX (0xx21) 3865-9541
NOME DO PROJETO DE PESQUISA:
Estudo para a sistematização do atendimento de pacientes com Leishmaniose Tegumentar Americana no Centro de Referência em LTA - Instituto de Pesquisa Clínica Evandro Chagas - Fiocruz
NOME DO VOLUNTÁRIO: ____

A leishmaniose tegumentar americana (LTA) é uma doença que atinge seres humanos e animais, incluindo o cão, causada por parasitos chamados Leishmanias. A doença é transmitida pelo "mosquito palha", que vive em regiões de mata, plantações de banana, manga etc. localizadas próximas às moradias humanas, onde costuma entrar para se alimentar de sangue de pessoas e animais domésticos. A LTA se apresenta como feridas na pele de difícil cicatrização. Algumas vezes, a LTA pode se tornar mais grave, envolvendo as mucosas de revestimento interno do nariz e da garganta, mesmo vários anos após a cicatrização da ferida na pele. Atualmente, não temos como saber qual paciente adoecerá de novo e qual permanecerá curado definitivamente.

Outras doenças como infecções por bactérias, tuberculose, sífilis, esporotricose, outras micoses, tumores etc. podem se manifestar de forma parecida com a leishmaniose e precisam ser diferenciadas para que se possa iniciar o tratamento correto. Entretanto, com os exames existentes atualmente, nem sempre se consegue ter certeza absoluta sobre qual a doença em questão.

No momento, várias perguntas precisam ser respondidas como: de que outras maneiras a LTA pode se manifestar? como se comportam os exames de laboratório antes, durante e após o tratamento? quais pacientes, mesmo após o tratamento, irão reabrir suas cicatrizes ou irão desenvolver doença dentro do nariz ou na garganta? que outras doenças parecidas estão sendo confundidas com a LTA e quais exames devem ser utilizados para esclarecimento? qual o papel dos seres humanos como reservatórios da doença? quais as melhores formas de tratamento? que medidas devem ser tomadas para controlar o problema?

Pelo presente documento, você está sendo convidado(a) a participar de uma investigação clínica a ser realizada no IPEC-Fiocruz, com os seguintes objetivos:

- ✓ Descrever aspectos da LTA: manifestações clínicas e exames de laboratório, tentando estabelecer padrões de apresentação da doença e seu modo de evolução, comparando com outras doenças.
- ✓ Avaliar o uso dos antimoniais e outras drogas utilizadas no tratamento da LTA levando em consideração o tempo de tratamento, toxicidade, facilidade de administração, custo e ausência de envolvimento das mucosas do nariz e da garganta.
- ✓ Isolar, identificar e comparar as leishmanias causadoras da LTA provenientes de diversas localidades.

Este documento procura esclarecê-lo sobre o problema de saúde em estudo e sobre a pesquisa que será realizada, prestando informações, detalhando os procedimentos e exames, benefícios, inconvenientes e riscos potenciais.

A sua participação neste estudo é voluntária. Você poderá recusar-se a participar de uma ou todas as etapas da pesquisa ou, mesmo, se retirar dela a qualquer momento, sem que este fato lhe venha causar qualquer constrangimento ou penalidade por parte da Instituição. O seu atendimento médico não será prejudicado caso você decida não participar ou caso decida sair do estudo já iniciado. Os seus médicos poderão também interromper a sua participação a qualquer momento, se julgarem conveniente para a sua saúde.

A sua participação com relação ao Projeto consiste em autorizar a realização de uma série de exames para o diagnóstico da sua doença, e que parte deste material, assim como os resultados destes exames de rotina, sejam utilizados neste estudo. Também será necessária a sua autorização: 1) para a utilização de documentação fotográfica ou filmagem de suas lesões para estudo 2) para que parte do material coletado periodicamente para a realização de exames para acompanhamento da evolução da sua doença, assim como os resultados destes exames de rotina e do seu tratamento sejam utilizados neste estudo 3) para que parte das amostras coletadas seja estocada a fim de servir para outros estudos que tenham como finalidade a melhor compreensão da doença, o desenvolvimento e avaliação de novos métodos diagnósticos; avaliação da resposta ao tratamento etc., desde que tal estudo seja previamente analisado e autorizado por um Comitê de Ética em Pesquisa.

Os exames e procedimentos aplicados lhe serão gratuitos. Você receberá todos os cuidados médicos adequados para a sua doença.

Participando deste estudo você terá algumas responsabilidades: seguir rigorosamente as instruções do seu médico; comparecer à unidade de saúde nas datas marcadas; relatar a seu médico todas as reações que você apresentar durante o tratamento, tanto positivas quanto negativas.

Caso você necessite de atendimento médico, durante o período em que estiver participando do estudo, procure o Instituto de Pesquisa Clínica Evandro Chagas - Fiocruz, mesmo fora do seu agendamento. Em caso de necessidade ligue para o Dr. Armando de Oliveira Schubach, Dra. Fátima Conceição-Silva ou Dra. Mariza Salgueiro nos telefones acima. Caso você apresente qualquer quadro clínico que necessite de internação, a equipe médica providenciará seu leito no Instituto de Pesquisa Clínica Evandro Chagas - Fiocruz. Seus animais com suspeita de LTA poderão ser atendidos gratuitamente pela médica veterinária Dra. Tânia Maria Valente Pacheco no Serviço de Zoonoses do IPEC.

Sua identidade será mantida como informação confidencial. Os resultados do estudo poderão ser publicados sem revelar a sua identidade e suas imagens poderão ser divulgadas desde que você não possa ser reconhecido. Entretanto, se necessário, os seus registros médicos estarão disponíveis para consulta para a equipe envolvida no estudo, para o Comitê de Ética em Pesquisa, para as Autoridades Sanitárias e para você.

Você pode e deve fazer todas as perguntas que julgar necessárias antes de concordar em participar do estudo, assim como a qualquer momento durante o tratamento. O seu médico deverá oferecer todas as informações necessárias relacionadas à sua saúde, aos seus direitos, e a eventuais riscos e benefícios relacionados à sua participação neste estudo.

Procedimentos, exames e testes que serão utilizados:

Antes do tratamento haverá coleta de informações sobre a doença; exame médico geral e exame da pele com descrição e documentação fotográfica ou filmagem das lesões; exame interno do nariz e da garganta com um aparelho chamado fibra ótica, que permite ver lesões pequenas ou em locais de difícil acesso, para descrição e documentação fotográfica ou filmagem das lesões (se necessário será aplicado "spray" anestésico local). Retirada, com anestesia local, de um pequeno fragmento da lesão de pele, de mucosa ou de "íngua" para realização de exames tanto para diagnóstico (aspecto microscópico do tecido doente e culturas para tentativa de isolamento de possíveis agentes de doença como fungos, bactérias e leishmanias) quanto para pesquisa (identificação de células e outros componentes da resposta inflamatória, assim como novos métodos de identificação dos possíveis agentes da doença). Outros materiais também poderão ser coletados na tentativa de isolamento do agente causador da doença: aspiração com seringa e agulha do bordo da lesão e de secreções em lesões de pele fechadas.

Outros exames também serão realizados para diagnosticar outras doenças possíveis de serem confundidas com a LTA, para classificar a gravidade da doença e avaliar os efeitos dos medicamentos a serem utilizados durante o seu tratamento: um a quatro testes cutâneos (injeção da décima parte de um mililitro de um reativo para determinada doença na pele da região anterior do antebraço, a qual deverá ser revista entre 2 a 3 dias após a

injeção); exames de sangue (quantidade equivalente a aproximadamente três colheres de sopa), exame de saliva (coletada com um tipo de cotonete), radiografia dos pulmões e da face (se necessário complementada por tomografia computadorizada); e eletrocardiograma.

O tratamento da LTA em pacientes humanos costuma ser com o medicamento antimoniato de meglumina por via intramuscular (IM), intravenosa (IV) uma injeção ao dia, geralmente, durante um período de 30 dias contínuos ou com intervalos de descanso. Excepcionalmente, para idosos, pacientes com doenças graves ou que não tolerem o tratamento normal, poderá ser utilizada a via intralesional (IL). O tempo do tratamento poderá ser diminuído ou aumentado conforme a necessidade. Outras opções de tratamento são a anfotericina B (IV) e a pentamidina (IM), ambas injetáveis e necessitando medidas de acompanhamento parecidas com as do antimoniato de meglumina.

Após o início do tratamento, você deverá comparecer a aproximadamente três consultas dentro de 10, 20 e 30 dias. Caso as lesões não cicatrizem totalmente, o tratamento poderá ser continuado pelo período de tempo necessário. Ao se atingir a cura clínica, você deverá retornar para consulta de reavaliação em 1, 3, 6, 9 e 12 meses após o término do tratamento. E, a partir de então, pelo menos uma vez por ano durante um prazo indefinido (no mínimo 5 anos).

A cada retorno deverão ser realizados avaliação médica e exames de sangue (na quantidade aproximada de uma ou duas colheres de sopa) para avaliar os efeitos dos medicamentos utilizados no seu tratamento e/ou para avaliar a evolução da doença. Outros exames, como o eletrocardiograma durante o tratamento, poderão ser realizados quando indicados.

Inconvenientes e riscos principais conhecidos até os dias atuais:

A coleta de sangue poderá causar alguma dor no momento da punção venosa e, eventualmente, poderá haver a formação de uma área arroxeada no local, que voltará ao normal dentro de alguns dias.

Ocasionalmente, os testes na pele poderão, apresentar uma reação forte com inflamação do local, formação de bolhas e, mais raramente, formação de ferida. Todo o processo costuma regredir dentro de alguns dias a poucas semanas.

Tanto os testes na pele quanto o anestésico injetado no momento da biópsia (retirada de um pequeno fragmento de pele para exame) poderão causar alergia, geralmente limitada ao aparecimento de áreas vermelhas, empoladas e com coceira na pele e que respondem bem a medicamentos anti-alérgicos. Mais raramente poderá haver uma reação mais severa com dificuldade de respirar e necessidade de cuidados mais intensos, existentes no IPEC.

No local da biópsia poderá ocorrer inflamação e dor, acompanhados ou não de infecção por bactérias. Caso isso ocorra, poderá ser necessário o uso de medicamentos para dor e antibióticos.

O medicamentos antimoniato de meglumina e pentamidina costumam causar efeitos indesejáveis, não devem ser utilizados na gravidez e seu uso em mulheres em idade reprodutiva deve ser acompanhado de uso de método anticoncepcional eficaz como preservativo de látex masculino ou feminino ("camisinha"), diafragma feminino ou anticoncepcional oral ("pílula"). Quando o tratamento não puder ser adiado, a anfotericina B poderá ser utilizada na gravidez. Os exames com raios-x também não devem ser realizados em grávidas.

Formas de ressarcimento:

Sempre que necessário, nos dias de seu atendimento, poderá ser fornecida alimentação conforme rotina do Serviço de Nutrição e Serviço social do IPEC para pacientes externos.

Benefícios esperados:

Espera-se que, ao final do tratamento, você esteja curado da LTA, embora as consultas de retorno por vários anos após o tratamento sejam necessárias para a confirmação da cura. Os resultados deste estudo poderão ou não beneficiá-lo diretamente, mas no futuro, poderão beneficiar outras pessoas, pois espera-se também que este estudo contribua para que o diagnóstico e acompanhamento do tratamento de pacientes com LTA possa ser feito de forma mais eficaz e segura.

Caso a sua investigação demonstre outro diagnóstico diferente de LTA, você será devidamente orientado a buscar o tratamento mais adequado para o seu caso.

Declaro que li e entendi todas as informações referentes a este estudo e que todas as minhas perguntas foram adequadamente respondidas pela equipe médica, a qual estará à disposição para responder minhas perguntas sempre que eu tiver dúvidas.

Recebi uma cópia deste termo de consentimento e pelo presente consinto, voluntariamente, em participar deste estudo de pesquisa.

______________________________	____________
Nome paciente:	Data
______________________________	____________
Nome médico:	Data
______________________________	____________
Nome testemunha[1]:	Data
______________________________	____________
Nome testemunha[2]:	Data

[1] Apenas no caso de pacientes impossibilitados de manifestar o seu consentimento por escrito.
No caso de menores de 18 anos, deverá ser assinado pelo pai, mãe ou responsável legal.

Comitê de Ética em Pesquisa

PARECER CONSUBSTANCIADO

Protocolo 0055.0.009.000-07

Identificação:

Título do Projeto: "Ensaio clínico fase III para leishmaniose tegumentar americana. Equivalência entre o esquema padrão e alternativos com antimoniato de meglumina".

Subprojeto A: "Ensaio clínico controlado, randomizado, duplo-cego e de fase III para verificar a equivalência da efetividade e comparar a segurança entre o esquema padrão e alternativo com antimoniato de meglumina no tratamento da leishmaniose cutânea"

Pesquisador responsável: Armando de Oliveira Schubach.

Instituição Responsável: Instituto de Pesquisa Clínica Evandro Chagas / Fiocruz.

Data de Apresentação ao CEP: 25/09/2007.

Em adição ao Parecer Consubstanciado datado de 17 de Outubro de 2007, informamos que em 2008 houve informação adicional de algumas mudanças relacionadas ao Subprojeto A:

1) Modificação de ensaio de equivalência para ensaio de não-inferioridade com margem de 15%;

2) Total de pacientes a serem incluídos neste subprojeto será de 72 participantes;

3) Os pacientes serão incluídos em dois grupos de tratamento: 20 mg Sb^{5+}/kg/dia por 20 dias e 5 mg Sb^{5+}/kg/dia por 30 dias.

Neste sentido, esclarecemos que, a partir da data em que foi redigido o Parecer Consubstanciado, esta adição não foi devidamente informada.

Assim, solicitamos que a modificação final do Parecer Consubstanciado já referido, incorpore os dados a partir de 01/09/2008.

O Parecer Consubstanciado de 2008 passa a ter a seguinte conotação:

Léa Camillo Coura

Drª Léa Ferreira Camillo-Coura
Coordenadora do Comitê
de Ética em Pesquisa
Mat. SIAPE 003709620
IPEC / FIOCRUZ

Ministério da Saúde

FIOCRUZ
Fundação Oswaldo Cruz
INSTITUTO DE PESQUISA CLÍNICA EVANDRO CHAGAS

I P E C

Comitê de Ética em Pesquisa

PARECER CONSUBSTANCIADO

Protocolo 0055.0.009.000-07

1. **Identificação:**

Título do Projeto: "Ensaio clínico fase III para leishmaniose tegumentar americana. Equivalência entre o esquema padrão e alternativos com antimoniato de meglumina".

Subprojeto A: "Ensaio clínico controlado, randomizado, duplo-cego e de fase III para verificar a equivalência da efetividade e comparar a segurança entre o esquema padrão e alternativo com antimoniato de meglumina no tratamento da leishmaniose cutânea"

Pesquisador responsável: Armando de Oliveira Schubach.

Instituição Responsável: Instituto de Pesquisa Clínica Evandro Chagas / Fiocruz.

Data de Apresentação ao CEP: 25/09/2007.

2. **Sumário:**

Trata-se de emenda ao Subprojeto A do projeto principal, com modificação do estudo de equivalência para estudo de não-inferioridade com margem de 15%.

Este subprojeto constitui um ensaio clínico controlado com o tratamento padrão, randomizado e de fase III com 72 pacientes com Leishmaniose cutânea (LC) atendidos no Centro de Referência em Leishmaniose – IPEC / Fiocruz. Os indivíduos elegíveis e que concordarem em participar serão alocados aleatoriamente em um dos dois grupos de tratamento: 20 mg Sb^{5+}/kg/dia por 20 dias e 5 mg Sb^{5+}/kg/dia por 30 dias. Tem como objetivo principal comparar os esquemas com antimoniato de meglumina recomendados no Brasil para Leishmaniose Tegumentar Americana (LTA) com o esquema alternativo. Objetivos específicos: comparar a efetividade com margem de não-inferioridade de 15% e a segurança entre os grupos no tratamento de leishmaniose cutânea (LC). O principal benefício potencial deste ensaio, consiste na possibilidade de subsidiar a utilização de doses de antimônio mais baixas, potencialmente menos tóxicas e de menor custo, para o tratamento de leishmaniose cutânea, incluindo pacientes idosos e com co-morbidades (cardiopatias, nefropatias e hepatopatias).

3. **Observações Gerais:** (Atendendo à Resolução CNS 196/96).

Projeto com delineamento adequado. O termo de consentimento livre e esclarecido foi elaborado em linguagem acessível ao sujeito da pesquisa. Este projeto é financiado parcialmente com recursos aprovados pelo edital MCT/CNPq/MS-SCTIE-DECIT 25/2006.

4. Diligências:

Sim. Foram satisfeitas.

5. Parecer: APROVADO.

Data: 01 de setembro de 2008

Assinatura do coordenador:

Léa Camillo Coura

Drª Léa Ferreira Camillo-Coura
Coordenadora do Comitê
de Ética em Pesquisa
Mat. SIAPE 003709620
IPEC / FIOCRUZ
